# Supplementary material for: Eye health knowledge, attitude, and practice among special school managers and barriers to eye health programmes in special schools in Hyderabad, India
Source: PLOS Glob Public Health. 2024 Aug 28;4(8):e0002124. doi: 10.1371/journal.pgph.0002124 (PMC11356432; doi:10.1371/journal.pgph.0002124)
Supplement: S1 Questionnaire — (PDF) [file pgph.0002124.s001.pdf]

KAP Questionnaire for special school managers

Participant age: ____ Years   Participant gender: _________

Participant role/designation in the school _____________________

Type of school: (Special school / Inclusive school/ Neither) __________________

Children with what type of disabilities are admitted in your school? __________________

Knowledge

1. Are you aware of the number of children with eye conditions in your school/class?

   1-Yes ☐   0-No ☐ (If it is No, then ignore question 2)

2. How many children, do you think, have eye problems or wear spectacles in your school/class?

   1-Approximately ☐   0-Not sure ☐

3. What do you think are the chances of children with disability developing eye problems as compared to children without disability?

   3- Very likely ☐   2- Same as children without a disability ☐

   1- Less likely ☐   0- Not sure ☐

4. What are some of the eye conditions you know that are common amongst children with disability?

S.No		Yes - 1	No -0
1	Cataract		
2	Glaucoma		
3	Refractive error		
4	Corneal scar		
5	Squint		
6	Conjunctivitis		
7	Big eyeballs		
8	Small eyeballs		
9	Eye Cancer		
10	Others specify		

5. Do you know eye problems in children, if treated early, can result in better visual outcome?

   2-Yes ☐ 1-Not sure ☐ 0-No ☐

6. What kind of problems, a child with reduced vision likely to experience in the classroom/school? List down a few in the box below.

   ☐

7. How might eye problems in children be identified? List down your answers below in the box.

   ☐

8. Do you know that poor nutrition can cause eye problems in children?

   2-Yes ☐ 1-Not sure ☐ 0-No ☐

9. How often should a child with special educational needs get his/her eyes tested?

   4-Twice a year ☐ 3-Once a year ☐

   2-Only when there is a problem ☐ 1-Not sure ☐

Attitude

1. Do you think a child with a vision problem is less capable to perform academic tasks?

   2-Yes ☐ 1-Not sure ☐ 0-No ☐

2. Do you think the child with vision problem will be able participate in extracurricular activities such as sports and cultural activities?

   2-Yes ☐ 1-Not sure ☐ 0-No ☐

3. Do you think a child with cross eye is likely to have cognitive impairment?

   2-Yes ☐ 1-Not sure ☐ 0-No ☐

4. What do you think are the reasons for development of eye problems in children? List down in the table below.

Heredity [ ] Lack of nutrition [ ] Physical illness [ ]

Trauma [ ] Curse from God [ ] Consanguinity [ ]

Others, specify [ ]

5. Do you think children who are vision impaired (low vision/blind) should attend normal school?

2-Yes [ ] 1-Not sure [ ] 0-No [ ]

6. If yes, then, why do you think visually handicapped (low vision/blind) children should go to normal school? If no, then, why they shouldn't? Write your reason in the box against your answer.

2-Yes [ ]

1-Not sure [ ]

0-No [ ]

7. Do you think a child with vision impairment (low vision/blind) will interact with peers?

1-Yes [ ] 0-No [ ]

8. If yes, why [ ]

0-Not sure [ ]

9. If no, why [ ]

0-Not sure [ ]

Practice

1. What would you do if you see a child keeping books close to his/her face while reading and writing?

2. What do you do with the children identified with eye diseases?

3. What do you do with children identified with eye diseases which cannot be treated?

4. What adaptations do you allow for a child with vision problem in the classroom?

5. Do you organize any eye health programs for the children in your school and how often?

6. What accessibility considerations do you have for the children with impairment to access toilets and classroom?

7. What do you do for providing good nutrition for children in your school?
