# Supplementary material for: Eye health knowledge, attitude, and practice among special school managers and barriers to eye health programmes in special schools in Hyderabad, India
Source: PLOS Glob Public Health. 2024 Aug 28;4(8):e0002124. doi: 10.1371/journal.pgph.0002124 (PMC11356432; doi:10.1371/journal.pgph.0002124)
Supplement: Attachment — Submitted filename: PGPH-D-23-01037_reviewer VRM.pdf [file pgph.0002124.s002.pdf]

Eye health awareness among special school managements and barriers to organize eye health programme in special schools in Hyderabad, India

Commented [VM1]: REPHRASE
You evaluated KAP amongst special school managers and programme barriers and enablers amongst organizations. The Title needs to more accurately affect study.

Author Information

Winston D Prakash [1,2,3]

Priya Morjaria [2]

Ian McCormick [2]

Rohit Khanna [1,4,5]

Affiliations

1. Allen Foster Community Eye Health Research Centre, Gullapalli Pratibha Rao International Centre for Advancement of Rural Eye care, L V Prasad Eye Institute, Hyderabad, Telangana, India
2. International Centre for Eye Health, London School of Hygiene and Tropical Medicine, Keppel Street, London, UK
3. Brien Holden Institute of Optometry and Vision Science, L V Prasad Eye Institute, Hyderabad, Telangana, India
4. School of Optometry and Vision Science, University of New South Wales, Sydney, Australia,
5. University of Rochester, School of Medicine and Dentistry, Rochester, NY, USA

Corresponding author and address:

6. Mr. Winston D Prakash,
7. E-mail: winston@lvpei.org

Abstract

Background

Children with special education needs (SEN) are at higher risk of developing vision problems; however, in India, there is inadequate data on awareness of their visual needs among special school managements (SSM). This study is aimed to identify the awareness level among SSM and barriers/enablers for organizing SEH programmes in special schools as reported by the eye health program organizers.

Commented [VM2]: WRITE IN FULL FOR 1ST USE

Methods

A mixed model qualitative study was conducted between July and August 2020 among SSM and eye health programme organizers from local eye care providers in Hyderabad, India. SSM participants completed an online questionnaire assessing their knowledge, attitude, and practice about the visual needs of children and SEN and eye health programme organizers were interviewed by telephone. The responses were recorded and anonymised for thematic analysis.

Commented [VM3]: MIXED METHOD MODEL INCLUDES QUANTITATIVE AND QUALITATIVE

Deleted: C

Deleted: with

Commented [VM4]: How was quantitative data analysed?

What were the ethical considerations of the study?

Results

13/67 invited SSM (19%) participated, 2/4 invited eye health programme organizers (50%) were interviewed. 92% of SSM participants were aware of vision impaired (VI) children in their schools. Awareness of potential causes of VI ranged from 54%-92% by cause, common eye conditions 23%-69% by conditions and difficulties experienced by children with SEN in classroom activities 46%-77% by activity. Only 23% of SS organized yearly SEH programmes. The eye health programme organizers reported barriers such as lack of interest from SSM, unavailability of qualified screening staff, lack of provision for spectacles, and low vision devices.

Commented [VM5]: Causes are reported in the main study under ATTITUDE and not KNOWLEDGE (Table 3)

Commented [VM6]: Check accuracy as per Table 2.

Conclusion

This study showed low eye awareness levels about eye health needs of children among SSM and there are several barriers to conduct SEH programmes which include lack of school participation, poor availability of human resource as well as treatment provisions. Further research is needed to undertake a comprehensive situational analysis and to plan eye health programs for children with SEN.

Commented [VM7]: ?

Commented [VM8]: What does this mean?

Keywords: Special needs, special school eye health, Eye health awareness, Barriers to organize school eye health programs, Special needs eye health

INTRODUCTION

Children with special educational needs (SEN) are at a higher risk of developing vision impairment (VI) and ocular comorbidities.(1, 2) In India, 28% of people living with a disability are under 19 years of age and 18% of these have a vision related disability.(3) Vision plays an important role in early childhood development such as hand-eye coordination, communication, spatial comprehension and social development.(4-6) Impaired vision can cause difficulty with daily living activities (ADL) and may lead to anxiety and depression,(7) and VI in the presence of other disabilities can amplify these difficulties further. Therefore, early identification of VI and other eye conditions in children with SEN is crucial for better health and development outcomes. (8)

School eye health (SEH) programmes have a potential to provide children with access to good quality services and can be cost-effective.(9-11) However, there is less evidence to what extent they are run in schools for children with SEN. A study by Woodhouse et al. in 2014,(12) found that only 53% of special schools in Wales, UK, had conducted eye health programmes in their schools.

Hyderabad is the capital city of Telangana state in South India and has a population of over 10 million people. There are over one hundred organizations involved in teaching, training, and providing services for children with SEN. According to a local eye health organization involved in delivering community-based eye health services, most SHE programmes are focused only on mainstream schools (V Sarvepally, personal email communication, 28 January 2020 – email communication available). Although studies from outside India report poor awareness level among schoolteachers, studies from within India among mainstream schoolteachers seem to show a better awareness levels.(13-16) The aims of this study were to identify the level of eye health awareness among special school managers and the barriers or enablers to organizing eye health programmes in special schools, from the perspective of eye health programme organizers.

Commented [VM9]: SEH

Commented [VM10]: In relation to?

Commented [VM11]: Were there any such studies conducted amongst school managers (our study target)?

Have there been any studies which showed lack of awareness impacting eye care services in school?

Deleted: of

Deleted: for

METHODS

Study Design and study population

This is a mixed-methods qualitative study that included an online questionnaire and semi-structured interviews by telephone. The heads of special school management team members and programme organisers from eye care organizations involved in community outreach activities in urban and sub-urban areas of Hyderabad, India were included in the study.

Commented [VM12]: ? Questionnaire (quantitative) and interviews (qualitative)

Commented [VM13]: Is this a validated questionnaire?

Commented [VM14]: What was the sampling strategy?

Ethics

Ethics approval was obtained from the London School of Hygiene & Tropical Medicine's MSc Research Ethics Committee, London, UK and the Hyderabad Eye Research Foundation, Hyderabad, India. All participants had to read and sign an informed consent form prior to the data collection.

Commented [VM15]: How was confidentiality maintained, was participation voluntary?

Inclusion and exclusion criteria

Special schools were defined as schools involved in helping children with activities such as communication, interaction, cognition, learning, catering to sensory and physical needs, helping with social and mental health, and were identified from a local tertiary eye care centre's rehabilitation center database (Figure 1). Contact details for all identified special schools in Hyderabad were obtained from the database and a publicly available online resource (www.nayi-disha.org) and schools with valid contact details were invited to participate in the study by a telephone call and an email. Headteachers, directors or principals who are directly involved in school management activities were eligible. Other organizations such as early intervention centres, therapy centres, homes for the disabled and schools for the blind were excluded from this study. Any SEH programme organizers from organizations active in the Hyderabad area with a minimum of 2 years' experience in the role were eligible.

Commented [VM16]: This info should be in the INTRODUCTION SECTION

Commented [VM17]: This is selection criteria and should be included in that part of the Methodology.

This section should merely highlight eligibility and exclusions

Data collection

The data collection was carried out between July-August 2020. Participant information and consent forms (English or Telugu language) were sent via email to each participant on invitation. After obtaining consent, a link to an online questionnaire built with Google Forms was sent to the participants. The questionnaire included demographic details and questions structured to capture knowledge, attitude, and practice with regards to the eye health needs of children studying in their schools. It included multiple-choice questions and open text boxes to allow participants to express their views. Participants were asked to complete the questionnaire within two weeks from receiving but requested not to refer to any readily available resources, so their answers reflected their current understanding. Schools who did not respond to calls or emails after three attempts were considered non-responders. Those who agreed to participate and received the questionnaire but did not complete it within the two-week period were also considered non-responders.

Commented [VM18]: Study

Commented [VM19]: What informed the questions that were included? Were they based on previous studies? How were they validated?

Commented [VM20]: and

Programme organizers were contacted by telephone to arrange an interview. An information and consent form was sent in advance. The interview guide consisted of demographic details and semi-structured questions addressing the barriers and enablers for organizing programmes in special schools. Those who did not respond to an invitation to interview were considered non-responders. None of the participants were given any financial or other incentives for participating. The individual identity of participants from both groups were concealed during data cleaning and analysis for anonymity. However, the email addresses and phone numbers of participants were available with the principal investigator as these participants were contacted through email and telephone calls.

Commented [VM21]: to

Commented [VM22]: These are ethical considerations

Data analysis

Responses to the online questionnaire were downloaded and transferred into a excel spread sheet (Microsoft office 2019). The descriptive statistics was carried out using Microsoft excel spread sheet 2019. Interviews were recorded and transcribed for thematic analysis. The

Commented [VM23]: Why were there no inferential statistics done? Eg. was there a relationship between those with better knowledge and practices such as arranging regular screening programmes?

Commented [VM24]: Were they then coded?

questionnaire responses were categorized under three different themes namely knowledge, attitude, and practice for the analysis.

Commented [VM25]: The KAP part of the study included these three enquiry areas/categories which were analysed quantitatively by my understanding. They did not undergo coding for Thematic analysis as the categories and specific MCQ alternatives were predetermined and part of the questionnaire.

Deleted: K

Commented [VM26]:

RESULTS

Questionnaire for special school management

We identified 67 eligible special schools, of which 39 could be contacted via a valid telephone number or email address. One school was public and 38 were private. All 39 schools agreed to participate, however, representatives from only 13/39 (33%) went on to complete the questionnaire. The median age of the participants was 40 years (interquartile range 35-51.5 years) and there were more females (n=9/13) than males. Eleven schools had less than 50 students while the other two had more than 100 students enrolled. Ten schools were schools for children with more than one type of disability (Table 1).

Table 1: Demographic details of school management participants

Table 1: Demographic details		
Demographics	Median(n=13)	Inter Quartile Range
Age	40	16.5 (35, 51.5)
Sex	Total number of participants	
Male	4 (31%)	
Female	9 (69%)	
Speciality of schools	Total number of schools	
Multiple disability	10 (77%)	
Autism	2 (15%)	
Intellectual disability	1 (8%)	
School strength *	Median(n=13)	Inter Quartile Range
*Total number of children in schools	36	26 (14,40)

Knowledge

Overall, 92% (n=12) of respondents were aware of the number of children with eye conditions in their schools and 77% (n=10) were aware that early treatment could result in better visual outcomes. However, only 23% (n=3) had any knowledge of children's increased risk of developing eye conditions and 63% (n=9) said they identified vision problems by just observing the behaviour of a child in the classroom. 31% (n=4) felt an eye check-up should be considered only if necessary (Table 2).

Commented [VM27]: Do not start a sentence with a number/figure. If necessary, write out in full.

Table 2: Participant response to Knowledge related questions

Table 2	
**Knowledge**	**n=13 (%)**
**Number of children with eye conditions in special schools**	
Aware	12 (92%)
Unaware	1(8%)
**Children SENs chances of developing eye conditions**	
Very likely	3 (23%)
Same as children without disability	3 (23%)
Less likely	2 (15%)
Not sure	5 (39%)
**Commonly known eye conditions**	
Squint	9 (69%)
Big eyeballs	4 (31%)
Refractive error	3 (23%)
Cataract	3 (23%)
Small eyeballs	3 (23%)
Eye cancer	2 (15%
Conjunctivitis	2 (15%)
Glaucoma	1 (8%)
**Observable symptoms in the school & classroom**	
Classroom related activities for Near	10 (77%)
Classroom related activity for distance	6 (46%)
Eye hand coordination	4 (31%)
Outdoor sports	3 (23%)
Identifying objects around	3 (23%)
Mobility	1 (8%)
Others	4 (31%)
Not sure	2 (15%)
**How often is an eye check-up warranted for children with SENs**	
Twice a year	5 (39%)
Once a year	4 (31%)
Only if needed	4 (31%)

Commented [VM28]: rephrase

Commented [VM29]: rephrase to accurately describe the list

Attitude

Most participants (92%; n=12) felt that children with vision problems were less capable of performing academic activities, although 69% (n=9) felt that they would be able to participate in extracurricular activities. Over half of the participants (62%; n=8) felt that children with VI or blindness, without any other additional disability, should attend normal schools as this could improve motivation and self-confidence. While 92% (n=12) said disabled children can interact with non-disabled peers, 31% (n=4) felt that they should not be attending normal school as they require special attention on a regular basis (Table 3).

Commented [VM30]: did you correlate this with the responses of the 62% cited above?

Table 3: Participants response to attitude related questions

Table 3	
**Attitude**	**n = 13 (%)**
**Ability to perform academic tasks**	
Normally capable	1 (8%)
Less capable	12 (92%)
**Ability to participate in non-academic activities**	
Normally capable	9 (69%)
Less capable	1 (8%)
Not sure	3 (23%)
**Reasons for developing eye conditions in children SENs**	
Lack of Nutrition	12 (92%)
Heredity	10 (77%)
Physical illness	9 (69%)
Injury	9 (69%)
Consanguinity	7 (54%)
Curse from God	2 (15%)
**Can children with VI attend mainstream school?**	
Yes	8 (62%)
No	4 (31%)
Not sure	1 (8%)
**VI children's ability to interact with peers**	
Can interact normally	12 (92%)
Did not answer	1 (8%)

Commented [VM31]: Knowledge question? This is how it is reported in ABSTRACT

Practice

Half of the participants (46%; n=6) said that they would refer children having problems seeing in class to an eye doctor, but 23% (n=3) were unsure of what should be done. Half

(46%; n=6) had never organized any eye health programmes in their schools. 54% (n=7) participants stated that they have an additional trained caretaker staff when asked about accessible toilets and classrooms. 15% (n=2) of schools reported having ramps, railings, and support handles, while 39% (n=5) provided a nutritious diet in their schools (Table 4).

Table 4: Participants response to practice related questions

Table 4	
**Practice**	**n=13(%)**
**If a child faces difficulty in reading & writing**	
Refer to eye doctor	6 (46%)
Modify working distance	3 (23%)
Help	1 (8%)
Did not answer	1 (8%)
Others	1 (8%)
**What classroom adaptations are made for VI children?**	
Approach magnification	7 (54%)
Encourage them to use and provide assistive aids	5 (39%)
Give extra time	2 (15%)
Environmental modifications	1 (8%)
Others	7 (54%)
Did not answer	2 (15%)
**If a child is found to have some eye problem**	
Refer to eye doctor	7 (54%)
Provide special care	1 (8%)
Help them wear glasses	1 (8%)
Ask them to stay home until they get better	2 (15%)
Did not answer	2 (15%)
**If a child has an untreatable eye condition**	
Create adaptive environments	4 (31%)
Refer them to doctor/rehabilitation	3 (23%)
Not sure	3 (23%)
Others	3 (23%)
**How often are eye health programmes organised in schools?**	
Once a year	3 (23%)
Twice a year	1 (8%)
Rarely	2 (15%)
Never	6 (46%)
**Available facilities to access toilets and classrooms**	
Extra trained caretaker staff	7 (54%)
Railings, ramps, and support handles	2 (15%)
Comfortable seating	1 (8%)
None	2 (15%)
Did not answer	1 (8%)
**Provision for nutritious diet for children in their schools**	
Educate the parents	6 (46%)
Provide nutritious diet at school	5 (39%)
None	2 (15%)

Commented [VM32]: Did they have poor knowledge or poor attitude?

Commented [VM33R32]:

Commented [VM34]: Attend to sentence structure

Commented [VM35]: Same here

Deleted: are

Did not answer	1 (8%)

Interview with eye health programme organizers

Of four known community outreach organizers from four different organizations in Hyderabad, two responded and agreed to be interviewed. One was a public health specialist with more than 2 decades of experience and the other, an optometrist and head of community ophthalmology services with a decade long experience in the field.

Interview responses

Interviewee responses on the status of SEH programmes in special schools are summarized in Box 1. Overall, the frequency of SEH programmes in special schools seem to be very low. Eye examination included as a part of annual health check-up in these schools is being one of the main reasons reported for the less frequent SEH programmes in special schools. Furthermore, there are no initiatives, neither from the organizing team or from the special schools for conducting SEH programmes in special schools.

Commented [VM36]: What were the main Themes that emerged from the interviews?
Which are the specific codes per theme?

Commented [VM37]: rephrase

**Box 1: Frequency of SEH programmes conducted in special schools**	
Participant 1	<1% out of 50 schools covered in a year were special schools
Participant 2	<2% out of 1000 schools covered in a year were special schools
**Reasons for less coverage**	
Participant 1	Eye health assessment as part of annual check-up is already happening
Participant 2	As eye check-up at the time of admission has already been done there are no further eye health services sought by the school or the parents
**Proactiveness in organizing SEH programmes**	
Participant 1	Organize only if requested by the schools/governmental agencies
Participant 2	Organize as part of initiatives from governmental agencies or NGO's
	Special schools seldom take initiative in organizing SEH programmes

Commented [VM38]: What does Box mean? Need to give a Table Heading. Is Frequency an emergent theme?

Barriers to organizing SEH programmes in special schools are summarized in Box 2. The main barriers included are lack of provision and unaffordability of spectacles and low vision

Commented [VM39]: Do SEH programmes include vision screening with referrals as well as comprehensive vision and ocular health assessments?

devices for children in special schools. One organizer said "The governmental provisions for availing spectacles and low vision devices (LVDs) for free of charge remain only on the policy level and never gets to see the light of the day". Another organizer said, "Future sustainability of SEH programme in special schools will be a major challenge due to lack of enthusiasm from the stakeholders including school managements, parents as well as programme organizers."

Commented [VM40]: Was this a barrier to screening or comprehensive eye care services?

Deleted: "

**Box 2: Barriers for organizing SEH programmes in special schools reported by the organizers**
• Unwillingness of teachers to participate in the screening process as volunteers or for helping the screening team
• Unavailability of skilled manpower (optometrists/ophthalmologists) for special school programmes
• Increased economic cost pertaining to skilled manpower and equipment needed
• Unwillingness/unavailability of parents to participate in programmes
• Lack of provision and unaffordability of spectacles, and LVDs and inaccessibility of governmental aids for purchasing these devices
• School infrastructures are inaccessible
• Currently there are no standard screening protocols available for special school eye health programmes

Existing good practices among the schools and eye health providing teams and points to be considered in the future programmes are summarized in box 3. Medical record keeping among the schools and creating awareness among parents and teachers during the screening process, team member training in handling children with SEN are some of the good practices that are existing among the stakeholders and the screening teams. One organizer stated that "There are no standard protocols for special school eye heath programs, hence this needs to be developed".

Commented [VM41]: Are these the Enablers? Which probe questions were these responses to?

Commented [VM42]: Are these being done by the existing organizations represented in the study?

Commented [VM43]: This is cited in the Table above as a barrier and not an enabler

**Box 3: List of existing good practices and points to be considered in the future programmes**
**From Schools side**
• Medical history record keeping for every child – Helps in understanding the overall medical conditions of children
**From eye care providers side**

Commented [VM44]: Re-phrase

Commented [VM45]: Re-phrase
Are these providers the organizations in Hyderabad or private clinicians?

• Helping the parents understand the eye health needs of their child during eye examination
• Creating awareness among the schoolteachers about the need for eye care services for children with SEN
• Mandatory training of screening team members prior to the programme in examining children with SEN
**Points to be considered prior to organizing SEH programmes in special schools**
• Making sure that the programme venues are accessible
• Inform parents/guardians well in advance and suggest them to be available during the screening
• Develop a standard operating protocol pertaining to special school eye health programmes
• Stakeholder involvement should include local NGOs and volunteers
• Making sure that the provision for spectacles and LVDs are available
• Governments should consider monetary or non-monetary incentives for participating NGOs/organizations

Commented [VM46]: Was this the probe question or the emergent Theme?

DISCUSSION

Universal eye health and the Sustainable Development Goal 3 “to ensure healthy lives for all people at all ages” cannot be achieved without ensuring equitable access to eye health care for underserved groups. This study surveyed the knowledge, attitudes, and practices of heads of schools for children with SEN and interviewed school eye health programme organizers in Hyderabad to understand whether there was a potential service gap for this group of at-risk children.

Encouragingly, almost all participants said they were aware of the number of children in their schools with eye conditions and that early intervention might result in better eye health outcomes. Despite this, only half of the participants said they had organized an eye health programme and only half of them said that they would refer a child having vision problems to an eye doctor. Even though most participants knew that children with SEN needed an eye check-up at least once or twice a year, this happened in less than a third of schools, lower than the 53% reported in a study from Wales, UK in 2014.(12)

Refractive error being one of the most common conditions found among children with SEN,(17, 18) only 23% of participants were aware of this condition. Almost all participants said poor nutrition could cause eye problems, but none of the participants mentioned about

Commented [VM47]: rephrase

vitamin A deficiency (VAD). Although there are not many studies reporting VAD in children with SEN, one study reported low vitamin A levels among Autism spectral disorders from China in 2021.(19) This may also indicate that many of these participants were unaware of the importance of vitamin A for good eye health, which may also directly impact the nutrition that is provided in the schools.

Most participants exhibited a negative attitude towards vision-impaired children's ability to perform academic tasks. This could negatively impact eye health seeking behaviour or referral practices among school staff. Almost two thirds of the participants thought children with only vision impairment could study in mainstream schools assuming that these schools are inclusive. They also felt studying with normal peers could improve their psychosocial well-being. Although this claim is supported by several studies carried out in developed countries (20), the evidence in LMICs is inadequate.(21)

Apart from creating adaptive environments in schools, many participants said they provide assistive devices; however, one cannot justify the reliability and effectiveness of these practices which require certain level of professional training and competency. In addition, they also did not mention about any specific devices pertaining to aid visually impaired. Hence, it is possible that this response could be due to a mere assertion of broader terminology used with regards to any disability and cannot justify the awareness level pertaining to aiding vision related difficulty. The low level of knowledge about the eye health needs of children with SEN among special school heads in Hyderabad is comparable to reports from mainstream primary schools outside India,(14, 15, 22) however, it does not correlate with mainstream primary schools in India.(13, 16) This could be due to higher coverage of mainstream schools during SEH programmes in India compared to special schools, which may also increase the awareness level among mainstream schoolteachers.

Interviews with SEH programme organizers point to various barriers for implementing programmes in special schools. Limited resources were of a major concern: assessing children with SEN requires qualified eye care workers such as optometrists or

Commented [VM48]: was this on the list in the questionnaire?
Could the term "nutrition" have included vitamin deficiencies on the part of the respondents?

Commented [VM49]: Was the contents of the meals provided at schools explored in the study?

Deleted: a

Commented [VM50]: was this asked? If not, it could be a limitation of the questionnaire

Commented [VM51]: As this was not tested it may be presumptious

Commented [VM52]: were the participants also school heads? If they were teachers, the awareness may be better as they have greater interaction with children than heads of schools.

ophthalmologists, with expertise in paediatric and low vision assessment (LVA). Equipment used for LVA and LVDs were noted to be expensive to procure. Furthermore, LVA will be a challenging task on the screening sites due to the various logistical constraints, in addition to being time consuming. Hence, it poses a need for appropriate referral set ups in the proximity for the referred children to avail these services. Appropriate stakeholder engagement in schools and parents was another major concern. Lack of motivation among teachers was reported on the assumption that they are already overburdened with other health programmes and responsibilities. The organizers reported that there is less scope for the screening teams to conduct eye health awareness sessions for the primary stakeholders (parents/guardians), as most of them do not attend the screening programmes. This may also have a significant impact on the continuum of care for children with eye health needs and may further lead to poor referral uptake.

The interviewees made several recommendations to improve knowledge, attitude and practice of stakeholders and to increase uptake of eye health programmes in special schools. They suggested that incorporating health promotion strategies within SEH programmes could make them more impactful and might indirectly address the issue of sustainability. The more informed the stakeholders are about the eye health needs of these children, the more they might proactively take them for regular eye examinations, schools may include eye examination as part of their annual health programmes and teachers may become more aware and sensitive towards the eye health needs of these children. It was also reported that financial protection against the cost of spectacles and LVDs could reduce the burden on families who may already face increased health care costs associated with caring for children with SEN.(23)

Limitations

This study should be interpreted in the context of its limitations. Firstly, the participants recruited were the school heads from a minority of special schools in the Hyderabad area and the participants of these institutions may not be representative of all

Commented [VM53]: Try to separate what organizations need for vision screening at these schools vs the supply of devices for children diagnosed through eye examinations.

Commented [VM54]: rephrase

Commented [VM55]: opportunities?

Deleted: ,

Commented [VM56]: rephrase

Commented [VM57]: what does this mean? Are you suggesting that parents receive subsidies or do not pay at all?

headteachers/schools in the area. Secondly, the study period was interrupted by COVID-19 travel restrictions, meaning face-to-face sensitization and enrolment of headteachers was not possible. Thirdly, although the questionnaire was piloted among the peers of the principal investigator, it was not validated for the target audience, which might have resulted in varying levels of understanding between participants. Finally, the day-to-day engagement of the headteachers with the children in their schools was unverifiable and it might have been valuable to have recruited class teachers for more insight into the daily activities and challenges faced by the children, however, this was hampered by the pandemic which posed difficulty in contacting the class teachers.

Conclusion

Despite the limitations, this study highlights a potentially important service gap for SEH programmes in India. Measuring the eye health awareness level among schoolteachers in a larger sample could give a better insight into the matter. In addition, it proposes a need to include eye health promotion and training of teachers in identifying symptoms of vision impairment to be an integral part of SEH programs in special schools. Further situational analysis should include a survey of prevalence of VI and other eye conditions among children with SEN to understand the current gap and need.

Commented [VM58]: Need to highlight key KAP aspects in relation to the managers and barriers and enablers with key recommendations to overcome barriers.

Acknowledgements

The authors would like to acknowledge the help received from Ms.Mahalaxmi Mojjada for translating the consent form and questionnaire into Telugu language. The authors also would like to thank all the participants for their valuable time for responding to the questionnaire and the interview.

Financial support

This study was carried out as part of MSc thesis at London School of Hygiene and Tropical Medicine (LSHTM), Keppel Street, London, UK. The study was supported by British Council for Prevention of Blindness, LSHTM, and Commonwealth eye health consortium.

Conflicts of interest

There are no conflicts of interests.

References

1. Das M, Spowart K, Crossley S, Dutton GN. Evidence that children with special needs all require visual assessment. Arch Dis Child. 2010;95(11):888-92.

2. Salt A, Sargent J. Common visual problems in children with disability. Arch Dis Child. 2014;99(12):1163-8.

3. Disabled Persons in India a statistical profile 2016 (Cited 2020 April 30) [Available from: http://mospi.nic.in/sites/default/files/publication_reports/Disabled_persons_in_India_2016.pdf].

4. Andersen ES, Dunlea A, Kekelis LS. Blind children's language: resolving some differences. J Child Lang. 1984;11(3):645-64.

5. Hatton DD, Bailey DB, Burchinal MR, Ferrell KA. Developmental Growth Curves of Preschool Children with Vision Impairments. Child Development. 1997;68(5):788-806.

6. Molloy A, Rowe FJ. Manneristic Behaviors of Visually Impaired Children. Strabismus. 2011;19(3):77-84.

7. Kempen GI, Ballemans J, Ranchor AV, van Rens GH, Zijlstra GA. The impact of low vision on activities of daily living, symptoms of depression, feelings of anxiety and social support in community-living older adults seeking vision rehabilitation services. Qual Life Res. 2012;21(8):1405-11.

8. Kulkarni SK, Veer AB, Gaikwad A, Deshpande M. The impact of early intervention on a child with vision loss in infancy - A parental perspective. Indian journal of ophthalmology. 2022;70(9):3377-81.

9. Frick KD, Riva-Clement L, Shankar MB. Screening for refractive error and fitting with spectacles in rural and urban India: cost-effectiveness. Ophthalmic Epidemiol. 2009;16(6):378-87.

10. Lester BA. Comparing the cost-effectiveness of school eye screening versus a primary eye care model to provide refractive error services for children in India. Community Eye Health. 2007;20(61):15.

11. Saravanan S, Krishnamurthy S, Ramani KK, Narayanan A. Cost Analysis of a Comprehensive School Eye Screening Model from India. Ophthalmic epidemiology. 2022:1-7.

12. Woodhouse JM, Davies N, McAvinchey A, Ryan B. Ocular and visual status among children in special schools in Wales: the burden of unrecognised visual impairment. Arch Dis Child. 2014;99(6):500-4.

13. Ambika K, Nair N. A Study on Awareness of Primary School Teachers Regarding Refractive Errors and its Early Identification among Primary School Children. International Journal of Nursing Education. 2013;5:6-9.

14. Habiba U, Ormsby GM, Butt ZA, Afghani T, Asif M. Knowledge and practices of teachers associated with eye health of primary school children in Rawalpindi, Pakistan. Taiwan J Ophthalmol. 2017;7(1):28-33.

15. Maru A, Belete G, Adimassu N. Knowledge, attitude and associated factors among primary school teachers regarding refractive error in school children in Gondar city, Northwest Ethiopia. PLOS ONE. 2018;13:e0191199.

16. Thomas A. A comparative study to assess the knowledge of primary school teachers regarding refractive errors among primary school children in selected urban and rural schools, Mangalore: RajivGandhi University of Health Science. 2013.

17. Gogate P, Soneji FR, Kharat J, Dulera H, Deshpande M, Gilbert C. Ocular disorders in children with learning disabilities in special education schools of Pune, India. Indian J Ophthalmol. 2011;59(3):223-8.

18. Pehere NK, Khanna RC, Marlapati R, Sannapaneni K. Prevalence of ophthalmic disorders among hearing-impaired school children in Guntur district of Andhra Pradesh. Indian Journal of Ophthalmology. 2019;67(4):530-5.

19. Wen J, Yang T, Zhu J, Guo M, Lai X, Tang T, et al. Vitamin a deficiency and sleep disturbances related to autism symptoms in children with autism spectrum disorder: a cross-sectional study. BMC pediatrics. 2021;21(1):299.

20. Price R. Inclusive and special education approaches in Developing countries: (Cited 2020 August 14) Available from: https://assets.publishing.service.gov.uk/media/5c6ac403ed915d4a39787401/373_Inclusive_and_Special_Education_Approaches.pdf. 2018.

21. Kuper, H, Saran, A & White, H 2018, Rapid evidence assessment (REA) of what works to improve educational outcomes for people with disabilities in low- and middle-income countries, Campbell Collaboration, and International Centre for Evidence in Disability, [Oslo], (cited 29 May 2023), Available from: https://www.gov.uk/government/publications/improving-educational-outcomes-for-people-with-disabilities-in-low-and-middle-income-countries-why-does-it-matter-and-what-works.

22. Okoloagu N, Okoye O, Onwubiko S, Eze C, Eze B, Chuka-Okosa C. A Survey of Teachers; Knowledge, Attitudes, and Practices Related to Pupils’ Eye Health and School-Based Eye-Health Services. Nigerian Journal of Ophthalmology. 2019;27(2):68-75.

23. Malvankar-Mehta MS, Wilson R, Leci E, Hatch K, Sharan S. Cost and quality of life of overlooked eye care needs of children. Risk management and healthcare policy. 2018;11:25-33.

Deleted: ¶
¶
