# Supplementary material for: Eye health knowledge, attitude, and practice among special school managers and barriers to eye health programmes in special schools in Hyderabad, India
Source: PLOS Glob Public Health. 2024 Aug 28;4(8):e0002124. doi: 10.1371/journal.pgph.0002124 (PMC11356432; doi:10.1371/journal.pgph.0002124)
Supplement: Attachment — Submitted filename: PLOS PUBLICATION SPECIAL SCHOOLS EYEHEALTH PROGRAMS REVIEWED.pdf [file pgph.0002124.s004.pdf]

PLOS Global Public Health

A cross-sectional survey of eye health knowledge, attitude, and practice among special school managers and barriers to eye health programmes in special schools in Hyderabad, India

--Manuscript Draft--

**Manuscript Number:**	PGPH-D-23-01037R1
**Article Type:**	Research Article
**Full Title:**	A cross-sectional survey of eye health knowledge, attitude, and practice among special school managers and barriers to eye health programmes in special schools in Hyderabad, India
**Short Title:**	Eye health KAP and Barriers to organize eye health program in special schools
**Corresponding Author:**	Winston Deva Prakash Devaraj, BSc, MSc<br>LV Prasad Eye Institute<br>Hyderabad, Telangana state INDIA
**Order of Authors:**	Winston Deva Prakash Devaraj, MSc
	Priya Morjaria, PhD
	Ian McCormick, MSc
	Rohit Khanna, MD
**Keywords:**	Special needs, special school eye health, Eye health awareness, Barriers to organize school eye health programs, Special needs eye health
**Abstract:**	Background<br>Children with special education needs (SEN) are at higher risk of developing vision problems; however, in India, there is inadequate data on awareness of their visual needs among special school managers (SSM). This study is aimed to identify the awareness level among SSM and barriers for organizing School Eye Health (SEH) programmes in special schools as reported by the eye health program organizers.<br>Methods<br>A mixed method study was conducted between July and August 2020 among SSM and eye health programme organizers from local eye care providers in Hyderabad, India. SSM participants completed an online questionnaire assessing their knowledge, attitude, and practice concerning the visual needs of children with SEN. Quantitative responses were described with summary statistics. Qualitative interviews with eye health programme organizers were conducted by telephone and transcripts were thematically analysed.<br>Results<br>13/67 invited SSM (19%) participated, 2/4 invited eye health programme organizers (50%) were interviewed. 92% of SSM participants were aware of vision impaired (VI) children in their schools. Awareness of potential causes of VI ranged from 54%-92% by cause, common eye conditions 8%-69% by condition and difficulties experienced by children with SEN in classroom activities 46%-77% by activity. Only 23% of special schools organized yearly SEH programmes. The eye health programme organizers reported barriers such as lack of interest from SSM, unavailability of qualified screening staff, and a lack of provision for spectacles and low vision devices.<br>Conclusion<br>This study identified varied level of knowledge, attitude, and practice about the eye health needs of children with SEN among SSM. Key barriers to conducting SEH programmes included lack of demand, human resource availability and limited access to government-funded resources. Further research is needed to undertake a comprehensive situational analysis and to plan eye health programmes for children with SEN.
**Additional Information:**	
**Question**	**Response**
**Financial Disclosure**	This study was carried out as part of MSc thesis at London School of Hygiene and

Powered by Editorial Manager® and ProduXion Manager® from Aries Systems Corporation

Enter a financial disclosure statement that describes the sources of funding for the work included in this submission and the role the funder(s) played. This includes grants and any commercial funding of the work or authors.<br><br>This statement will be typeset if the manuscript is accepted for publication.<br><br>*Please review the submission guidelines and the instructions link below for detailed requirements and guidance.*	Tropical Medicine (LSHTM), Keppel Street, London, UK. The study was supported by British Council for Prevention of Blindness, LSHTM, and Commonwealth eye health consortium.
**Competing Interests**<br><br>On behalf of all authors, disclose any competing interests that could be perceived to bias this work.<br><br>This statement will be typeset if the manuscript is accepted for publication.<br><br>*Please review the instructions link below and PLOS Global Public Health's competing interests policy to determine what information must be disclosed at submission.*	None
**Data Availability**<br><br>Before publication, Authors are required to make fully available and without restriction all data underlying their findings. Please see our PLOS Data Policy page for detailed information on this policy.<br><br>A **Data Availability Statement**, detailing where the data can be accessed, is required at first submission. Insert your Data Availability Statement in the box	The anonymized data can be made accessible if necessary in MS format

below.<br><br>Please see the data reporting section of our submission guidelines for instructions on what you need to include in your Data Availability Statement.<br><br>This statement will be typeset if the manuscript is accepted for publication.<br><br>*PLOS allows rare exemptions to address legal and ethical concerns. If you have legal or ethical restrictions, please use the box below to detail these in full sentences for the Journal team to consider.*	

Manuscript

Click here to access/download;Manuscript;Manuscript_29_05_2023 (1).docx

Eye health awareness among special school managements and barriers to organize eye health programme in special schools in Hyderabad, India

Author Information

Winston D Prakash [1,2,3]

Priya Morjaria [2]

Ian McCormick [2]

Rohit Khanna [1,4,5]

Affiliations

1. Allen Foster Community Eye Health Research Centre, Gullapalli Pratibha Rao International Centre for Advancement of Rural Eye care, L V Prasad Eye Institute, Hyderabad, Telangana, India
2. International Centre for Eye Health, London School of Hygiene and Tropical Medicine, Keppel Street, London, UK
3. Brien Holden Institute of Optometry and Vision Science, L V Prasad Eye Institute, Hyderabad, Telangana, India
4. School of Optometry and Vision Science, University of New South Wales, Sydney, Australia,
5. University of Rochester, School of Medicine and Dentistry, Rochester, NY, USA

Corresponding author and address:

6. Mr. Winston D Prakash,
7. E‐mail: winston@lvpei.org

Abstract

Background

Children with special education needs (SEN) are at higher risk of developing vision problems; however, in India, there is inadequate data on awareness of their visual needs among special school managements (SSM). This study is aimed to identify the awareness level among SSM and barriers/enablers for organizing SEH programmes in special schools as reported by the eye health program organizers.

Qualify the acronym

Methods

A mixed model qualitative study was conducted between July and August 2020 among SSM and eye health programme organizers from local eye care providers in Hyderabad, India. SSM participants completed an online questionnaire assessing their knowledge, attitude, and practice about the visual needs of Children with SEN and eye health programme organizers were interviewed by telephone. The responses were recorded and anonymised for thematic analysis.

I think it is important here to indicate that this data collection was part of the qualitative data collected

Results

low uptake

13/67 invited SSM (19%) participated, 2/4 invited eye health programme organizers (50%) were interviewed. 92% of SSM participants were aware of vision impaired (VI) children in their schools. Awareness of potential causes of VI ranged from 54%-92% by cause, common eye conditions 23%-69% by conditions and difficulties experienced by children with SEN in classroom activities 46%-77% by activity. Only 23% of SS organized yearly SEH programmes. The eye health programme organizers reported barriers such as lack of interest from SSM, unavailability of qualified screening staff, lack of provision for spectacles, and low vision devices.

Conclusion

delete

This study showed low ~~eye~~ awareness levels about eye health needs of children among SSM and there are several barriers to conduct SEH programmes which include lack of school participation, poor availability of human resource as well as treatment provisions. Further research is needed to undertake a comprehensive situational analysis and to plan eye health programs for children with SEN.

I suggest that these two sentences be separated. They are completely different

Keywords: Special needs, special school eye health, Eye health awareness, Barriers to organize school eye health programs, Special needs eye health

INTRODUCTION

Children with special educational needs (SEN) are at a higher risk of developing vision impairment (VI) and ocular comorbidities.(1, 2) In India, 28% of people living with a disability are under 19 years of age and 18% of these have a vision related disability.(3) Vision plays an important role in early childhood development such as hand-eye coordination, communication, spatial comprehension and social development.(4-6) Impaired vision can cause difficulty with daily living activities (ADL) and may lead to anxiety and depression,(7) and VI in the presence of other disabilities can amplify these difficulties further. Therefore, early identification of VI and other eye conditions in children with SEN is crucial for better health and development outcomes. (8)

School eye health (SEH) programmes have a potential to provide children with access to good quality services and can be cost-effective.(9-11) However, there is less evidence to what extent they are run in schools for children with SEN. A study by Woodhouse et al in 2014,(12) found that only 53% of special schools in Wales, UK, had conducted eye health programmes in their schools.

Hyderabad is the capital city of Telangana state in South India and has a population of over 10 million people. There are over one hundred organizations involved in teaching, training, and providing services for children with SEN. According to a local eye health organization involved in delivering community-based eye health services, most SHE (Do you mean SEH??) programmes are focused only on mainstream schools (V Sarvepally, personal email communication, 28 January 2020 – email communication available). Although studies from outside India report poor (SEH) awareness level among schoolteachers, studies from within India among mainstream schoolteachers seem to show a better awareness levels.(13-16) The aims of this study were to identify the level of eye health awareness ~~of~~ among special school managers (SSMs) and the barriers or enablers for organizing eye health programmes in special schools, from the perspective of eye health programme organizers.

METHODS

Study Design and study population

This is a mixed-methods qualitative study that included an online questionnaire and semi-structured interviews by telephone. The heads of special school management team members and programme organisers from eye care organizations involved in community outreach activities in urban and sub-urban areas of Hyderabad, India were included in the study.

Ethics

Ethics approval was obtained from the London School of Hygiene & Tropical Medicine's MSc Research Ethics Committee, London, UK and the Hyderabad Eye Research Foundation, Hyderabad, India. All participants had to read and sign an informed consent form prior to the data collection.

Inclusion and exclusion criteria

Special schools were defined as schools involved in helping children with activities such as communication, interaction, cognition, learning, catering to sensory and physical needs, helping with social and mental health, and were identified from a local tertiary eye care centre's rehabilitation center database (Figure 1). Contact details for all identified special schools in Hyderabad were obtained from the database and a publicly available online resource (www.nayi-disha.org) and schools with valid contact details were invited to participate in the study by a telephone call and an email. Headteachers, directors or principals who are directly involved in school management activities were eligible. Other organizations such as early intervention centres, therapy centres, homes for the disabled and schools for the blind were excluded from this study. Any SEH programme organizers from organizations active in the Hyderabad area with a minimum of 2 years' experience in the role were eligible.

This sounds like it should be a statement made under methodology

Data collection

The data collection was carried out between July-August 2020. Participant information and consent forms (English or Telugu language) were sent via email to each participant on invitation. After obtaining consent, a link to an online questionnaire built with Google Forms was sent to the participants. The questionnaire included demographic details and questions structured to capture knowledge, attitude, and practice with regards to the eye health need of children studying in their schools. It included multiple-choice questions and open text boxes to allow participants to express their views. Participants were asked to complete the questionnaire within two weeks from receiving but requested not to refer to any readily available resources, so their answers reflected their current understanding. Schools who did not respond to calls or emails after three attempts were considered non-responders. Those who agreed to participate and received the questionnaire but did not complete it within the two-week period were also considered non-responders.

Programme organizers were contacted by telephone to arrange an interview. An information and consent form was sent in advance. The interview guide consisted of demographic details and semi-structured questions addressing the barriers and enablers for organizing programmes in special schools. Those who did not respond to an invitation to interview were considered non-responders. None of the participants were given any financial or other incentives for participating. The individual identity of participants from both groups were concealed during data cleaning and analysis for anonymity. However, the email addresses and phone numbers of participants were available with the principal investigator as these participants were contacted through email and telephone calls.

Data analysis

Responses to the online questionnaire were downloaded and transferred into ~~a~~ an excel spread sheet (Microsoft office 2019). The descriptive statistics was carried out using Microsoft excel spread sheet 2019. Interviews were recorded and transcribed for thematic analysis. The

questionnaire responses were categorized under three different themes namely Knowledge, attitude, and practice for the analysis.

RESULTS

Questionnaire for special school management

We identified 67 eligible special schools, of which 39 could be contacted via a valid telephone number or email address. One school was public and 38 were private. All 39 schools agreed to participate, however, representatives from only 13/39 (33%) went on to complete the questionnaire. The median age of the participants was 40 years (interquartile range 35-51.5 years) and there were more females (n=9/13) than males. Eleven schools had less than 50 students while the other two had more than 100 students enrolled. Ten schools were schools for children with more than one type of disability (Table 1).

Table 1: Demographic details of school management participants

Table 1: Demographic details		
Demographics	Median(n=13)	Inter Quartile Range
Age	40	16.5 (35, 51.5)
Sex	Total number of participants	
Male	4 (31%)	
Female	9 (69%)	
Speciality of schools	Total number of schools	
Multiple disability	10 (77%)	
Autism	2 (15%)	
Intellectual disability	1 (8%)	
School strength *	Median(n=13)	Inter Quartile Range
*Total number of children in schools	36	26 (14,40)

Knowledge

Overall, 92% (n=12) respondents were aware of the number of children with eye conditions in their schools and 77% (n=10) were aware that early treatment could result in better visual outcomes. However, only 23% (n=3) had any knowledge of children's increased risk of developing eye conditions and 63% (n=9) said they identified vision problems by just observing the behaviour of a child in the classroom. 31% (n=4) felt an eye check-up should be considered only if necessary (Table 2).

Table 2: Participant response to Knowledge related questions

Table 2	
**Knowledge**	**n=13 (%)**
**Number of children with eye conditions in special schools**	
Aware	12 (92%)
Unaware	1(8%)
**Children SENs chances of developing eye conditions**	
Very likely	3 (23%)
Same as children without disability	3 (23%)
Less likely	2 (15%)
Not sure	5 (39%)
**Commonly known eye conditions**	
Squint	9 (69%)
Big eyeballs	4 (31%)
Refractive error	3 (23%)
Cataract	3 (23%)
Small eyeballs	3 (23%)
Eye cancer	2 (15%
Conjunctivitis	2 (15%)
Glaucoma	1 (8%)
**Observable symptoms in the school & classroom**	
Classroom related activities for Near	10 (77%)
Classroom related activity for distance	6 (46%)
Eye hand coordination	4 (31%)
Outdoor sports	3 (23%)
Identifying objects around	3 (23%)
Mobility	1 (8%)
Others	4 (31%)
Not sure	2 (15%)
**How often is eye check-up warranted for children SENs**	
Twice a year	5 (39%)
Once a year	4 (31%)
Only if needed	4 (31%)

Attitude

Most participants (92%; n=12) felt that children with vision problems were less capable of performing academic activities, although 69% (n=9) felt that they would be able to participate in extracurricular activities. Over half of the participants (62%; n=8) felt that children with VI or blindness, without any other additional disability, should attend normal schools as this could improve motivation and self-confidence. While 92% (n=12) said disabled children can interact with non-disabled peers, 31% (n=4) felt that they should not be attending normal school as they require special attention on a regular basis (Table 3).

Table 3: Participants response to attitude related questions

Table 3	
**Attitude**	**n = 13 (%)**
**Ability to perform academic tasks**	
Normally capable	1 (8%)
Less capable	12 (92%)
**Ability to participate in non-academic activities**	
Normally capable	9 (69%)
Less capable	1 (8%)
Not sure	3 (23%)
**Reasons for developing eye conditions in children SENs**	
Lack of Nutrition	12 (92%)
Heredity	10 (77%)
Physical illness	9 (69%)
Injury	9 (69%)
Consanguinity	7 (54%)
Curse from God	2 (15%)
**Can children with VI attend mainstream school?**	
Yes	8 (62%)
No	4 (31%)
Not sure	1 (8%)
**VI children's ability to interact with peers**	
Can interact normally	12 (92%)
Did not answer	1 (8%)

Practice

Half of the participants (46%; n=6) said that they would refer children having problems seeing in class to an eye doctor, but 23% (n=3) were unsure of what should be done. Half

(46%; n=6) had never organized any eye health programmes in their schools. 54% (n=7) participants stated that they have an additional trained caretaker staff when asked about accessible toilets and classrooms. 15% (n=2) of schools reported having ramps, railings, and support handles, while 39% (n=5) provided a nutritious diet in their schools (Table 4).

Table 4: Participants response to practice related questions

Table 4	
**Practice**	**n=13(%)**
**If a child faces difficulty in reading & writing**	
Refer to eye doctor	6 (46%)
Modify working distance	3 (23%)
Help	1 (8%)
Did not answer	1 (8%)
Others	1 (8%)
**What classroom adaptations are made for VI children?**	
Approach magnification	7 (54%)
Encourage them to use and provide assistive aids	5 (39%)
Give extra time	2 (15%)
Environmental modifications	1 (8%)
Others	7 (54%)
Did not answer	2 (15%)
**If a child is found to have some eye problem**	
Refer to eye doctor	7 (54%)
Provide special care	1 (8%)
Help them wear glasses	1 (8%)
Ask them to stay home until they get better	2 (15%)
Did not answer	2 (15%)
**If a child has an untreatable eye condition**	
Create adaptive environments	4 (31%)
Refer them to doctor/rehabilitation	3 (23%)
Not sure	3 (23%)
Others	3 (23%)
**How often eye health programmes are organised in schools?**	
Once a year	3 (23%)
Twice a year	1 (8%)
Rarely	2 (15%)
Never	6 (46%)
**Available facilities to access toilets and classrooms**	
Extra trained caretaker staff	7 (54%)
Railings, ramps, and support handles	2 (15%)
Comfortable seating	1 (8%)
None	2 (15%)
Did not answer	1 (8%)
**Provision for nutritious diet for children in their schools**	
Educate the parents	6 (46%)
Provide nutritious diet at school	5 (39%)
None	2 (15%)

Did not answer	1 (8%)

Interview with eye health programme organizers

Of four known community outreach organizers from four different organizations in Hyderabad, two responded and agreed to be interviewed. One was a public health specialist with more than 2 decades of experience and the other, an optometrist and head of community ophthalmology services with a decade long experience in the field.

Interview responses

Interviewee responses on the status of SEH programmes in special schools are summarized in Box 1. Overall, the frequency of SEH programmes in special schools seem to be very low. Eye examination included as a part of annual health check-up in these schools is being one of the main reasons reported for the less frequent SEH programmes in special schools. Furthermore, there are no initiatives, neither from the organizing team or from the special schools for conducting SEH programmes in special schools.

**Box 1: Frequency of SEH programmes conducted in special schools**	
Participant 1	<1% out of 50 schools covered in a year were special schools
Participant 2	<2% out of 1000 schools covered in a year were special schools
**<u>Reasons for less coverage</u>**	
Participant 1	Eye health assessment as part of annual check-up is already happening
Participant 2	As eye check-up at the time of admission has already been done there are no further eye health services sought by the school or the parents
**<u>Proactiveness in organizing SEH programmes</u>**	
Participant 1	Organize only if requested by the schools/governmental agencies
Participant 2	Organize as part of initiatives from governmental agencies or NGO's
	Special schools seldom take initiative in organizing SEH programmes

Barriers to organizing SEH programmes in special schools are summarized in Box 2. The main barriers included are lack of provision and unaffordability of spectacles and low vision

devices for children in special schools. One organizer said" The governmental provisions for availing spectacles and low vision devices (LVDs) for free of charge remain only on the policy level and never gets to see the light of the day". Another organizer said, "Future sustainability of SEH programme in special schools will be a major challenge due to lack of enthusiasm from the stakeholders including school managements, parents as well as programme organizers."

**Box 2: Barriers for organizing SEH programmes in special schools reported by the organizers**
• Unwillingness of teachers to participate in the screening process as volunteers or for helping the screening team
• Unavailability of skilled manpower (optometrists/ophthalmologists) for special school programmes
• Increased economic cost pertaining to skilled manpower and equipment needed
• Unwillingness/unavailability of parents to participate in programmes
• Lack of provision and unaffordability of spectacles, and LVDs and inaccessibility of governmental aids for purchasing these devices
• School infrastructures are inaccessible
• Currently there are no standard screening protocols available for special school eye health programmes

Existing good practices among the schools and eye health providing teams and points to be considered in the future programmes are summarized in box 3. Medical record keeping among the schools and creating awareness among parents and teachers during the screening process, team member training in handling children with SEN are some of the good practices that are existing among the stakeholders and the screening teams. One organizer stated that "There are no standard protocols for special school eye heath programs, hence this needs to be developed".

**Box 3: List of existing good practices and points to be considered in the future programmes**
**<u>From Schools side</u>**
• Medical history record keeping for every child – Helps in understanding the overall medical conditions of children
**<u>From eye care providers side</u>**

• Helping the parents understand the eye health needs of their child during eye examination
• Creating awareness among the schoolteachers about the need for eye care services for children with SEN
• Mandatory training of screening team members prior to the programme in examining children with SEN
**Points to be considered prior to organizing SEH programmes in special schools**
• Making sure that the programme venues are accessible
• Inform parents/guardians well in advance and suggest them to be available during the screening
• Develop a standard operating protocol pertaining to special school eye health programmes
• Stakeholder involvement should include local NGOs and volunteers
• Making sure that the provision for spectacles and LVDs are available
• Governments should consider monetary or non-monetary incentives for participating NGOs/organizations

DISCUSSION

Universal eye health and the Sustainable Development Goal 3 “to ensure healthy lives for all people at all ages” cannot be achieved without ensuring equitable access to eye health care for underserved groups. This study surveyed the knowledge, attitudes, and practices of heads of schools for children with SEN and interviewed school eye health programme organizers in Hyderabad to understand whether there was a potential service gap for this group of at-risk children.

Encouragingly, almost all participants said they were aware of the number of children in their schools with eye conditions and that early intervention might result in better eye health outcomes. Despite this, only half of the participants said they had organized an eye health programme and only half of them said that they would refer a child having vision problems to an eye doctor. Even though most participants knew that children with SEN needed an eye check-up at least once or twice a year, this happened in less than a third of schools, lower than the 53% reported in a study from Wales, UK in 2014.(12)

Refractive error being one of the most common conditions found among children with SEN,(17, 18) only 23% of participants were aware of this condition. Almost all participants said poor nutrition could cause eye problems, but none of the participants mentioned about

vitamin A deficiency (VAD). Although there are not many studies reporting VAD in children with SEN, one study reported low vitamin A levels among Autism spectral disorders from China in 2021.(19) This may also indicate that many of these participants were unaware of the importance of vitamin A for good eye health, which may also directly impact the nutrition that is provided in the schools.

Most participants exhibited a negative attitude towards vision-impaired children’s ability to perform academic tasks. This could negatively impact eye health seeking behaviour or referral practices among school staff. Almost two thirds of the participants thought children with only vision impairment could study in mainstream schools assuming that these schools are inclusive. They also felt studying with normal peers could improve their psychosocial well-being although this claim is supported by several studies carried out in developed countries (20), the evidence in LMICs is inadequate.(21)

Apart from creating adaptive environments in schools, many participants said they provide assistive devices; however, one cannot justify the reliability and effectiveness of these practices which require certain level of professional training and competency. In addition, they also did not mention about any specific devices pertaining to aid the visually impaired. Hence, it is possible that this response could be due to a mere assertion of broader terminology used with regards to any disability and cannot justify the awareness level pertaining to aiding vision related difficulty. The low level of knowledge about the eye health needs of children with SEN among special school heads in Hyderabad is comparable to reports from mainstream primary schools outside India,(14, 15, 22) however, it does not correlate with mainstream primary schools in India.(13, 16) This could be due to higher coverage of mainstream schools during SEH programmes in India compared to special schools, which may also increase the awareness level among mainstream schoolteachers.

Interviews with SEH programme organizers point to various barriers for implementing programmes in special schools. Limited resources were of a major concern: assessing children with SEN requires qualified eye care workers such as optometrists or

ophthalmologists, with expertise in paediatric and low vision assessment (LVA). Equipment used for LVA and LVDs were noted to be expensive to procure. Furthermore, LVA will be a challenging task on the screening sites due to the various logistical constraints, in addition to being time consuming. Hence, it poses a need for appropriate referral set ups in the proximity for the referred children to avail these services. Appropriate stakeholder engagement in schools and parents was another major concern. Lack of motivation among teachers was reported on the assumption that they are already overburdened with other health programmes and responsibilities. The organizers reported that there is less scope for the screening teams to conduct eye health awareness sessions for the primary stakeholders (parents/guardians), as most of them do not attend the screening programmes. This may also have a significant impact on the continuum of care for children with eye health needs and may further lead to poor referral uptake.

The interviewees made several recommendations to improve knowledge, attitude, practice of stakeholders and to increase uptake of eye health programmes in special schools. They suggested incorporating health promotion strategies within SEH programmes could make them more impactful and might indirectly address the issue of sustainability. The more informed the stakeholders are about the eye health needs of these children, the more they might proactively take them for regular eye examinations, schools may include eye examination as part of their annual health programmes and teachers may become more aware and sensitive towards the eye health needs of these children. It was also reported that financial protection against the cost of spectacles and LVDs could reduce the burden on families who may already face increased health care costs associated with caring for children with SEN.(23)

Limitations

This study should be interpreted in the context of its limitations. Firstly, the participants recruited were the school heads from a minority of special schools in the Hyderabad area and the participants of these institutions may not be representative of all

headteachers/schools in the area. Secondly, the study period was interrupted by COVID-19 travel restrictions, meaning face-to-face sensitization and enrolment of headteachers was not possible. Thirdly, although the questionnaire was piloted among the peers of the principal investigator, it was not validated for the target audience, which might have resulted in varying levels of understanding between participants. Finally, the day-to-day engagement of the headteachers with the children in their schools was unverifiable and it might have been valuable to have recruited class teachers for more insight into the daily activities and challenges faced by the children, however, this was hampered by the pandemic which posed difficulty in contacting the class teachers.

Conclusion

Despite the limitations, this study highlights a potentially important service gap for SEH programmes in India. Measuring the eye health awareness level among schoolteachers in a larger sample could give a better insight into the matter. In addition, it proposes a need to include eye health promotion and training of teachers in identifying symptoms of vision impairment to be an integral part of SEH programs in special schools. Further situational analysis should include a survey of prevalence of VI and other eye conditions among children with SEN to understand the current gap and need.

Acknowledgements

The authors would like to acknowledge the help received from Ms.Mahalaxmi Mojjada for translating the consent form and questionnaire into Telugu language. The authors also would like to thank all the participants for their valuable time for responding to the questionnaire and the interview.

Financial support

This study was carried out as part of MSc thesis at London School of Hygiene and Tropical Medicine (LSHTM), Keppel Street, London, UK. The study was supported by British Council for Prevention of Blindness, LSHTM, and Commonwealth eye health consortium.

Conflicts of interest

There are no conflicts of interests.

References

1. Das M, Spowart K, Crossley S, Dutton GN. Evidence that children with special needs all require visual assessment. Arch Dis Child. 2010;95(11):888-92.

2. Salt A, Sargent J. Common visual problems in children with disability. Arch Dis Child. 2014;99(12):1163-8.

3. Disabled Persons in India a statistical profile 2016 (Cited 2020 April 30) [Available from: http://mospi.nic.in/sites/default/files/publication_reports/Disabled_persons_in_India_2016.pdf.

4. Andersen ES, Dunlea A, Kekelis LS. Blind children's language: resolving some differences. J Child Lang. 1984;11(3):645-64.

5. Hatton DD, Bailey DB, Burchinal MR, Ferrell KA. Developmental Growth Curves of Preschool Children with Vision Impairments. Child Development. 1997;68(5):788-806.

6. Molloy A, Rowe FJ. Manneristic Behaviors of Visually Impaired Children. Strabismus. 2011;19(3):77-84.

7. Kempen GI, Ballemans J, Ranchor AV, van Rens GH, Zijlstra GA. The impact of low vision on activities of daily living, symptoms of depression, feelings of anxiety and social support in community-living older adults seeking vision rehabilitation services. Qual Life Res. 2012;21(8):1405-11.

8. Kulkarni SK, Veer AB, Gaikwad A, Deshpande M. The impact of early intervention on a child with vision loss in infancy - A parental perspective. Indian journal of ophthalmology. 2022;70(9):3377-81.

9. Frick KD, Riva-Clement L, Shankar MB. Screening for refractive error and fitting with spectacles in rural and urban India: cost-effectiveness. Ophthalmic Epidemiol. 2009;16(6):378-87.

10. Lester BA. Comparing the cost-effectiveness of school eye screening versus a primary eye care model to provide refractive error services for children in India. Community Eye Health. 2007;20(61):15.

11. Saravanan S, Krishnamurthy S, Ramani KK, Narayanan A. Cost Analysis of a Comprehensive School Eye Screening Model from India. Ophthalmic epidemiology. 2022:1-7.

12. Woodhouse JM, Davies N, McAvinchey A, Ryan B. Ocular and visual status among children in special schools in Wales: the burden of unrecognised visual impairment. Arch Dis Child. 2014;99(6):500-4.

13. Ambika K, Nair N. A Study on Awareness of Primary School Teachers Regarding Refractive Errors and its Early Identification among Primary School Children. International Journal of Nursing Education. 2013;5:6-9.

14. Habiba U, Ormsby GM, Butt ZA, Afghani T, Asif M. Knowledge and practices of teachers associated with eye health of primary school children in Rawalpindi, Pakistan. Taiwan J Ophthalmol. 2017;7(1):28-33.

15. Maru A, Belete G, Adimassu N. Knowledge, attitude and associated factors among primary school teachers regarding refractive error in school children in Gondar city, Northwest Ethiopia. PLOS ONE. 2018;13:e0191199.

16. Thomas A. A comparative study to assess the knowledge of primary school teachers regarding refractive errors among primary school children in selected urban and rural schools, Mangalore: RajivGandhi University of Health Science. 2013.

17. Gogate P, Soneji FR, Kharat J, Dulera H, Deshpande M, Gilbert C. Ocular disorders in children with learning disabilities in special education schools of Pune, India. Indian J Ophthalmol. 2011;59(3):223-8.

18. Pehere NK, Khanna RC, Marlapati R, Sannapaneni K. Prevalence of ophthalmic disorders among hearing-impaired school children in Guntur district of Andhra Pradesh. Indian Journal of Ophthalmology. 2019;67(4):530-5.

19. Wen J, Yang T, Zhu J, Guo M, Lai X, Tang T, et al. Vitamin a deficiency and sleep disturbances related to autism symptoms in children with autism spectrum disorder: a cross-sectional study. BMC pediatrics. 2021;21(1):299.

20. Price R. Inclusive and special education approaches in Developing countries: (Cited 2020 August 14) Available from: https://assets.publishing.service.gov.uk/media/5c6ac403ed915d4a39787401/373_Inclusive_and_Special_Education_Approaches.pdf. 2018.

21. Kuper, H, Saran, A & White, H 2018, Rapid evidence assessment (REA) of what works to improve educational outcomes for people with disabilities in low- and middle-income countries, Campbell Collaboration, and International Centre for Evidence in Disability, [Oslo], (cited 29 May 2023), Available from: https://www.gov.uk/government/publications/improving-educational-outcomes-for-people-with-disabilities-in-low-and-middle-income-countries-why-does-it-matter-and-what-works.

22. Okoloagu N, Okoye O, Onwubiko S, Eze C, Eze B, Chuka-Okosa C. A Survey of Teachers; Knowledge, Attitudes, and Practices Related to Pupils’ Eye Health and School-Based Eye-Health Services. Nigerian Journal of Ophthalmology. 2019;27(2):68-75.

23. Malvankar-Mehta MS, Wilson R, Leci E, Hatch K, Sharan S. Cost and quality of life of overlooked eye care needs of children. Risk management and healthcare policy. 2018;11:25-33.

A cross-sectional survey of eye health knowledge, attitude, and practice among special school managers and barriers to eye health programmes in special schools in Hyderabad, India

Author Information

Winston D Prakash [1,2,3]

Priya Morjaria [2]

Ian McCormick [2]

Rohit Khanna [1,4,5]

Affiliations

1. Allen Foster Community Eye Health Research Centre, Gullapalli Pratibha Rao International Centre for Advancement of Rural Eye care, L V Prasad Eye Institute, Hyderabad, Telangana, India
2. International Centre for Eye Health, London School of Hygiene and Tropical Medicine, Keppel Street, London, UK
3. Brien Holden Institute of Optometry and Vision Science, L V Prasad Eye Institute, Hyderabad, Telangana, India
4. School of Optometry and Vision Science, University of New South Wales, Sydney, Australia,
5. University of Rochester, School of Medicine and Dentistry, Rochester, NY, USA

Corresponding author and address:

6. Mr. Winston D Prakash,
7. E- mail: winston@lvpei.org

ABSTRACT

Background

Children with special education needs (SEN) are at higher risk of developing vision problems; however, in India, there is inadequate data on awareness of their visual needs among special school managers (SSM). This study is aimed to identify the awareness level among SSM and barriers for organizing School Eye Health (SEH) programmes in special schools as reported by the eye health program organizers.

Methods

A mixed method study was conducted between July and August 2020 among SSM and eye health programme organizers from local eye care providers in Hyderabad, India. SSM participants completed an online questionnaire assessing their knowledge, attitude, and practice concerning the visual needs of children with SEN. Quantitative responses were described with summary statistics. Qualitative interviews with eye health programme organizers were conducted by telephone and transcripts were thematically analysed.

Results

13/67 invited SSM (19%) participated, 2/4 invited eye health programme organizers (50%) were interviewed. 92% of SSM participants were aware of vision impaired (VI) children in their schools. Awareness of potential causes of VI ranged from 54%-92% by cause, common eye conditions 8%-69% by condition and difficulties experienced by children with SEN in classroom activities 46%-77% by activity. Only 23% of special schools organized yearly SEH programmes. The eye health programme organizers reported barriers such as lack of interest from SSM, unavailability of qualified screening staff, and a lack of provision for spectacles and low vision devices.

Conclusion

This study identified varied level of knowledge, attitude, and practice about the eye health needs of children with SEN among SSM. Key barriers to conducting SEH programmes included lack of demand, human resource availability and limited access to government-funded resources. Further research is needed to undertake a comprehensive situational analysis and to plan eye health programmes for children with SEN.

Keywords: Disability, eye health, child health, school health, special school eye health

INTRODUCTION

Children with special educational needs (SEN) are at a higher risk of developing vision impairment (VI) and ocular comorbidities.(1, 2) In India, 28% of people living with a disability are under 19 years of age and 18% of these have vision related disability.(3) Vision plays an important role in early childhood development such as hand-eye coordination, communication, spatial comprehension and social development.(4-6) Impaired vision can lead to difficulty in performing activities of daily living (ADL), anxiety and depression,(7), additionally, VI in the presence of other disabilities can amplify these difficulties further. Therefore, early identification of VI and other eye conditions in children with SEN is crucial for better health and development outcomes. (8)

Special schools help children with activities such as communication, interaction, cognition, learning, catering to sensory and physical needs, helping with social and mental health.(9) School eye health (SEH) programmes have a potential to provide children with access to high quality eye care services and can be cost-effective.(10-12) However, there is less evidence to what extent they are run in schools for children with SEN. A study by Woodhouse et al in 2014,(13) found that only 53% of special schools in Wales, UK, had conducted eye health programmes in their schools. A study in 2019 by Donaldson LA et al,(14) argued that school entry vision screening is ineffective for children in special schools in England, mainly due to low uptake of primary care service and limited cooperation levels. However, such programs might be the only option to reach these children in many low-income settings, owing to the poor parental awareness or availability of basic services such as vision screening and referral services. A recent study by Allen LC et al in 2021, reporting on availability of eye care services for children with SEN found, 31% service providers did not have provision for these children in their practice.(15) We found no evidence on the proportion of special schools in India that have held eye health programmes.

Hyderabad is the capital city of Telangana state in South India and has a population of over 10 million people. There are over one hundred organizations involved in teaching, training,

and providing services for children with SEN. According to a local eye health organization involved in delivering community-based eye health services, most SEH programmes are focused only on mainstream schools (V Sarvepally, personal email communication, 28 January 2020 – email communication available). Among studies on the knowledge, attitude, and practice of mainstream schoolteachers about the eye health needs of children, studies in India showed higher levels compared to those in other countries.(16-19) However, there are no such studies reporting on the level of awareness about the eye health needs of children with SEN and/or knowledge, attitude, and practice (KAP) among the special school managers (SSM). There is also evidence that increased eye health awareness levels among the school staff, children and parents could result in increased service uptake.(20, 21) Therefore, the aims of this study were firstly to identify the level of eye health awareness of among special school managers and secondly, to identify barriers to organizing eye health programmes in special schools, from the perspective of eye health programme organizers.

METHODS

Ethics

Ethics approval was obtained from the London School of Hygiene & Tropical Medicine's MSc Research Ethics Committee, London, UK and the Hyderabad Eye Research Foundation, Hyderabad, India. Participation was voluntary and none of the participants were given any financial or other incentives for participating. All participants read and signed an informed consent form in English or Telugu before enrolling. Participants were advised that all responses would be anonymised for reporting.

Study design, participants, and recruitment

This was a mixed methods study consisting of a quantitative and qualitative component: an online questionnaire to assess KAP among SSM, and semi-structured interviews by telephone to understand the barriers to organizing SEH programmes in special schools. For the online questionnaire, participants were representatives of special schools identified from

a local tertiary eye care centre's rehabilitation centre database (Figure 1). Contact details for all identified special schools in Hyderabad were obtained from the database and a publicly available free online resource (www.nayi-disha.org). Schools with valid contact details were invited to participate in the study by a telephone call and an email.

For the qualitative interviews, participants were programme organisers from eye care organizations involved in community outreach activities in urban and sub-urban areas of Hyderabad, India. Programme organizers were invited to participate by email and those who responded were included in the study.

Inclusion and exclusion criteria

Any special school in our area of interest with valid contact details was eligible for inclusion. Organizations such as early intervention centres, therapy centres, homes for the disabled and schools for the blind were excluded. Within schools, only headteachers, directors or principals who were directly involved in school management activities (referred to in this study as 'managers') were eligible for inclusion. Any organization that was active in conducting community eye health activities in the Hyderabad region was eligible for inclusion. Within these organizations, any SEH programme organizer with a minimum of two years' experience was eligible for inclusion.

Data collection

Data collection was carried out between July-August 2020. After giving consent, participants were emailed a link to an online questionnaire built with Google Forms. The questionnaire included some questions used in previous studies conducted in mainstream schools,(16-19, 22) plus additional questions written for this study. The questionnaire included demographic details and questions structured to capture KAP with regards to the eye health needs of children attending special schools. It included multiple-choice questions and open-ended text boxes to allow participants to express their views (Supplementary material). Participants were asked to complete the questionnaire within two weeks and not to refer to the internet,

so their answers reflected their current understanding. Schools who did not respond to calls or emails after three attempts were considered non-responders. Those who agreed to participate and received the questionnaire but did not complete it within the two-week period were also considered non-responders.

Programme organizers were contacted by telephone to arrange an interview. An information and consent form were sent in advance. The interview guide consisted of demographic details and semi-structured questions addressing the barriers to organizing programmes in special schools. Those who did not respond to an invitation to interview were considered non-responders.

Data analysis

Responses to the online questionnaire were downloaded and transferred to a Excel spreadsheet (Microsoft, 2019). Summary statistics for participant demographics and MCQ responses were calculated in Excel. Any responses in open text boxes were listed as common subthemes such as good and poor knowledge, positive and negative attitudes, acceptable and non-acceptable practices under the overall themes of knowledge, attitude, and practice. Interviews were audio recorded and transcribed for thematic analysis. For the thematic analysis, each response was familiarized, and codes were created for generating themes (as described by Braun and Clark).(23) These themes were further categorized under common category of themes for the interpretation as barriers and suggestions.(23)

RESULTS

Questionnaire responses of special school managers

We identified 67 special schools, among whom, only 39 had a valid contact detail and could be contacted. One was public and 38 were private schools. Although all 39 schools agreed to participate, only 13/39 (33%) went on to complete the questionnaire within the time allowed. The median age of the participants was 40 years (interquartile range 35-51.5 years) and there was a female preponderance at 69% (n=9/13). Eleven schools had less than 50

students while the other two had more than 100 students enrolled. Ten schools were schools for children with more than one type of disability (Table 1).

Table 1: Demographic details of school management participants

Demographics	**Median (n=13)**	**Inter Quartile Range**
Age	40	16.5 (35, 51.5)
Sex	Total number of participants n=13 (%)	
Male	4 (31)	
Female	9 (69)	
Speciality of schools	Total number of schools n=13 (%)	
Multiple disability	10 (77)	
Autism	2 (15)	
Intellectual disability	1 (8)	
	Median (n=13)	Inter Quartile Range
Number of children in schools	36	26 (14,40)

Knowledge

Overall, 92% (n=12) of respondents were aware of the number of children with eye conditions in their schools and 77% (n=10) were aware that early treatment could result in better visual outcomes. However, only 23% (n=3) had any knowledge that children with SEN have increased risk of developing eye conditions. Sixty three percent (n=9) said they identified vision problems by just observing the behaviour of the child in classrooms. The main emergent themes in the open-ended text box responses with regards to symptoms of reduced vision faced by the children in the classroom was *"Difficulties faced in performing academic tasks"* which included difficulty in copying from blackboard, difficulty in writing and reading at near distance which was reported by 53.8% (n=7) of participants, followed by *"Poor hand eye coordination while performing near activities"* which was reported by 30.8% (n=4). Another major theme that emerged was *"Difficulty in playing or participating in sports"* which was reported by 30.8% (n=4). Most participants said lack of nutrition (92.3%, n=12) and hereditary predisposition (76.9%, n=10) were the main reasons for children developing eye problems and 31% (n=4) felt an eye check-up should be considered only if necessary. The most well-known condition was squint (69.23%, n=9) and the least well-known was glaucoma (7.69%, n=1) (Table 2).

Table 2: Participant response to Knowledge related questions

Table 2	
**Participant responses for knowledge related questions**	
**Knowledge**	**n=13 (%)**
**Are you aware of the number of children with eye conditions in your school? (MCQ type)**	
Yes	12 (92%)
Not sure	1(8%)
**How likely are children with SEN to develop eye problems? (MCQ type)**	
Very likely	3 (23%)
Same as children without disability	3 (23%)
Less likely	2 (15%)
Not sure	5 (39%)
**Among the following eye conditions, which are the most common conditions that could affect children with SEN? (MCQ type)**	
Squint	9 (69%)
Big eyeballs	4 (31%)
Refractive error	3 (23%)
Cataract	3 (23%)
Small eyeballs	3 (23%)
Eye cancer	2 (15%
Conjunctivitis	2 (15%)
Glaucoma	1 (8%)
**What do you think is/are the reason/s for children with SEN developing eye problems (MCQ type)**	
Heredity	10 (76.9)
lack of Nutrition	12 (92.3)
Physical illness	9 (69.23)
Injury	9 (69.23)
Consanguinity	7 (53.85)
Curse from God	2 (15.4)
**What are the symptoms that could indicate a child of having vision problem? (Open ended)**	
Difficulty in reading and writing	7 (53.8)
Difficulty in sports activities	4 (30.8)
Difficulty in mobility	1 (7.7)
Difficulty in eye hand coordination	4 (30.8)
Difficulty in copying from blackboard	7 (53.8)
Difficulty in identifying objects around	3 (23.1)

Others	4 (30.8)
Not sure	3 (23.1)
**How often is an eye check-up warranted for children with SEN? (MCQ type)**	
Twice a year	5 (39%)
Once a year	4 (31%)
Only if needed	4 (31%)

Attitude

Most participants (92%; n=12) felt that children with vision problems were less capable of performing academic activities, although 69% (n=9) felt that they would be able to participate in extracurricular activities. Over half of the participants (62%; n=8) felt that children with VI or blindness, without any other additional disability, should attend normal schools as this could improve motivation and self-confidence. While 92% (n=12) said disabled children can interact with non-disabled peers, 31% (n=4) felt that they should not be attending normal school as they require special attention on a regular basis and need for additional teaching materials and methods.

To the question asked in open text boxes about why/why not children with VI should be attending mainstream schools, one major theme that emerged was *"Inclusive schools."* A third of participants (30.8%; n=4) felt that children with VI should attend regular schools if they are inclusive, while 15% (n=2) said they should attend normal school if these schools provided Braille training. A participant stated, "*The teachers need to pay more attention while teaching these children, which is not practically possible in regular schools*," while another participant said, "*They should go to an inclusive school, they deserve to be with normal children*" (Table 3).

Table 3: Participants response to attitude related questions

**Table 3**	
**Participant responses for attitude related questions**	
**Attitude**	**n = 13 (%)**

**Do you think children with SEN can perform academic tasks? (Open ended)**	
Yes, they can perform academic tasks	1 (8%)
No, they cannot perform academic tasks	12 (92%)
**Do you think children with SEN are capable to participate in non-academic activities such as sports or extracurricular activities? (Open ended)**	
Yes, they can participate and perform	9 (69%)
Yes, but not fully capable	1 (8%)
Not sure	3 (23%)
**Can children with vision impairment attend mainstream school? (Open ended)**	
Yes	8 (62%)
No	4 (31%)
Not sure	1 (8%)
**Do you think children with vision impairment can interact with normal peers? (Open ended)**	
Can interact normally	12 (92%)
Did not answer	1 (8%)

Practice

Close to half of the participants (46%; n=6) said that they would refer children who face difficulty in academic tasks such as reading and writing to an eye doctor, but 46% (n=6) were unsure of what to do or gave generic responses, for e.g. "*We never faced such issues in our school" or "I would counsel the parents*". However, 54% (n=7) participants said they make the children sit closer to the blackboard or the instructor, if they had difficulty in copying from the blackboard. One participant said," *I would encourage the child to use his/her spectacles*" while another said, "*We allow the child to use prescribed low vision device when needed."* Forty six percent (n=6) of participants said they had never organized an eye health programme in their schools in the past, while one participant said, "*We approached few doctors for eye camps, but they asked us to bring the children to their hospital"*. When asked about availability of accessible facilities in their schools, 54% (n=7) participants stated that they have additional trained caretaker staff, while one participant stated, *"We do not have any child with low vision in our school"*. Thirty nine percent (n=5) of

the schools provided a nutritious diet in their schools. Twenty three percent (n=3) participants stated that they provided a mixer of nutritious diet including pulses, millet, finger millet, fruits, green leafy vegetables as salads, chicken meat, boiled eggs, and milk in their schools while few stated that they did not provide any food in their schools. However, two participants stated that they check the lunch box and give feedback/suggestions to the parents to include nutritious diet (Table 3).

Table 4: Participants response to practice related questions

**Table 4**	
**Participant responses for practice related questions**	
**Practice**	**N=13 (%)**
**What would you do if you see a child keeping books close to his/her face while reading and writing? (Open ended)**	
Refer to eye doctor	6 (46%)
Modify working distance	3 (23%)
Help in copying and writing	1 (8%)
Did not answer	1 (8%)
Others	1 (8%)
**What classroom adaptations are made for vision impaired children in your school? (Open ended)**	
Encourage them to use prescribed optical devices	2 (15.4)
Ask the children to sit closer to the blackboard or the teacher	7 (53.85)
Provide large font and bright coloured materials	3 (23.1)
Braille training	2 (15.4)
Provide extra time to complete the task	2 (15.4)
Did not answer	2 (15.4)
**What would you do If you suspect a child to have eye problem? (Open ended)**	
Refer to eye doctor	7 (54%)
Provide special care	1 (8%)
Help them wear glasses	1 (8%)
Ask them to stay home until they get better	2 (15%)
Did not answer	2 (15%)
**What would you do If a child has an untreatable eye condition? (Open ended)**	
Create adaptive environments	4 (31%)
Refer them to doctor/rehabilitation	4 (31%)
Not sure	3 (23%)

Incoherent or generic responses	3 (23%)
**How often eye health programs are organised in schools? (MCQ)**	
Once a year	3 (23%)
Twice a year	1 (8%)
Rarely	2 (15%)
Never	6 (46%)
**What are the facilities available for children in your schools with regards to accessibility? (Open ended)**	
Extra trained caretaker staff	7 (54%)
Railings, ramps, and support handles	2 (15%)
Comfortable seating	1 (8%)
None	2 (15%)
Did not answer	1 (8%)
**Do you provide nutritious diet for children in your school? (Open ended)**	
No such provisions at the school	6 (46%)
Suggest parents to include nutritious diet	2 (15%)
Provide nutritious diet at school	5 (39%)
None	2 (15%)
Did not answer	1 (8%)

Interview responses of eye health programme organizers

Of four known community outreach organizers from four different organizations in Hyderabad, two responded and agreed to be interviewed. One was a public health specialist with more than 2 decades of experience and the other, an optometrist and head of community ophthalmology services with a decade of experience in the field. Interview transcripts were coded for thematic analysis. The three major themes were a) barriers to organizing SEH programs, b) existing good practice, and c) suggestions to improve services. Under each theme, sub-themes were coded and categorized.

Barriers

Low programme coverage

Most of the special schools covered by their respective organizations were run by the government and were mostly located in urban areas. One organizer covered 1000 schools every year of which only 0.5% were special schools, the second organizer covered 50 schools in a year of which less than 2% were special schools. The reasons for the low coverage/exclusion of special schools in their programmes are describe as follows: Firstly, both interviewees said that the reason for the exclusion of special schools in their programmes was that they assumed but not sure that an eye examination was carried out as part of an annual general health check-up conducted in special schools. The second reason for the low coverage of special schools was that the time taken for completing a SEH programme in one special school was much longer compared to the time taken for one mainstream school which is even more challenging due to the limited time availability to complete their programmes within the school calendar due to frequent public holidays, school vacations and exam periods. Hence special schools are not prioritized.

Programme Initiative or lack of ownership

Special SEH programmes are usually done as stand-alone programmes and hence they are rarely included in government or NGO sponsored activities. Government sponsored programmes are not usually inclusive of special schools and most programmes run by both governmental and NGOs are focused on the number of children screened rather than needs-based activity. Hence, the initiative from these organizations is limited. On the other hand, special schools seldom take the initiative in organizing eye health programmes. Overall, there is a lack of motivation from all the stakeholders concerned. One interviewee said, "*Very rarely we receive requests from one or two private schools to conduct eye health programmes in their schools, but never from special schools*."

A lack of stakeholder engagement

Special schoolteachers are already occupied with various responsibilities pertaining to other governmental/non-governmental activities. Therefore, they are unwilling or lack motivation to

take up additional responsibilities. Without schoolteachers' involvement it is impossible to examine children with SEN, which is another reason for the organizers not considering special schools in their programmes. In some situations, the teachers or the staff are often transferred to a different school or unavailable, which makes it difficult to convince the new management or teachers to help in organizing programmes in these schools. Unlike mainstream schools, special SEH programmes require the parents to attend while the eye examination is being carried out. However, it is difficult to coordinate parents' availability for all the children on the day of the programme. Hence, there is limited opportunity to educate and create awareness among the parents about their children's eye condition and needs.

Skilled human resource shortages

Unlike mainstream schools, special schools require skilled workforce who are trained in the assessment of children with SEN, low vision assessment (LVA), prescription of spectacles and low vision devices (LVDs). Most SEH programmes are conducted by community eye health workers, schoolteachers and vision technicians who are not trained to carry out a complete eye examination. Involving a more skilled workforce, i.e., optometrists or ophthalmologists, would incur additional costs. In some circumstances, such cadres are unwilling to participate in the SEH programmes due to long-distance travel and limited financial benefits. Therefore, most special SEH programmes are conducted by optometry trainees unsupervised.

Impossible to task-shift to non-specialists

Training of schoolteachers, community eye health workers (CEHW) and administrative staff in screening of children with SEN is not possible as it requires more time and expertise in the field. One interviewee said, *"I can understand the difficulties in assessing these children owing to the training that I received during my low vision assessment training, which was part of my undergraduate degree, but not everyone can understand. So, I think having a basic training in assessing these children helps in organizing such programmes."* Children

with SEN require additional tests for vision assessment and refraction which cannot be performed by school staff and CEHW. Additionally, there is a limited scope for training children in usage of spectacles or LVDs due to the unavailability of parents, time, and training environment.

Additional clinical equipment and assessment needs

A complete eye examination for children with SEN requires expensive equipment, hence increased demand for financial resources. Secondly, only dry refraction can be performed in the school premises and children require a referral for cycloplegic refraction. Poor referral uptake among this group makes it difficult to provide effective refractive error correction via SEH programmes alone.

Poor availability and affordability of spectacles and low vision devices

Interviewees said that the low-cost spectacles which are commonly provided through SEH programmes are not of good quality, hence there is a higher chance of breakage and discomfort to children. However, providing good quality spectacles requires increased financial resources which is a major challenge. Additionally, unavailability and unaffordability of LVDs pose a major challenge. Most of the time these devices are just prescribed and not provided. It is highly likely that the parents do not purchase them. One organizer said, *"Although governmental provisions are available for purchasing LVDs, these are available only on paper and not accessible in reality."*

Good practices and suggestions

The two themes of existing good practice in special schools and organizations and suggestions for improved service provision are summarized in Table 4.

**Table 4: List of existing good practices and points to be considered in the future programmes**
**<u>Good practices followed in special schools</u>**
• Medical history record keeping for every child – Helps in understanding the overall medical conditions of children
**<u>Good practices followed by eye care providers</u>**
• Helping the parents understand the eye health needs of their children during eye examination
• Creating awareness among the schoolteachers about the need for eye care services for children with SEN
• Mandatory training of screening team members prior to the programme in examining children with SEN
**<u>Suggestions: Points to be considered prior to organizing SEH programmes in special schools</u>**
• Making sure that the programme venues are accessible
• Inform parents/guardians well in advance and encourage them to be available during the screening
• Develop a standard operating protocol pertaining to special school eye health programmes
• Stakeholder involvement should include local NGOs and volunteers
• Facilitate the availability of good quality spectacles and LVDs
• Government should consider monetary or non-monetary incentives for participating NGOs/organizations

One organizer mentioned, *"Many of these special schools maintain health records for each child, which is helpful for us to understand the overall health of the child. It would be good if every school followed this habit."* There are several good practices that were mentioned by the organizers, to have been followed by the screening teams themselves. One organizer said, *"We make sure that the parents understand the condition of their child's eye and it is also easy for us to create awareness, if the parents are present during examination."*

One organizer mentioned, *"Before every school screening program in special schools, the parents must be informed and encouraged to be present during the program. This will also improve the child's cooperation level."* Another organizer said, *"If government could give some financial or other incentives to the volunteering NGOs, they will be motivated to participate in special school screening programs."*

One organizer said, "We ourselves find it difficult to procure low vision devices if needed as these are not easily available and also very expensive."

DISCUSSION

Universal eye health and the Sustainable Development Goal 3 “to ensure healthy lives for all people at all ages” cannot be achieved without ensuring equitable access to eye health care for underserved groups.(24) This is the first study in India to report on special school managers’ knowledge, attitudes, and practices about the eye health needs of children with SEN. Overall, the findings point to a varied level of understanding, however, heads of special schools can be catalysts for change, in their own schools and among their peers; existing evidence suggests increased eye health knowledge leads to better health-seeking behaviour among parents, school managements.(18, 19)

Encouragingly, most participants said they were aware of the number of children in their schools with eye conditions and that early intervention could result in better eye health outcomes. However, this claim could only be validated by identifying the true numbers by carrying out a prevalence survey in these schools. Additionally, if this is the case, then educating the staff to encourage/remind the parents to take their children for regular eye check-up would enable continuum of care for children with eye conditions. This is especially important if there are no eye health programs organized in schools. Even though most participants knew that children with SEN needed regular eye check-up, less than a third of schools reported to have conducted eye health programmes in the past, which is lower than the 53% reported in a similar study from Wales, UK in 2014.(13)

Although, refractive error is one of the most common conditions reported in children with SEN,(25, 26), only less than a third of participants were aware of this condition. This is alarming as uncorrected refractive error could lead to avoidable VI which would further amplify any difficulties caused by a pre-existing disability. Conversely, if corrected in time, it could improve the quality of life for these children.(27-29) All participants said poor nutrition could cause eye problems, but none of the participants mentioned vitamin A deficiency (VAD). Perhaps including VAD specifically in the questionnaire MCQs would have impacted the responses on the causes reported. This may also imply that many participants were

unaware of the importance of vitamin A for good eye health, which might directly impact the diet provided in these schools. However, few schools where meals were provided included vitamin A rich items such as dairy, meat, egg, and green vegetables. Perhaps, these claims cannot be directly attributed to their knowledge about the nutrition pertaining to good eye health alone but overall health. Although there are few studies reporting VAD in children with SEN, one study reported low vitamin A levels among Autism spectral disorders from China in 2021.(30) However, specific knowledge on the role of nutrition and eye health among this group is seems to be limited. This also warrants active inclusion of health promotion as part of special SEH programs in the future.

Most participants exhibited a negative attitude towards the ability of vision-impaired children to perform academic tasks. This could negatively impact eye health-seeking behaviour or referral practices among school staff. In many circumstances, management of a child's overall disability status takes precedence over eye health-related issues and thereby affecting the eye health seeking behaviour among the stakeholder groups.(31) Almost two thirds of the participants thought children with only vision impairment could study in mainstream schools assuming that these schools are inclusive. They also felt studying with normal peers could improve their psychosocial well-being. Although this claim is supported by studies from high-income countries (32), the evidence in LMICs is inadequate.(33)

Apart from creating adaptive environments in schools, many participants said they provided non-optical assistive devices such as large print reading materials, bright coloured objects, and tactile learning materials such as Braille printed reading materials. In addition, they also mentioned that they would encourage the children to use their spectacles and allow them to use prescribed low vision devices. Such practices are encouraging and should be included in health education programs. The varied level (mostly low) of knowledge about the eye health needs of children with SEN among SSM in Hyderabad is comparable to reports from mainstream primary schoolteachers outside India,(17, 18, 34) however, it does not correlate with mainstream primary schools in India.(16, 19) This could be due to higher coverage of

mainstream schools by SEH programmes in India compared to special schools, which might have had a positive impact on the awareness level among mainstream schoolteachers which was also reported in China and Vietnam.(35, 36) Alternatively, this disparity could also be due to the inclusion of school heads in this study rather than school/class teachers.

This study also identified barriers for organizing eye health programs in special schools. The SEH programme organizers reported that the inclusion of an eye examination in annual general health check-ups was one of the main reasons for the low coverage of special schools in SEH programmes. However, this was not reported by the SSM staff. This misalignment could be a crucial component of any service coverage gap. Future research should prioritise assessing the frequency and ophthalmic content of general health check-ups at special schools, as well the competency of the personnel doing them to reliably assess the eye health and vision of children with SEN. Should check-ups be found to be insufficient to fully meet the needs of children with SEN, both schools and programme organisers will have a greater incentive to prioritise inclusion of special schools in broader programme activities.

The organizers reported various barriers for implementing programmes in special schools which included limited lack of ownership, skilled human resources, difficulty in task-shifting, unaffordability, and availability of LVDs. A systematic review in 2018, reporting on the interventions to improve school-based eye care in LMICs also reported similar findings.(37) Training of skilled eye health workers such as optometrists in assessing children with SEN, prescription of glasses and LVDs in children and incentivizing them for attending special school eye health programmes may help in addressing the issue of unavailability of human resource problems.(38) Additionally, facilitating availability of LVDs and making them accessible and affordable for these children are some of the aspects that needs to be worked out at policy makers' level. Furthermore, increasing eye health awareness among special school managers, schoolteachers and parents would help in better referral uptake of

services and improve continuum of care.(31, 38) Hence, there is a need for appropriate referral set ups in their proximity for the referred children to avail these services.

Lack of stakeholder (teachers/parents/guardians) engagement in SEH programme was another major concern reported. Lack of motivation among teachers was reported on the assumption that they are already overburdened with other health programmes and responsibilities, a scenario also reported in Pakistan.(39) Perhaps incentivizing the teachers or staff for taking up such responsibilities might improve this situation.(38) The organizers reported that there is a limited opportunity to conduct eye health awareness sessions for the primary stakeholders (parents/guardians), as most of them do not attend the screening programmes. This may also have a significant impact on the continuum of care due to poor referral uptake.(31)

The organizers also made several recommendations to improve KAP of stakeholders and to increase uptake of eye health programmes in special schools. They suggested that incorporating health promotion strategies within SEH programmes could make them more impactful and might indirectly address the issue of sustainability.(31) When the stakeholders are better informed about the eye health needs of these children, it would positively impact their eye health seeking behaviour, similar to studies in mainstream schools from China and Vietnam.(35, 36) Additionally, financial subsidiaries or free of charge spectacles/LVDs could also reduce the burden on these families who might otherwise be facing increased health care costs associated with caring for children with SEN.(40)

Limitations

This study should be interpreted in the context of its limitations. Firstly, the participants recruited were the school heads from a minority of special schools in the Hyderabad area and the participants of these institutions may not be representative of all headteachers/schools in the area. Secondly, the study period was interrupted by COVID-19 travel restrictions, meaning face-to-face sensitization and enrolment of headteachers was

not possible. Thirdly, although the questionnaire was piloted among the peers of the principal investigator, it was not validated for the target audience, which might have resulted in varying levels of understanding between participants. Finally, the day-to-day engagement of the headteachers with the children in their schools was unverifiable and it might have been valuable to have recruited class teachers for more insight into the daily activities and challenges faced by the children, however, this was hampered by the pandemic which posed difficulty in contacting the class teachers.

Conclusion

This study highlights a potential gap in the knowledge, attitude and practice of special school managers and barriers to organize SEH programmes in special schools in India. Measuring the eye health awareness level and KAP among schoolteachers in a larger sample would give a better insight into the matter. It further demands inclusion of eye health promotion strategies to create eye health awareness among all the stakeholder groups. This study also highlights barriers to organize SEH programmes in special schools that included accessibility and affordability of eye health services. Thus there is a need to conduct a situational analysis to estimate the prevalence of VI and other eye conditions to understand the current need and service gap for children with SEN.

Acknowledgements

The authors would like to acknowledge the help received from Ms.Mahalaxmi Mojjada for translating the consent form and questionnaire into Telugu language. The authors also would like to thank all the participants for their valuable time responding to the questionnaire and the interview.

Financial support

This study was carried out as part of MSc thesis at London School of Hygiene and Tropical Medicine (LSHTM), Keppel Street, London, UK. The study was supported by British Council for Prevention of Blindness, LSHTM, and Commonwealth eye health consortium.

Conflicts of interest

There are no conflicts of interests.

References

1.Das M, Spowart K, Crossley S, Dutton GN. Evidence that children with special needs all require visual assessment. Arch Dis Child. 2010;95(11):888-92.

2.Salt A, Sargent J. Common visual problems in children with disability. Arch Dis Child. 2014;99(12):1163-8.

3.Disabled Persons in India a statistical profile 2016. Ministry of Health and family welfare; 2016.

4.Andersen ES, Dunlea A, Kekelis LS. Blind children's language: resolving some differences. J Child Lang. 1984;11(3):645-64.

5.Hatton DD, Bailey DB, Burchinal MR, Ferrell KA. Developmental Growth Curves of Preschool Children with Vision Impairments. Child Development. 1997;68(5):788-806.

6.Molloy A, Rowe FJ. Manneristic Behaviors of Visually Impaired Children. Strabismus. 2011;19(3):77-84.

7.Kempen GI, Ballemans J, Ranchor AV, van Rens GH, Zijlstra GA. The impact of low vision on activities of daily living, symptoms of depression, feelings of anxiety and social support in community-living older adults seeking vision rehabilitation services. Quality of life research : an international journal of quality of life aspects of treatment, care and rehabilitation. 2012;21(8):1405-11.

8.Kulkarni SK, Veer AB, Gaikwad A, Deshpande M. The impact of early intervention on a child with vision loss in infancy - A parental perspective. Indian journal of ophthalmology. 2022;70(9):3377-81.

9.Education for children with disabilities- improving access and quality , UKAID, Department of International Development ; https://assets.publishing.service.gov.uk/government/uploads/system/uploads/attachment_data/file/67664/edu-chi-disabil-guid-note.pdf.

10.Frick KD, Riva-Clement L, Shankar MB. Screening for refractive error and fitting with spectacles in rural and urban India: cost-effectiveness. Ophthalmic epidemiology. 2009;16(6):378-87.

11.Lester BA. Comparing the cost-effectiveness of school eye screening versus a primary eye care model to provide refractive error services for children in India. Community Eye Health. 2007;20(61):15.

12.Saravanan S, Krishnamurthy S, Ramani KK, Narayanan A. Cost Analysis of a Comprehensive School Eye Screening Model from India. Ophthalmic epidemiology. 2022:1-7.

13.Woodhouse JM, Davies N, McAvinchey A, Ryan B. Ocular and visual status among children in special schools in Wales: the burden of unrecognised visual impairment. Arch Dis Child. 2014;99(6):500-4.

14.Donaldson LA, Karas M, O'Brien D, Woodhouse JM. Findings from an opt-in eye examination service in English special schools. Is vision screening effective for this population? PLoS One. 2019;14(3):e0212733.

15.Allen LC, Dillon A, Bowen P. Eye Care for Children in Special Schools: An Audit of Provision. The British and Irish orthoptic journal. 2021;17(1):27-32.

16.Ambika K, Nair N. A Study on Awareness of Primary School Teachers Regarding Refractive Errors and its Early Identification among Primary School Children. International Journal of Nursing Education. 2013;5:6-9.

17.Habiba U, Ormsby GM, Butt ZA, Afghani T, Asif M. Knowledge and practices of teachers associated with eye health of primary school children in Rawalpindi, Pakistan. Taiwan J Ophthalmol. 2017;7(1):28-33.

18.Maru A, Belete G, Adimassu N. Knowledge, attitude and associated factors among primary school teachers regarding refractive error in school children in Gondar city, Northwest Ethiopia. PLOS ONE. 2018;13:e0191199.

19.Thomas A. A comparative study to assess the knowledge of primary school teachers regardingrefractive errors among primary school children in selected urban and rural schools, Mangalore: RajivGandhi University of Health Science. 2013.

20.Cassetti V, Sanders T, Bruce A. Challenges of Eye Health Care in Children and Strategies to Improve Treatment Uptake: A Qualitative Study from the Perspective of Eye Care Professionals in the UK. British and Irish Orthoptic Journal. 2019.

21.Paudel P, Yen PT, Kovai V, Naduvilath T, Ho SM, Giap NV, et al. Effect of school eye health promotion on children’s eye health literacy in Vietnam. Health Promotion International. 2017;34(1):113-22.

22.Archana Vishwakarma PG, Rupsha Mitra, Sajid Ali. Teachers' perspective, awareness and understaning of Student's eye health, Tech Mahindra Foundation, New Delhi. 2021.

23.Braun V, Clarke V. Using thematic analysis in psychology. Qualitative Research in Psychology. 2006;3:77-101.

24.The Sustainable Development Goals Report 2023: Special Edition: United Nations; 2023 [Available from: https://unstats.un.org/sdgs/report/2023/The-Sustainable-Development-Goals-Report-2023.pdf.

25.Gogate P, Soneji FR, Kharat J, Dulera H, Deshpande M, Gilbert C. Ocular disorders in children with learning disabilities in special education schools of Pune, India. Indian journal of ophthalmology. 2011;59(3):223-8.

26.Pehere NK, Khanna RC, Marlapati R, Sannapaneni K. Prevalence of ophthalmic disorders among hearing-impaired school children in Guntur district of Andhra Pradesh. Indian journal of ophthalmology. 2019;67(4):530-5.

27.Alvarez-Peregrina C, Sánchez-Tena M, Andreu-Vázquez C, Villa-Collar C. Visual Health and Academic Performance in School-Aged Children. Int J Environ Res Public Health. 2020;17(7).

28.Burton MJ, Ramke J, Marques AP, Bourne RRA, Congdon N, Jones I, et al. The Lancet Global Health Commission on Global Eye Health: vision beyond 2020. The Lancet Global health. 2021;9(4):e489-e551.

29.Jan C, Li SM, Kang MT, Liu L, Li H, Jin L, et al. Association of visual acuity with educational outcomes: a prospective cohort study. The British journal of ophthalmology. 2019;103(11):1666-71.

30.Wen J, Yang T, Zhu J, Guo M, Lai X, Tang T, et al. Vitamin a deficiency and sleep disturbances related to autism symptoms in children with autism spectrum disorder: a cross-sectional study. BMC pediatrics. 2021;21(1):299.

31.Cassetti V, Sanders T, Bruce A. Challenges of Eye Health Care in Children and Strategies to Improve Treatment Uptake: A Qualitative Study from the Perspective of Eye Care Professionals in the UK. The British and Irish orthoptic journal. 2019;15(1):96-104.

32.Price R. Inclusive and special education approaches in Developing countries: https://assets.publishing.service.gov.uk/media/5c6ac403ed915d4a39787401/373_Inclusive_and_Special_Education_Approaches.pdf. 2018.

33.Hannah Kuper AS, Howard White. Rapid Evidence Assessment (REA) of What Works to Improve Educational Outcomes for People with Disabilities in Low- and Middle-Income Countries. International Centre for Evidence in Disability, London School of Hygiene and Campbell Collaboration. 2018.

34.Okoloagu N, Okoye O, Onwubiko S, Eze C, Eze B, Chuka-Okosa C. A Survey of Teachers’ Knowledge, Attitudes, and Practices Related to Pupils’ Eye Health and School-Based Eye-Health Services. Nigerian Journal of Ophthalmology. 2019;27(2):68-75.

35.Liu S-M, Chang F-C, Chen C-Y, Shih S-F, Meng B, Ng E, et al. Effects of Parental Involvement in a Preschool-Based Eye Health Intervention Regarding Children’s Screen Use in China. International Journal of Environmental Research and Public Health. 2021;18(21):11330.

36.Paudel P, Yen PT, Kovai V, Naduvilath T, Ho SM, Giap NV, et al. Effect of school eye health promotion on children's eye health literacy in Vietnam. Health Promot Int. 2019;34(1):113-22.

37.Burnett AM, Yashadhana A, Lee L, Serova N, Brain D, Naidoo K. Interventions to improve school-based eye-care services in low- and middle-income countries: a systematic review. Bulletin of the World Health Organization. 2018;96(10):682-94d.

38.Zhao J, Wang H, Guan H, Du K, Zhang Y, Congdon N. Impact of Teacher Incentive Intervention on Students' Vision Healthcare Uptake: A Cluster-Randomized Controlled Trial. Int J Environ Res Public Health. 2022;19(19).

39.Bechange S, Gillani M, Jolley E, Iqbal R, Ahmed L, Bilal M, et al. School-based vision screening in Quetta, Pakistan: a qualitative study of experiences of teachers and eye care providers. BMC public health. 2021;21(1):364.

40.Malvankar-Mehta MS, Wilson R, Leci E, Hatch K, Sharan S. Cost and quality of life of overlooked eye care needs of children. Risk management and healthcare policy. 2018;11:25-33.

Dear Reviewers,

We would like to thank each of the reviewers for their valuable, detailed review and inputs on this manuscript. It has been an excellent learning experience throughout the process of reanalysis and manuscript writing with your inputs. Please find below the responses for each of the comments and inputs from each of the reviewers. We will be very happy to clarify/modify if there are any additional changes or information that need to be additionally added to the manuscript.

Reviewer 1:

1.Title: must better reflect the study which has a KAP component and barriers and enablers to eye care.

Thank you for taking the time to review our manuscript and giving your feedback. We have revised the title to the following “A cross-sectional survey of eye health knowledge, attitude, and practice among special school managers and barriers to eye health programmes in special schools in Hyderabad, India”

2.Methodological concerns: The study is described as mixed methods, but the data is not managed or presented clearly according to requirements of each method. There needs to be clarification between the quantitative analysis and thematic analysis used in the qualitative part.

This study as stated in the manuscript was a mixed method design which included quantitative component and qualitative components. The study consisted of an online questionnaire to capture the knowledge, attitude and practice among the special school managers and an interview of eye health program organizers to understand the barriers to special school eye health programs. The questionnaire consisted of multiple-choice questions and open-ended text boxes. The participant demographics and responses for the multiple-choice questions were analysed as quantitative data for presenting summary statistics. Responses in the open-ended text format and interviews were analysed as qualitative data. The open-ended text responses were categorized under common subthemes and presented within the tables as summary statistics whereas the interview responses were coded and categorized under eight major themes and presented as thematic analysis. Additionally, the participant responses in both open-ended text as well as interviews were stated as quotes in the results section. We did not conduct any inferential statistics as the main objective of the study was not to understand any correlations.

Although, we do agree that an inferential would have added more value to this study, unfortunately we were unable to collect all the information needed to conduct an inferential statistic due to the COVID-19 imposed travel restrictions during the study period. However, we intend to study this in detail as we have secured a grant to conduct a large-scale eye health program in special schools in the study area.

The selection criteria applied must be more clearly articulated to clarify steps and sampling strategy eg. whether all managers or only one leader in each school were invited to participate etc.

Any special school in the study area with valid contact details was eligible for inclusion. Organizations such as early intervention centres, therapy centres, homes for the disabled and schools for the blind were excluded. Within schools, only headteachers, directors or principals who were directly involved in school management activities (referred to in this study as ‘managers’) were eligible for inclusion. Among the managers who could be contacted via the valid contact details were included; typically one manager from each organization. SEH program organizers with a minimum of two years’ experience in community eye health activities from organization that are active in conducting community eye health activities in the Hyderabad region were eligible for inclusion.

The questionnaire design must be detailed. ie. the basis for inclusion of specific questions in each area of the questionnaire and how were these validated for inclusion.

The participants were emailed a link to an online questionnaire built with Google Forms. The questionnaire included some questions used in previous studies conducted in mainstream schools (references given below), plus additional questions written for this study. The questionnaire included

demographic details and questions structured to capture KAP with regards to the eye health needs of children attending special schools. It included multiple-choice questions and open-ended text boxes to allow participants to express their views.

References

1.Ambika K, Nair N. A Study on Awareness of Primary School Teachers Regarding Refractive Errors and its Early Identification among Primary School Children. International Journal of Nursing Education. 2013;5:6-9.

2.Habiba U, Ormsby GM, Butt ZA, Afghani T, Asif M. Knowledge and practices of teachers associated with eye health of primary school children in Rawalpindi, Pakistan. Taiwan J Ophthalmol. 2017;7(1):28-33.

3.Maru A, Belete G, Adimassu N. Knowledge, attitude and associated factors among primary school teachers regarding refractive error in school children in Gondar city, Northwest Ethiopia. PLOS ONE. 2018;13:e0191199.

4.Thomas A. A comparative study to assess the knowledge of primary school teachers regardingrefractive errors among primary school children in selected urban and rural schools, Mangalore: RajivGandhi University of Health Science. 2013.

5.Archana Vishwakarma PG, Rupsha Mitra, Sajid Ali. Teachers' perspective, awareness and understaning of Student's eye health, Tech Mahindra Foundation, New Delhi. 2021.

3.The Results for the quantitative analysis should include some inferential analysis e.g. were there associations between knowledge and practices, or attitudes and practices and were these significant.

We agree that conducting an inferential analysis would add significant weightage to the study and would give meaningful information on the KAP. However, this study was not designed for inferential analysis. Additionally, the number of participants recruited does not allow for meaningful tests of association or statistical significance. Therefore we have not included such results in this study.

Qualitative analysis results must be presented in more detail with the emergent Themes and the specific codes per theme explained and presented as such. Quantitative data is referred to as themes, hence causing confusion.

The thematic analysis of the interview responses was coded and categorized under two major themes as Barriers and Suggestions. Under these major themes, subcategories were explained in detail (The subcategories as follows: program coverage, Program initiative, Lack of skilled human resource, Lack of stakeholder engagement, Impossible to task shift to non-specialists, Poor availability and accessibility of spectacles and low vision devices)

The questionnaire responses were listed as summary tables as explained above in response to question 2. We have modified these as per the suggestions.

4.The Discussion will be strengthened if the methods and results are sorted out.

We have made several changes to the discussion section as per the re-analysis of the data and the results. Pertaining to both the questionnaire and interview responses.

For e.g: From the questionnaire point of view we have added the following points “Almost all participants said poor nutrition could cause eye problems, but none of the participants mentioned vitamin A deficiency (VAD). Perhaps including VAD specifically in the questionnaire MCQs would have impacted the responses on the causes reported. This may also imply that many participants were unaware of the importance of vitamin A for good eye health, which might directly impact the diet provided in these schools. However, few schools where meals were provided included vitamin A rich items such as dairy, meat, egg, and green vegetables. These claims cannot be directly attributed to their knowledge about the nutrition pertaining to good eye health but overall health. Although there are few studies reporting VAD in children with SEN, one study reported low vitamin A levels among Autism spectral disorders from China in 2021. However, specific knowledge on the role of nutrition and eye

health among this group is seems to be limited. This also warrants active inclusion of health promotion as part of special SEH programs in the future.

Apart from creating adaptive environments in schools, many participants said they provided non-optical assistive devices such as large print reading materials, bright coloured objects, and tactile learning materials such as Braille printed reading materials. In addition, they also mentioned that they would encourage the children to use their spectacles and allow them to use prescribed low vision devices. Such practices are encouraging and should be included in health education programs

From the interviews point of view the following statements have been amended and added to the discussion: For e.g. The SEH programme organizers reported that the inclusion of an eye examination in annual general health check-ups was one of the main reasons for the low coverage of special schools in SEH programmes. However, this was not reported by the SSM staff. This misalignment could be a crucial component of any service coverage gap. Future research should prioritise assessing the frequency and ophthalmic content of general health check-ups at special schools, as well the competency of the personnel doing them to reliably assess the eye health and vision of children with SEN. Should check-ups be found to be insufficient to fully meet the needs of children with SEN, both schools and programme organisers will have a greater incentive to prioritise inclusion of special schools in broader programme activities.

Lack of stakeholder (teachers/parents/guardians) engagement in SEH programme was another major concern reported. Lack of motivation among teachers was reported on the assumption that they are already overburdened with other health programmes and responsibilities, a scenario also reported in Pakistan. The organizers reported that there is a limited opportunity to conduct eye health awareness sessions for the primary stakeholders (parents/guardians), as most of them do not attend the screening programmes. This may also have a significant impact on the continuum of care due to poor referral uptake.

5.The Conclusion should highlight findings in relation to the key study objectives and recommendations.

We have added the following as the conclusion as per suggestion, "This study highlights a potential gap in the knowledge, attitude and practice of special school managers and barriers to organize SEH programmes in special schools in India. Measuring the eye health awareness level and KAP among schoolteachers in a larger sample would give a better insight into the matter. It further demands inclusion of eye health promotion strategies to create eye health awareness among all the stakeholder groups. This study also highlights barriers to organize SEH programmes in special schools that included accessibility and affordability of eye health services. Thus there is a need to conduct a situational analysis to estimate the prevalence of VI and other eye conditions to understand the current need and service gap for children with SEN."

6.There are minor language edits needed as tracked in the manuscript.

We have attempted to improve the writing style throughout the manuscript as part of this revision.

Reviewer 2

Main comments:
Many thanks for a thought-provoking article. Establishing a connection between the responses from the two groups of participants, namely teachers and School Eye Health (SEH) organizers, would provide valuable insights. Exploring teachers' perspectives on the SEH organizers' list of barriers and enablers could shed light on their perceptions and potential collaboration challenges. Notably, the organizers suggest that all children should undergo either annual or admission eye checks. It is essential to examine whether this recommendation is implemented in practice. If the organizers assert that SEH programs are unnecessary because eye care already takes place, it raises the question of whether this belief constitutes the primary barrier to effective eye care, particularly if annual checks or checks on admission or not occurring for everyone. In light of these considerations, the research could benefit from focusing less on theoretical assessments of the perceived importance of eye care and instead prioritize investigating the current state of eye care in India. Questions that address the percentage of children who have received comprehensive eye examinations, the timing of their most

recent tests, the prevalence of spectacle usage or eye conditions among the children, and a comparison with existing studies would enhance the understanding of the current situation.

Thank you very much for your review and valuable suggestions for future research in this area. We agree that an important next step is to report on the status of children's eye examinations, eye health and spectacle use. Due to the COVID-19 restrictions, studying the current state of eye care in special schools was not possible. Reaching out to the schoolteachers rather than the managers was also not possible. We have secured a grant to conduct a large-scale school eye health program in special schools in this region. As part of this project, we intend to collect data on the status of availability of services and compare the results with the level of knowledge of schoolteachers. We would also like to understand any correlation in the vision impairment and refractive errors, prevalence of spectacle usage and the awareness levels. We also would be able to study the spectacle compliance among these children.

Introduction:
1. School eye health (SEH) programs have the potential to provide children with access to good quality services and can be cost-effective (9-11). It would be helpful to specify which services are included in SEH programs.

The sentence has been amended as following: School eye health (SEH) programmes have a potential to provide children with access to high quality eye care services including refractive error correction and additional referral services and it can be cost-effective.

2. In a study by Woodhouse et al. in 2014 (12), it was found that only 53% of special schools in Wales, UK, had conducted eye health programs. It would be beneficial to include data from India on this topic, or mention if no data is available.

There are no such studies or reports available from Indian context and we have included the same in the manuscript as well.

3. In the introduction, it would be valuable to compare the prevalence of eye problems in children with autism, children with multiple disabilities, and children with no disabilities. Additional information and references from Donaldson papers and the Seeability website could be included.

We have added several study references to the introduction section (References 1, 9-22) on various disabilities which highlight the need for eye care interventions in among children with autism, intellectual impairment, hearing impairment and cerebral palsy both within and outside India.

4. The reader needs to know what currently happens in India and in each school included in the research. It would be relevant to mention whether each child has received an eye examination or screening test, and what those tests consisted of. Details about vision tests, cover tests, ocular motility, BV tests, cycloplegia, refraction, and fundus and media examinations would be informative. Additionally, it would be helpful to clarify the procedures followed if a child is unable to respond to a letter test, such as using pictures or preferential looking tests, or referring the child to a hospital.

We acknowledge the importance of including these elements in the data collection process. Although our original aim was to conduct a cross sectional study in a sample of these schools in India, COVID-19 restrictions at the time of the study disrupted the proposed examination of children. This is also the main reason for difficulty in contacting the schools and the teachers online as the principal investigator was in the UK at the time of the study. We will certainly consider these components to be added in our future research in this area.

5. Special school managements- Is this Special school managers/head teachers?

This included headteachers, directors and principals. Everyone had a different title although they hada similar roles in their schools which is the role of a head teacher.

6. Please check SEH written in full in the first instance.

This has been corrected as per the suggestion

7. Sometimes SHE used. This has been corrected as per the suggestion

8. Throughout the paper, it should be explicitly addressed that the study assesses the knowledge and attitudes of school managers and the barriers and enablers of organizers to SEH. This should be reflected in the title as well.

We have modified the title as per the suggestion as follows "A cross-sectional survey of eye health knowledge, attitude, and practice among special school managers and barriers to eye health programmes in special schools in Hyderabad, India"

Methodology:
9. In Table 1, it may be more effective to present the 13 schools separately rather than together.

Thank you for the suggestion. While it is a very good idea to do so, we presented it as a small table as the other tables are very large and may not be appealing to the reader. However, we can modify this if the reviewer feels it would add more value.

10. Table 1 specify years for age.

Included as suggested

11. Instead of focusing on theoretical scenarios, it could be more realistic to ask schools about the eye care provided to the children currently under their care. A theoretical methodology is likely to lead to positive subjective bias.

We agree with this point. We will consider this in our future research which we will be conducting for the special schoolteachers in the study area. We welcome any suggestions towards the new methodology, and it will be valuable for us for the future research in the area as we have received a grant to pursue research in this area.

12. The number of school managers who are aware of children with an eye condition does not mean that they have identified all the children with eye conditions if a full sight test not carried out on all children.

This is true. We also believe that unless an eye examination is carried out in these schools it will be difficult to ascertain this merely based on the response from these participants. This we believe will be addressed in the future research.

13. Stating that health programmes are warranted twice a year does not mean they occur, particularly for all children. Many people are under the impression that some children are too disabled for a sight test. What is included in a health programme?

This is very true. However, the aim of this question was to understand what these participants felt it to be appropriate, not to understand how many times a year they had conducted in the past. We did not ask the question pertaining to the annual health check-up. As the questionnaire responses were conducted prior to the interviews. Perhaps conducting the organizer interviews before creating the

questionnaire would have helped in framing the questions related to past health/eye health programs in the schools.

14. It would be valuable to determine the percentage of children who have received an eye test or screening program. Which type was it, how long ago it occurred, and the average age at the first test.

Thank you for the suggestion. We will consider this in our future research.

15. The study should also inquire if each child has a documented sight test in their clinical records and if there is a management plan for any diagnosed conditions.

Thank you for the suggestion. We will consider this in our future research.

16. Comparing the number of visually impaired children, those wearing refractive correction, or diagnosed with eye conditions to other populations of children with special educational needs (SEN) would provide useful insights. If significantly lower numbers are found, it suggests that schools are not identifying all cases.

This is the next step in our future research. Conducting eye health programs in the special schools and identifying the eye health awareness levels among schoolteachers. Thank you for reiterating these important points.

17. It would be beneficial to inquire if carers understand the implications for each child diagnosed with an eye condition and if they felt they required further education. For example, clarification could be sought regarding whether a child with freely alternating manifest strabismus would be classified as visually impaired and require magnification or if it simply affects their coordination.
18. It would be pertinent to ask how often each child's glasses should be checked and what the procedure is in case of breakage.

Thank you very much for your helpful suggestions in points 11-18 for future research building on this initial study of knowledge, attitudes, and practice among schoolteachers.

19. Online questionnaire should be included.

Thank you for this suggestion. We have included the questionnaire as supplementary material.

Results:
20. It is unclear why there is a discrepancy in the responses of participants 1 and 2 regarding the frequency of eye check-ups (yearly or on admission). Clarification should be sought from all schools to confirm if children receive an annual eye health assessment or one on admission, as stated by the health organizers. Additionally, it would be important to determine if any children fall through the gaps and do not receive an eye test.

As mentioned in question 13, the questionnaire responses were conducted prior to the interviews. Perhaps conducting the organizer interviews before creating the questionnaire would have helped in framing the questions related to past health/eye health programs in the schools. Also we feel that the different responses from two participants could be due to difference in the level of priority that each of their organizations might be having to conduct school eye health programs each year.

21. It would be relevant to explore if schools are aware that they can request an SEH program, as stated by the organizers.
22. The schools' perspectives on the barriers stated by the organizers should be included. Do they feel unwilling to participate?
23. It should be clarified if the schools are allowed to refer children to a doctor and, if so, which type of

doctor.
24. Environmental modifications or adaptations mentioned in the results should be explained.
25. The SEH organizers could be asked if the identified barriers impact the child's annual or admission eye check.
26. If the organizers believe that SEH is unnecessary because eye care already occurs, it raises the question of why they are concerned about barriers to SEH.

This is a very valid point. Thank you for highlighting this point. This must also be included in the future research questionnaire item.

Discussion:

27. When children have difficulties with reading, writing, and multiple disabilities, it can be challenging for caregivers to determine whether the issues stem from eye problems, IQ problems, autism, or other factors related to their disabilities. It would be helpful to address whether all children struggling are referred to eye specialist even if carers are unsure what the problem is.

28. The SEH organizers should be checked whether they have verified if each child receives their annual or admission eye check-up, or if they assume that it happens.

Thank you very much for your helpful suggestions in points 20-28 for future research building on this initial study.

Reviewer 3

This is an interesting and useful contribution to understanding the barriers to service provision in special schools.

Thank you very much for your review and feedback.

My comments:

1) Line 81-84. In the introduction the authors refer to eye care programs in Wales. There is now a wider England study that is driving the debate that I think is worth considering.
Findings from an opt-in eye examination service in English special schools. Is vision screening effective for this population? L. A. Donaldson, M. Karas, D. O'Brien and J. M. Woodhouse. PloS one 2019 Vol. 14 Issue 3 Pages e0212733
I would also like to see this idea explored more as to the relevance or not of this single example of a program in the UK.

Thank you for you suggestion. We have included the above citation as well we found another relevant study. These both studies highlight different sides of the problems 1) Donaldson LA et al presents the provision for providing eye care for children with SEN among the eye care providers whereas 2) Woodhouse et al, presents the problem from the special schools' point of view as how often they conducted eye health programs in schools. We have highlighted these studies and elaborated in the introduction section.

2) The study defines itself as a mixed methods study (line 98-99) but it is hard to work out how the data was handled? It talks of a thematic analysis, and I am assuming this is of both the open text parts of the online questionnaires and the recorded interviews. (Line 148-148). This needs to be clarified. It then goes onto to say the questionnaire data themes were determined but says nothing of the interview thematic analysis which you would expect to lead to some other themes.

In the results section it then discusses results under each of these three themes defined in the methods section, but this data seems to be only the results of knowledge, practice, and attitude multiple choice questions. I cannot see any discussion of the results of the thematic analysis of the free text responses.

This study as stated in the manuscript was a mixed method design which included quantitative component and qualitative components. The study consisted of an online questionnaire to capture the knowledge, attitude and practice among the special school managers and an interview of eye health program organizers to understand the barriers to special school eye health programs. The questionnaire consisted of multiple-choice questions and open-ended text boxes. The participant demographics and responses for the multiple-choice questions were analysed as quantitative data for presenting summary statistics. Responses in the open-ended text format and interviews were analysed as qualitative data. The open-ended text responses were categorized under common subthemes and presented within the tables as summary statistics. Additionally, the participant responses in both open-ended text as well as interviews were stated as quotes in the results section.

The thematic analysis of the interview responses was coded and categorized under two major themes as Barriers and Suggestions. Under these major themes, subcategories were explained in detail (The subcategories as follows: program coverage, Program initiative, Lack of skilled human resource, Lack of stakeholder engagement, Impossible to task shift to non-specialists, Poor availability and accessibility of spectacles and low vision devices)

3) The results of the interviews do offer quotes as would be expected of a thematic analysis and the data does seem to be grouped into headings but there is no mention made of themes generated form an analysis of this data.

We have included quotes as suggested from both questionnaire as well as interviews

4) There is no discussion of the methodology of the thematic analysis. How was it planned, how was the data coded and how was a thematic framework arrived at. There is also no mention of the theoretical underpinnings of the approach to the thematic analysis. This is a complex subject, but I feel if the authors a claiming a thematic methodology they should make some effort to describe within the bounds of accepted thinking on this method. (Braun & Clarke, 2006; Kiger & Varpio, 2020). This does not need to be detailed but it does need a mention.

Thank you for these resources. We have followed the methods suggested by Braun and Clarke for the analysis. We have also included this in the methodology section as reference.
