# Supplementary material for: Eye health knowledge, attitude, and practice among special school managers and barriers to eye health programmes in special schools in Hyderabad, India
Source: PLOS Glob Public Health. 2024 Aug 28;4(8):e0002124. doi: 10.1371/journal.pgph.0002124 (PMC11356432; doi:10.1371/journal.pgph.0002124)
Supplement: Attachment — Submitted filename: REVIEW COMMENTS corrected.pdf [file pgph.0002124.s006.pdf]

MY COMMENTS

GENERAL COMMENTS

This is an interesting study and worth reporting. The manuscript is very well written with least grammatical errors. However, some of the underlisted limitations are ones authors could have corrected easily before commencing the actual data collection. Example is especially ensuring the questionnaire used was reliable and valid to measure exactly what it seeks to measure. I think authors instead of recommending another study to report on a comprehensive situational analysis, should have taken some time to collect data and analyze these findings also to enrich the current report further.

SPECIFIC COMMENTS

Line 39-40 on the old document ; Lines 41 -42 of revised document: Is not grammatically correct to begin a sentence with a number or fraction like this. I suggest authors modify this sentence. E.g. A number of thirteen (13) out of sixty-seven (67) invited SSM (19%) participated.

Line 51 on the old document; Lines 53-54 of revised doc: I think current work should have considered also conducting a comprehensive situational analysis of these findings so discussion would be rich. This is just a scanty result.

Lines 128-130 in old document; Lines 146 – 148 on the revised doc: How are authors sure responders' answers reflect their current understanding. What precautions or measures did author put in place to ensure responders to not refer to other resources on the internet especially as questionnaire stayed with them for two weeks. Please indicate this.

Line 154-155 in old document; Line 172 -173 on revised document: Demographic data like age and gender distributions are repeated in tables and in the text. It will be advisable for authors to delete from the text and to simply refer to the table 1 to avoid repetitions.

Line292 in old document; Line 445 -447 I revised document: The questionnaire used should have at least been tested for its reliability. If these was done in the pilot study, please indicate how reliable this tool was. With use of an unreliable questionnaire, how are we sure of the reliability of the results?
