# Supplementary material for: Eye health knowledge, attitude, and practice among special school managers and barriers to eye health programmes in special schools in Hyderabad, India
Source: PLOS Glob Public Health. 2024 Aug 28;4(8):e0002124. doi: 10.1371/journal.pgph.0002124 (PMC11356432; doi:10.1371/journal.pgph.0002124)
Supplement: Attachment — Submitted filename: PGPH-D-23-01037_R2 EDITOR REVIEW 1.pdf [file pgph.0002124.s008.pdf]

PLOS Global Public Health

Eye health knowledge, attitude, and practice among special school managers and barriers to eye health programmes in special schools in Hyderabad, India

--Manuscript Draft--

**Manuscript Number:**	PGPH-D-23-01037R2
**Article Type:**	Research Article
**Full Title:**	Eye health knowledge, attitude, and practice among special school managers and barriers to eye health programmes in special schools in Hyderabad, India
**Short Title:**	Eye health KAP and Barriers to organize eye health program in special schools
**Corresponding Author:**	Winston Deva Prakash Devaraj, BSc, MSc<br>LV Prasad Eye Institute<br>Hyderabad, Telangana state INDIA
**Order of Authors:**	Winston Deva Prakash Devaraj, MSc
	Priya Morjaria, PhD
	Ian McCormick, MSc
	Rohit Khanna, MD
**Keywords:**	Special needs, special school eye health, Eye health awareness, Barriers to organize school eye health programs, Special needs eye health
**Abstract:**	Background<br>Children with special education needs (SEN) are at higher risk of developing vision problems. In India, there is no data available on the awareness level among special school managers (SSM) about the eye health needs of children with SEN and on barriers for providing eye care for these in children in schools. This study is aimed to identify the awareness level among SSM and barriers for organizing School Eye Health (SEH) programmes in special schools as reported by the eye health program organizers.<br>Methods<br>A mixed method study was conducted between July and August 2020 among SSM and eye health programme organizers from local eye care providers in Hyderabad, India. SSM participants completed an online questionnaire assessing their knowledge, attitude, and practice concerning the eye health needs of children with SEN. Quantitative responses were described with summary statistics. Qualitative interviews with eye health programme organizers were conducted by telephone and transcripts were thematically analysed.<br>Results<br>A total of 13 (19.4%) out of 67 invited SSM participated and 2/4 invited eye health programme organizers (50%) were interviewed. Among the SSM participants, 92.3% were aware of vision impaired (VI) children in their schools. Awareness of potential causes of VI (most to least common cause) ranged from 53.9%-92.3% by cause, common eye conditions 7.7%-69.2% by condition (most to least common condition) and difficulties experienced by children with SEN in classroom activities 46.2%-76.9% by activity (most to least common activity). Only 30.8% of special schools organized SEH programmes at least once a year. Eye health programme organizers reported barriers such as a lack of interest from SSM, unavailability of qualified screening staff, and a lack of provision for spectacles and low vision devices.<br>Conclusion<br>This study identified varied levels of knowledge, attitude, and practice, about the eye health needs of children with SEN, among SSM. Key barriers to conducting SEH programmes included lack of demand, human resource availability and limited access to government-funded resources. Further research is needed to undertake a comprehensive situational analysis and to plan eye health programmes for children with SEN.
**Opposed Reviewers:**	
**Additional Information:**	

Commented [VR1]: delete

Deleted: ,

Commented [VR2]: related to

Powered by Editorial Manager® and ProduXion Manager® from Aries Systems Corporation

Question	Response
**Financial Disclosure**<br><br>Enter a financial disclosure statement that describes the sources of funding for the work included in this submission and the role the funder(s) played. This includes grants and any commercial funding of the work or authors.<br><br>This statement will be typeset if the manuscript is accepted for publication.<br><br>*Please review the submission guidelines and the instructions link below for detailed requirements and guidance.*	This study was carried out as part of a MSc thesis at London School of Hygiene and Tropical Medicine (LSHTM), Keppel Street, London, UK. During the study period the principal investigator (WP) was supported by the British Council for Prevention of Blindness, LSHTM, and Commonwealth eye health consortium. Other authors (PM, IM, RCK) did not receive any financial or in-kind incentives to be a part of this study. The funders had no role in the study design, data collection and analysis, decision to publish, or preparation of the manuscript.
**Competing Interests**<br><br>On behalf of all authors, disclose any competing interests that could be perceived to bias this work.<br><br>This statement will be typeset if the manuscript is accepted for publication.<br><br>*Please review the instructions link below and PLOS Global Public Health's competing interests policy to determine what information must be disclosed at submission.*	The authors have declared no competing interest exists.
**Data Availability**<br><br>Before publication, Authors are required to make fully available and without restriction all data underlying their findings. Please see our PLOS Data Policy page for detailed information on this policy.<br><br>A **Data Availability Statement**, detailing where the data can be accessed, is	Dear Editor,<br><br>Please find the data in the following link:<br><br>https://datadryad.org/stash/share/MP-46peyiUV9qrUdHfass2BLoM8Ankgz-hfFtwBw5fQ<br><br>Thank you,<br>Kind regards,<br>Winston D Prakash (First Author)<br>(On behalf of the authors)

Powered by Editorial Manager® and ProduXion Manager® from Aries Systems Corporation

required at first submission. Insert your Data Availability Statement in the box below.<br><br>Please see the data reporting section of our submission guidelines for instructions on what you need to include in your Data Availability Statement.<br><br>This statement will be typeset if the manuscript is accepted for publication.<br><br>*PLOS allows rare exemptions to address legal and ethical concerns. If you have legal or ethical restrictions, please use the box below to detail these in full sentences for the Journal team to consider.*	

Powered by Editorial Manager® and ProduXion Manager® from Aries Systems Corporation

Cover letter

To
The editor-in-chief,
PLOS Global Public Health,

Respected Sir/Madam,

We would like to publish our original research article titled "**Eye health knowledge, attitude, and practice among special school managers and barriers to eye health programmes in special schools in Hyderabad, India**" in your esteemed journal. We believe that the findings and message from this work is suitable and within the scope of your esteemed journal. The key findings from this study are as follows. This study provides a snapshot into the knowledge, attitude and practice among the key stakeholder groups such as special school managers and eye health service providers. This being a first study in India to identify the eye health knowledge, attitude and practice among special school managers and barriers to organize eye health programs in special schools in Hyderabad from eye health program organizers point of view, we believe the findings from this study will be helpful to understand the status of the availability of eye health service provisions for children with special educational needs in Hyderabad, India.

Key findings in this article are as follows

1. This study identifies the varied level of knowledge, attitude and practice among the special school managers about the eye health needs of children with special educational needs.
2. There are number of barriers to conduct eye health programmes in special schools in Hyderabad, India which include lack of manpower, resources, and treatment provisions.
3. There is a need for increasing eye health awareness among teachers, school managements and parents of children with special educational needs in Hyderabad, India.

Kindly do the needful.

Thank you,
Sincerely,
Winston D Prakash (Email: winston@lvpei.org) - Corresponding author

Eye health knowledge, attitude, and practice among special school managers and barriers to eye health programmes in special schools in Hyderabad, India

Author Information

Winston D Prakash [1,2,3]

Priya Morjaria [2]

Ian McCormick [2]

Rohit Khanna [1,4,5]

Affiliations

1. Allen Foster Community Eye Health Research Centre, Gullapalli Pratibha Rao International Centre for Advancement of Rural Eye care, L V Prasad Eye Institute, Hyderabad, Telangana, India
2. International Centre for Eye Health, London School of Hygiene and Tropical Medicine, Keppel Street, London, UK
3. Brien Holden Institute of Optometry and Vision Science, L V Prasad Eye Institute, Hyderabad, Telangana, India
4. School of Optometry and Vision Science, University of New South Wales, Sydney, Australia,
5. University of Rochester, School of Medicine and Dentistry, Rochester, NY, USA

Corresponding author and address:

6. Mr. Winston D Prakash,
7. E-mail: winston@lvpei.org

ABSTRACT

Background

Children with special education needs (SEN) are at higher risk of developing vision problems. In India, there is no data available on the awareness level among special school managers (SSM) about the eye health needs of children with SEN and on barriers for providing eye care for these children in schools. This study aimed to identify the awareness level among SSM and barriers for organizing School Eye Health (SEH) programmes in special schools, as reported by the eye health program organizers.

Deleted: in

Deleted: is

Methods

A mixed method study was conducted between July and August 2020 among SSM and eye health programme organizers from local eye care providers in Hyderabad, India. SSM participants completed an online questionnaire assessing their knowledge, attitude, and practice concerning the eye health needs of children with SEN. Quantitative responses were described with summary statistics. Qualitative interviews with eye health programme organizers were conducted by telephone and transcripts were thematically analysed.

Commented [VR3]: were these programme organizers chosen from amongst all eye care providers (including private providers) or just from amongst the NPO sector?

Results

A total of 13 (19.4%) out of 67 invited SSM participated, 2/4 invited eye health programme organizers (50%) were interviewed. Among the SSM participants, 92.3% were aware of vision impaired (VI) children in their schools. Awareness of potential causes of VI (most to least common cause) ranged from 53.9%-92.3% by cause, common eye conditions 7.7%-69.2% by condition (most to least common condition) and difficulties experienced by children with SEN in classroom activities 46.2%-76.9% by activity (most to least common activity). Only 30.8% of special schools organized SEH programmes at least once a year. Eye health programme organizers reported barriers such as lack of interest from SSM, unavailability of qualified screening staff, and a lack of provision for spectacles and low vision devices.

Conclusion

This study identified varied levels of knowledge, attitude, and practice about the eye health needs of children with SEN among SSM. Key barriers to conducting SEH programmes included a lack of demand, human resource availability and limited access to government-funded resources. Further research is needed to undertake a comprehensive situational analysis and to plan eye health programmes for children with SEN.

Commented [VR4]: Noting the limitations it may be necessary to include "As the study was negatively impacted by the Covid pandemic, further research with wider representation is needed to plan comprehensive eye health programmes for children with SEN"

Keywords: Disability, eye health, child health, school health, special school eye health

INTRODUCTION

Children with special educational needs (SEN) are at a higher risk of developing vision impairment (VI) and ocular comorbidities.(1, 2) In India, 28% of people living with a disability are under 19 years of age and 18% of these have vision related disability.(3) Vision plays an important role in early childhood development such as hand-eye coordination, communication, spatial comprehension and social development.(4-6) Impaired vision can lead to difficulty in performing activities of daily living (ADL), anxiety and depression,(7), additionally, VI in the presence of other disabilities can amplify these difficulties further. Therefore, early identification of VI and other eye conditions in children with SEN is crucial for better health and development outcomes. (8)

Commented [VR5]: Check spacing. Review the journals manuscript formatting guidelines.

Commented [VR6]: Need to check and apply consistency with spacings

Special schools help children with activities such as communication, interaction, cognition, learning, catering to sensory and physical needs and helping with social and mental health.(9) School eye health (SEH) programmes have a potential to provide children with access to high quality eye care services and can be cost-effective.(10-12) However, there is less evidence to what extent they are run in schools for children with SEN. A study by Woodhouse et al. in 2014,(13) found that only 53% of special schools in Wales, UK, had conducted eye health programmes in their schools. A study in 2019 by Donaldson LA et al,(14) argued that school entry vision screening is ineffective for children in special schools in England, mainly due to low uptake of primary care service and limited cooperation levels. However, such programs might be the only option to reach these children in many low-income settings, owing to the poor parental awareness or availability of basic services such as vision screening and referral services. A recent study by Allen LC et al in 2021, reporting on availability of eye care services for children with SEN found, 31% of service providers did not have provision for these children in their practices.(15) We found no evidence on the proportion of special schools in India that have held eye health programmes.

Deleted: ,

Commented [VR7]: Rephrase. Donaldson et al., (2019) argued that school……

Commented [VR8]: Do not include initials

Need full stop after al. (Allen et al., (2021)

Hyderabad is the capital city of Telangana state in South India and has a population of over 10 million people. There are over one hundred organizations involved in teaching, training,

and providing services for children with SEN. According to a local eye health organization involved in delivering community-based eye health services, most SEH programmes are focused only on mainstream schools (V Sarvepally, personal email communication, 28 January 2020 – email communication available). Among studies on the knowledge, attitude, and practice of mainstream schoolteachers about the eye health needs of children, studies in India showed higher levels compared to those in other countries.(16-19) However, there are no such studies reporting on the level of awareness about the eye health needs of children with SEN and/or knowledge, attitude, and practice (KAP) among the special school managers (SSM). There is also evidence that increased eye health awareness levels among the school staff, children and parents could result in increased service uptake.(20, 21) Therefore, the aims of this study were firstly to identify the level of eye health awareness of among special school managers and secondly, to identify barriers to organizing eye health programmes in special schools, from the perspective of eye health programme organizers.

Commented [VR9]: As per Reviewer 6, you need to explain why SSM were more appropriate to participate as opposed to teachers.

METHODS

Ethics

Ethics approval was obtained from the London School of Hygiene & Tropical Medicine's MSc Research Ethics Committee, London, UK and the Hyderabad Eye Research Foundation, Hyderabad, India. Participation was voluntary and none of the participants were given any financial or other incentives for participating. All participants read and signed an informed consent form in English or Telugu before enrolling. Participants were advised that all responses would be anonymised for reporting.

Study design, participants, and recruitment

This was a mixed methods study consisting of a quantitative and qualitative component: an online questionnaire to assess KAP among SSM, and semi-structured telephonic interviews to understand the barriers to organizing SEH programmes in special schools. For the online questionnaire, participants were representatives of special schools identified from

Deleted: by

Deleted: telephone

a local tertiary eye care centre's rehabilitation centre database **(Figure 1).** Contact details for all identified special schools in Hyderabad were obtained from the database and a publicly available free online resource (www.nayi-disha.org). Schools with valid contact details were invited to participate in the study by a telephone call and an email.

For the qualitative interviews, participants were programme organisers from eye care organizations involved in community outreach activities in urban and sub-urban areas of Hyderabad, India. Programme organizers were invited to participate by email and those who responded were included in the study.

Inclusion and exclusion criteria

Any special school in our area of interest with valid contact details was eligible for inclusion. Organizations such as early intervention centres, therapy centres, homes for the disabled and schools for the blind were excluded. Within schools, only headteachers, directors or principals who were directly involved in school management activities (referred to in this study as 'managers') were eligible for inclusion. Any organization that was active in conducting community eye health activities in the Hyderabad region was eligible for inclusion. Within these organizations, any SEH programme organizer with a minimum of two years' experience was eligible for inclusion.

Data collection

Data collection was carried out between July-August 2020. After giving consent, participants were emailed a link to an online questionnaire built with Google Forms. The questionnaire included some questions used in previous studies conducted in mainstream schools,(16-19, 22) plus, additional questions written for this study. The questionnaire included demographic details and questions structured to capture KAP with regards to the eye health needs of children attending special schools. It included multiple-choice questions and open-ended text boxes to allow participants to express their views **(Supplementary material).** Participants were asked to complete the questionnaire within two weeks and not to refer to the internet,

Commented [VR10]: *Rephrase:* Representatives of special schools identified from a local tertiary eye care centre database completed the online questionnaire.

Commented [VR11]: What is a "valid" contact detail as opposed to one that will be considered "invalid".

Commented [VR12]: The qualitative component included participants ………

Commented [VR13]: Again…need clarification in previous section on "valid contact details" as 39/67 were identified as not valid.

Commented [VR14]: Were these developed and piloted?

Commented [VR15]: The questionnaire included open ended questions as opposed to open ended "text boxes" The text boxes are included for the responses to be inserted, but your questions were "open-ended. Please rephrase throughout.

so their answers reflected their current understanding. Schools who did not respond to calls or emails after three attempts were considered non-responders. Those who agreed to participate and received the questionnaire but did not complete it within the two-week period were also considered non-responders.

Commented [VR16]: ,to ensure that their answers reflect their own current understanding.

Commented [VR17]: which

Programme organizers were contacted by telephone to arrange an interview. An information and consent form were sent in advance. The interview guide consisted of demographic details and semi-structured questions addressing the barriers to organizing programmes in special schools. Those who did not respond to an invitation to interview were considered non-responders.

Data analysis

Responses to the online questionnaire were downloaded and transferred to an Excel spreadsheet (Microsoft, 2019). Summary statistics for participant demographics and MCQ responses were calculated in Excel. Any responses in open text boxes were categorized under common subthemes and listed as summary statistics in the tables. These responses were presented as themes created rather than verbatim responses. Interviews were audio recorded and transcribed for thematic analysis; the codes were created using inductive method. For the thematic analysis, each response was familiarized, and codes were created for emergent themes (as described by Braun and Clark).(23) These themes were further categorized under common category of themes for the interpretation as barriers and suggestions.(23)

RESULTS

Questionnaire responses of special school managers

We identified 67 special schools, among whom, only 39 had a valid contact detail and could be contacted. One was public and 38 were private schools. Although all 39 schools agreed to participate, only 13/39 (33.0%) went on to complete the questionnaire within the time allowed. (Table 1).

Commented [VR18]: This represents only 19% of SES in the study area. This is a study limitation

Table 1: Demographic details of school management participants

Demographics	**Median (n=13)**	**Inter Quartile Range**
Age	40	16.5 (35, 51.5)
Sex	Total number of participants n=13 (%)	
Male	4 (31)	
Female	9 (69)	
Speciality of schools	Total number of schools n=13 (%)	
Multiple disability	10 (77)	
Autism	2 (15)	
Intellectual disability	1 (8)	
	Median (n=13)	Inter Quartile Range
Number of children in schools	36	26 (14,40)

Knowledge

Overall, 92.3% (n=12) of respondents were aware of the number of children with eye conditions in their schools and 76.9% (n=10) were aware that early treatment could result in better visual outcomes. However, only 23.1% (n=3) had any knowledge that children with SEN have increased risk of developing eye conditions. Sixty three percent (n=9) said they identified vision problems by just observing the behaviour of the child in classrooms.

The main emergent themes in the open-ended text box responses with regards to symptoms of reduced vision faced by the children in the classroom was *"Difficulty in reading and writing, difficulty in copying from blackboard"* which was reported by 53.8% (n=7) of participants, followed by *"Poor hand eye coordination"* which was reported by 30.8% (n=4). Another major theme that emerged was *"Difficulty in sports activities"* which was reported by 30.8% (n=4). Most participants said lack of nutrition (92.3%, n=12) and hereditary predisposition (76.9%, n=10) were the main reasons for children developing eye problems and 30.7% (n=4) felt an eye check-up should be considered only if necessary. The most well-known condition was squint (69.2%, n=9) and the least well-known was glaucoma (7.7%, n=1) (Table 2).

Table 2: Participant response to knowledge related questions

Commented [VR19]: Respective schools

Deleted: K

Table 2	
**Participant responses for knowledge related questions**	
**Knowledge**	**n=13 (%)**
**Are you aware of the number of children with eye conditions in your school? (MCQ type)**	
Yes	12 (92.3%)
Not sure	1(7.7%)
**How likely are children with SEN to develop eye problems? (MCQ type)**	
Very likely	3 (23.1%)
Same as children without disability	3 (23.1%)
Less likely	2 (15.4%)
Not sure	5 (38.5%)
**Among the following eye conditions, which are the most common conditions that could affect children with SEN? (MCQ type)**	
Squint	9 (69.2%)
Big eyeballs	4 (30.7%)
Refractive error	3 (23.1%)
Cataract	3 (23.1%)
Small eyeballs	3 (23.1%)
Eye cancer	2 (15.4%
Conjunctivitis	2 (15.4%)
Glaucoma	1 (7.7%)
**What do you think is/are the reason/s for children with SEN developing eye problems (MCQ type)**	
Heredity	10 (76.9)
lack of Nutrition	12 (92.3)
Physical illness	9 (69.2)
Injury	9 (69.2)
Consanguinity	7 (53.9)
Curse from God	2 (15.4)
**What are the symptoms that could indicate a child of having vision problem? (Open ended)**	
Difficulty in reading and writing	7 (53.8)
Difficulty in sports activities	4 (30.8)
Difficulty in mobility	1 (7.7)
Difficulty in eye hand coordination	4 (30.8)
Difficulty in copying from blackboard	7 (53.8)
Difficulty in identifying objects around	3 (23.1)
Others (avoid using computer, headache, neck pain, and low attention span)	4 (30.8)
Not sure	3 (23.1)

Commented [VR20]: The table already has a Title, why is this repeated?

Commented [VR21]: Remove blank rows

Commented [VR22]: Lack

Commented [VR23]: is

How often is an eye check-up warranted for children with SEN? (MCQ type)	
Twice a year	5 (38.5%)
Once a year	4 (30.8%)
Only if needed	4 (30.8%)

Attitude

Most participants (92.3%; n=12) felt that children with vision problems were less capable of performing academic activities, although 69.2% (n=9) felt that they would be able to participate in extracurricular activities. Over half of the participants (61.5%; n=8) felt that children with VI or blindness, without any other additional disability, should attend normal schools as this could improve motivation and self-confidence. While 92.3% (n=12) said disabled children can interact with non-disabled peers, 30.8% (n=4) felt that they should not be attending normal school as they require special attention on a regular basis and need for additional teaching materials and methods.

To the question asked in open text boxes about why/why not children with VI should be attending mainstream schools, one major theme that emerged was *"Inclusive schools."* A third of participants (30.8%; n=4) felt that children with VI should attend regular schools if they are inclusive, while 15% (n=2) said they should attend normal school if these schools provided Braille training. A participant stated, "*The teachers need to pay more attention while teaching these children, which is not practically possible in regular schools*," while another participant said, "*They should go to an inclusive school, they deserve to be with normal children*" (Table 3).

Table 3: Participants response to attitude related questions

Table 3	
**Participant responses for attitude related questions**	
**Attitude**	**n = 13 (%)**
**Do you think children with SEN can perform academic tasks? (Open ended)**	
Yes, they can perform academic tasks	1 (7.7%)

Commented [VR24]: open-ended questions

Commented [VR25]: on possible reasons why children should/should not be…..

Commented [VR26]: these seem more like conditions needed for children to attend regular schools as opposed to "reasons WHY they should/should not attend regular schools. Please review the responses carefully and rephrase.

Commented [VR27]: In the methods you mention that you did not use "verbatim responses". Please rephrase that

Commented [VR28]: Remove as stated above

No, they cannot perform academic tasks	12 (92.3%)
**Do you think children with SEN are capable to participate in non-academic activities such as sports or extracurricular activities? (Open ended)**	
Yes, they can participate and perform	9 (69.2%)
Yes, but not fully capable	1 (7.7%)
Not sure	3 (23.1%)
**Can children with vision impairment attend mainstream school? (Open ended)**	
Yes	8 (61.5%)
No	4 (30.8%)
Not sure	1 (7.7%)
**Do you think children with vision impairment can interact with normal peers? (Open ended)**	
Can interact normally	12 (92.3%)
Did not answer	1 (7.7%)

Commented [VR29]: of participating

Practice

Close to half of the participants (46.2%; n=6) said that they would refer children who face difficulty in academic tasks such as reading and writing to an eye doctor, but 46.2% (n=6) were unsure of what to do or gave generic responses, for e.g. "*We never faced such issues in our school" or "I would counsel the parents*". However, 53.8% (n=7) participants said they make the children sit closer to the blackboard or the instructor, if they had difficulty in copying from the blackboard. One participant said," *I would encourage the child to use his/her spectacles*" while another said, "*We allow the child to use prescribed low vision device when needed."* Forty six percent (n=6) of participants said they had never organized an eye health programme in their schools in the past, while one participant said, "*We approached few doctors for eye camps, but they asked us to bring the children to their hospital"*.

Commented [VR30]: of participants

When asked about availability of accessible facilities in their schools, 53.8% (n=7) participants stated that they have additional trained caretaker staff, while one participant stated, *"We do not have any child with low vision in our school"*. Thirty nine percent (n=5) of

Commented [VR31]: of participants…

the schools provided a nutritious diet in their schools. Twenty three percent (n=3) participants stated that they provided a mixer of nutritious diet including pulses, millet, finger millet, fruits, green leafy vegetables as salads, chicken meat, boiled eggs, and milk in their schools while few stated that they did not provide any food in their schools. However, two participants stated that they check the lunch box and give feedback/suggestions to the parents to include nutritious diet (Table 3).

Commented [VR32]: of participants

Commented [VR33]: a nutritious

Table 4: Participants response to practice related questions

Table 4	
**Participant responses for practice related questions**	
**Practice**	**N=13 (%)**
**What would you do if you see a child keeping books close to his/her face while reading and writing? (Open ended)**	
Refer to eye doctor	6 (46.2%)
Modify working distance	3 (23.1%)
Help in copying and writing	1 (7.7%)
Did not answer	1 (7.7%)
Others (Child may have near vision problem, we will test his/her vision)	2 (15.4%)
**What classroom adaptations are made for vision impaired children in your school? (Open ended)**	
Encourage them to use prescribed optical devices	2 (15.4)
Ask the children to sit closer to the blackboard or the teacher	7 (53.9)
Provide large font and bright coloured materials	3 (23.1)
Braille training	2 (15.4)
Provide extra time to complete the task	2 (15.4)
Did not answer	2 (15.4)
**What would you do If you suspect a child to have eye problem? (Open ended)**	
Refer to eye doctor	7 (53.9%)
Provide special care	1 (7.7%)
Help them wear glasses	1 (7.7%)
Ask them to stay home until they get better	2 (15.4%)
Did not answer	2 (15.4%)
**What would you do If a child has an untreatable eye condition? (Open ended)**	
Create adaptive environments	4 (30.8%)
Refer them to doctor/rehabilitation	4 (30.8%)
Not sure	3 (23.1%)
Generic responses (Counsel parents, Train the child to live independently, we have not faced such problems yet)	3 (23.1%)
**How often are eye health programs organised in your schools? (MCQ)**	
Once a year	3 (23.1%)
Twice a year	1 (7.7%)

Commented [VR34]: remove

Deleted: are

Rarely	2 (15.4%)
Never	6 (46.2%)
Did not answer	1 (7.7%)
**What are the facilities available for children in your schools with regards to accessibility? (Open ended)**	
Extra trained caretaker staff	7 (53.8%)
Railings, ramps, and support handles	2 (15.4%)
Comfortable seating	1 (7.7%)
None	2 (15.4%)
Did not answer	1 (7.7%)
**Do you provide nutritious diet for children in your school? (Open ended)**	
No such provisions at the school	6 (46.2%)
Suggest parents to include nutritious diet	2 (15.4%)
Provide nutritious diet at school	5 (38.5%)
None	2 (15.4%)
Did not answer	1 (7.7%)

Interview responses of eye health programme organizers

Of four known community outreach organizers from four different organizations in Hyderabad, two responded and agreed to be interviewed. One was a public health specialist with more than 2 decades of experience and the other, an optometrist and head of community ophthalmology services with a decade of experience in the field. Interview transcripts were coded for thematic analysis. The three major themes were a) barriers to organizing SEH programs, b) existing good practice, and c) suggestions to improve services. Under each theme, sub-themes were coded and categorized.

Commented [VR35]: were they from private or public organizatios

Barriers

Low priority

Most of the special schools covered by their respective organizations were run by the government and were mostly located in urban areas. One organizer covered 1000 schools every year of which only 0.5% were special schools, the second organizer covered 50 schools in a year of which less than 2% were special schools. The reasons for the low priority given for including special schools in their programmes are described as follows: Firstly, both interviewees said that the reason for the exclusion of special schools in their

programmes was that they assumed but not sure that an eye examination was carried out as part of an annual general health check-up conducted in special schools. The second reason for the low coverage of special schools was that the time taken for completing a SEH programme in one special school was much longer compared to the time taken for one mainstream school which is even more challenging due to the limited time availability to complete their programmes within the school calendar due to frequent public holidays, school vacations and exam periods. Hence special schools were not prioritized.

Programme Initiative or lack of ownership

Special SEH programmes are usually done as stand-alone programmes and hence they are rarely included in government or NGO sponsored activities. Government sponsored programmes are not usually inclusive of special schools and most programmes run by both governmental and NGOs are focused on the number of children screened rather than needs-based activity. Hence, the initiative from these organizations is limited. On the other hand, special schools seldom take the initiative in organizing eye health programmes. Overall, there is a lack of motivation from all the stakeholders concerned. One interviewee said, "*Very rarely we receive requests from one or two private schools to conduct eye health programmes in their schools, but never from special schools*."

A lack of stakeholder engagement

Special schoolteachers are already occupied with various responsibilities pertaining to other governmental/non-governmental activities. Therefore, they are unwilling or lack motivation to take up additional responsibilities. Without schoolteachers' involvement it is impossible to examine children with SEN, which is another reason for the organizers not considering special schools in their programmes. In some situations, the teachers or the staff are often transferred to a different school or unavailable, which makes it difficult to convince the new management or teachers to help in organizing programmes in these schools. Unlike mainstream schools, special SEH programmes require the parents to attend while the eye

Commented [VR36]: respective

Commented [VR37]: , but were not sure,

Commented [VR38]: Sentence is too long…re-phrase

Commented [VR39]: This merely covers "lack of initiative". What does "lack of ownership mean as it is not covered in the paragraph below?

Commented [VR40]: being a needs…

Commented [VR41]: This paragraph appears to highlight "stakeholder related challenges"

Commented [VR42]: reported reason for….

Commented [VR43]: are unavailable..

examination is being carried out. However, it is difficult to coordinate parents' availability for all the children on the day of the programme. Hence, there is limited opportunity to educate and create awareness among the parents about their children's eye condition and needs.

Skilled human resource shortages

Unlike mainstream schools, special schools require skilled workforce who are trained in the assessment of children with SEN, low vision assessment (LVA), prescription of spectacles and low vision devices (LVDs). Most SEH programmes are conducted by community eye health workers, schoolteachers and vision technicians who are not trained to carry out a complete eye examination. Involving a more skilled workforce, i.e., optometrists or ophthalmologists, would incur additional costs. In some circumstances, such cadres are unwilling to participate in the SEH programmes due to long-distance travel and limited financial benefits. Therefore, most special SEH programmes are conducted by optometry trainees unsupervised.

Commented [VR44]: to travel

Impossible to task-shift to non-specialists

Commented [VR45]: optometrists are not "specialists". These skills you refer to are part of "undergraduate competencies and not "specialist" competencies.

Training of schoolteachers, community eye health workers (CEHW) and administrative staff in screening of children with SEN is not possible as it requires more time and expertise in the field. One interviewee said, *"I can understand the difficulties in assessing these children owing to the training that I received during my low vision assessment training, which was part of my undergraduate degree, but not everyone can understand. So, I think having a basic training in assessing these children helps in organizing such programmes."* Children with SEN require additional tests for vision assessment and refraction which cannot be performed by school staff and CEHW. Additionally, there is a limited scope for training children in usage of spectacles or LVDs due to the unavailability of parents, time, and training environment.

Commented [VR46]: Do you mean opportunity?

Additional clinical equipment and assessment needs

A complete eye examination for children with SEN requires expensive equipment, hence increased demand for financial resources. Secondly, only dry refraction can be performed on the school premises and children require a referral for cycloplegic refraction. Poor referral uptake among this group makes it difficult to provide effective refractive error correction via SEH programmes alone.

Poor availability and affordability of spectacles and low vision devices

Interviewees said that the low-cost spectacles which are commonly provided through SEH programmes are not of good quality, hence there is a higher chance of breakage and discomfort to children. However, providing good quality spectacles requires increased financial resources which is a major challenge. Additionally, unavailability and unaffordability of LVDs pose a major challenge. Most of the time these devices are just prescribed and not provided. It is highly likely that the parents do not purchase them. One organizer said, *"Although governmental provisions are available for purchasing LVDs, these are available only on paper and not accessible in reality."*

Good practices and suggestions

The two themes of existing good practice in special schools and organizations and suggestions for improved service provision are summarized in Table 4.

Commented [VR47]: Where programmes are being conducted,

**Table 4: List of existing good practices and points to be considered in the future programmes**
**Good practices followed in special schools**
• Medical history record keeping for every child – Helps in understanding the overall medical conditions of children
**Good practices followed by eye care providers**
• Helping the parents understand the eye health needs of their children during eye examination
• Creating awareness among the schoolteachers about the need for eye care services for children with SEN
• Mandatory training of screening team members prior to the programme in examining children with SEN
**Suggestions: Points to be considered prior to organizing SEH programmes in special schools**
• Making sure that the programme venues are accessible

• Inform parents/guardians well in advance and encourage them to be available during the screening
• Develop a standard operating protocol pertaining to special school eye health programmes
• Stakeholder involvement should include local NGOs and volunteers
• Facilitate the availability of good quality spectacles and LVDs
• Government should consider monetary or non-monetary incentives for participating NGOs/organizations

One organizer mentioned, *"Many of these special schools maintain health records for each child, which is helpful for us to understand the overall health of the child. It would be good if every school followed this habit."* There are several good practices that were mentioned by the organizers, to have been followed by the screening teams themselves. One organizer said, *"We make sure that the parents understand the condition of their child's eye and it is also easy for us to create awareness, if the parents are present during examination."*

One organizer mentioned, *"Before every school screening program in special schools, the parents must be informed and encouraged to be present during the program. This will also improve the child's cooperation level."* Another organizer said, *"If government could give some financial or other incentives to the volunteering NGOs, they will be motivated to participate in special school screening programs."*

One organizer said, "We ourselves find it difficult to procure low vision devices if needed as these are not easily available and also very expensive."

DISCUSSION

Universal eye health and the Sustainable Development Goal 3 "to ensure healthy lives for all people at all ages" cannot be achieved without ensuring equitable access to eye health care for underserved groups.(24) This is the first study in India to report on special school managers' knowledge, attitudes, and practices about the eye health needs of children with SEN. Overall, the findings point to a varied level of understanding, however, heads of special schools can be catalysts for change, in their own schools and among their peers; existing

Commented [VR48]: Split into 2 sentences

evidence suggests increased eye health knowledge leads to better health-seeking behaviour among parents, school managements.(18, 19)

Commented [VR49]: and

Encouragingly, most participants said they were aware of the number of children in their schools with eye conditions and that early intervention could result in better eye health outcomes. However, this claim could only be validated by identifying the true numbers by carrying out a prevalence survey in these schools. Additionally, if this is the case, then educating the staff to encourage/remind the parents to take their children for regular eye check-up would enable continuum of care for children with eye conditions. This is especially important if there are no eye health programs organized in schools. Even though most participants knew that children with SEN needed regular eye check-up, less than a third of schools reported to have conducted eye health programmes in the past, which is lower than the 53% reported in a similar study from Wales, UK in 2014.(13)

Commented [VR50]: or vision assessments

Although, refractive error is one of the most common conditions reported in children with SEN,(25, 26), only less than a quarter of participants were aware of this condition. This is alarming as uncorrected refractive error could lead to avoidable VI which would further amplify any difficulties caused by a pre-existing disability. Conversely, if corrected in time, it could improve the quality of life for these children.(27-29) Most participants said poor nutrition could cause eye problems, but none of the participants mentioned vitamin A deficiency (VAD). Perhaps including VAD specifically in the questionnaire MCQs would have impacted the responses on the causes reported. Few studies have reported VAD among children with SEN.(25, 30) This also warrants active inclusion of health promotion as part of special SEH programs in the future.

Commented [VR51]: add actual percentage

Commented [VR52]: Noting the importance of vision and ocular health, perhaps….

Commented [VR53]: Delete

Suggestion- ..warrants more careful selection of questions in future studies and inclusion of health promotion….

Most participants exhibited a negative attitude towards the ability of vision-impaired children to perform academic tasks. This could negatively impact eye health-seeking behaviour or referral practices among school staff. In many circumstances, management of a child's overall disability status takes precedence over eye health-related issues, thereby affecting the eye health seeking behaviour among the stakeholder groups.(31) Almost two

Deleted: and

thirds of the participants thought children with only vision impairment could study in mainstream schools assuming that these schools are inclusive. Although positive impact of inclusive education on psychosocial well-being is supported by studies from high-income countries (32), the evidence in LMICs is inadequate.(33) Additionally, this study did not explore to understand this factor.

Practices such as providing non-optical assistive devices, Braille printed reading materials, and encouraging children to use their spectacles and prescribed low vision devices are encouraging and should be included in health education programs. The varied level (mostly low) of knowledge about the eye health needs of children with SEN among SSM in Hyderabad is comparable to reports from mainstream primary schoolteachers outside India,(17, 18, 34). However, it does not correlate with mainstream primary schools in India.(16, 19), which could be due to higher coverage of mainstream schools by SEH programmes in India compared to special schools. This might have had a positive impact on the awareness level among mainstream schoolteachers which was also reported in China and Vietnam.(35, 36) Alternatively, this disparity could also be due to the inclusion of school heads in this study rather than school/class teachers.

Although it was reported that the regular health check-ups in the schools included eye health, this could not be verified in this study. Hence, future research should prioritise assessing the frequency and ophthalmic content of general health check-ups at special schools, as well the competency levels of those who perform these examinations. Should check-ups be found to be insufficient to fully meet the needs of children with SEN, both schools and programme organisers will have a greater incentive to prioritise inclusion of special schools in broader programme activities. From the schools' point of view, it is also possible that overall disability takes priority over eye health, and hence the schools also do not take initiative in organizing SEH programmes. This also shows a possible knowledge gap among the school management.

Commented [VR54]: ..to further understand

Deleted: h

Deleted: T

Deleted: s

Deleted: , which

Deleted: side

The barriers reported for implementing programmes in special schools included a lack of ownership, skilled human resources, difficulty in task-shifting, unaffordability, and availability of LVDs. A systematic review in 2018, reporting on the interventions to improve school-based eye care in LMICs also reported similar findings.(37) Training of skilled eye health workers such as optometrists in assessing children with SEN, prescription of glasses and LVDs in children and incentivizing them for attending special school eye health programmes may help in addressing the issue of unavailability of human resource problems.(38) Additionally, facilitating availability of LVDs and making them accessible and affordable for these children are some of the aspects that need to be worked out at policy makers' level. Furthermore, increasing eye health awareness among special school managers, schoolteachers and parents would help in better referral uptake of services and improve continuum of care.(31, 38) Hence, there is a need for appropriate referral set ups in their proximity for the referred children to avail these services.

Commented [VR55]: This does not accurately reflect the challenges that teachers face as you have reported in that section. Reconsider the phrase "lack of ownership"

Commented [VR56]: human resource shortages

Commented [VR57]: facilities

Lack of stakeholder (teachers/parents/guardians) engagement in SEH programme was another major concern reported. Lack of motivation among teachers was reported on the assumption that they are already overburdened with other health programmes and responsibilities, a scenario also reported in Pakistan.(39) Perhaps incentivizing the teachers or staff for taking up such responsibilities might improve this situation.(38) The organizers reported that there is a limited opportunity to conduct eye health awareness sessions for the primary stakeholders (parents/guardians), as most of them do not attend the screening programmes. This may also have a significant impact on the continuum of care due to poor referral uptake.(31)

Commented [VR58]: this was not tested. Rather, you had raised their legitimate challenges

Commented [VR59]: Thoughts to reflect on….
What type of incentives? Should this not be an integral part of the role of teachers. If financial incentives… how cost effective and sustainable will that be?

Commented [VR60]: What is your recommendation to rectify this challenge?

The organizers also made several recommendations to improve KAP of stakeholders and to increase uptake of eye health programmes in special schools. They suggested that incorporating health promotion strategies within SEH programmes could make them more impactful and might indirectly address the issue of sustainability.(31) When the stakeholders are better informed about the eye health needs of these children, it would positively impact

their eye health seeking behaviour, similar to studies in mainstream schools from China and Vietnam.(35, 36) Additionally, financial subsidiaries or free of charge spectacles/LVDs could also reduce the burden on these families who might otherwise be facing increased health care costs associated with caring for children with SEN.(40)

Limitations

This study should be interpreted in the context of its limitations. Firstly, the participants recruited were the school heads from a minority of special schools in the Hyderabad area and the response rate was also very low. Hence, the participants of these institutions may not be representative of all headteachers/schools in the area. Secondly, the study period was interrupted by COVID-19 travel restrictions, meaning face-to-face sensitization and enrolment of headteachers was not possible. Thirdly, although the questionnaire was piloted among the peers of the principal investigator, it was not validated for the target audience, which might have resulted in varying levels of understanding between participants. Additionally, it is difficult to ascertain whether the participants used any external resources to answer the questionnaire, which might have impacted the results.

Furthermore, the day-to-day engagement of the headteachers with the children in their schools was unverifiable and it might have been valuable to have recruited class teachers for more insight into the daily activities and challenges faced by the children. However, this was hampered by the pandemic which posed difficulty in contacting the class teachers. Lastly, the interviews were conducted over phone call and hence the further exploration of the responses to the semi-structured questions was not done. Further exploration would have highlighted several important components which were otherwise not captured in this study.

Conclusion

This study highlights a potential gap in the knowledge, attitude and practice of special school managers and barriers to organize SEH programmes in special schools in India. Measuring

Commented [VR61]: Only 19.4% of SES are represented in this study which may limit generalizability of findings

Commented [VR62]: And exclusion of other pertinent questions

Deleted: , h

Commented [VR63]: telephonically

the eye health awareness level and KAP among schoolteachers in a larger sample would give a better insight into the matter. It further demands inclusion of eye health promotion strategies to create eye health awareness among all the stakeholder groups. This study also highlights barriers to organize SEH programmes in special schools that included accessibility and affordability of eye health services. Thus there is a need to conduct a situational analysis to estimate the prevalence of VI and other eye conditions to understand the current need and service gap for children with SEN.

Commented [VR64]: ….comprehensive situational analysis or vision screening programme…..

Acknowledgements

The authors would like to acknowledge the help received from Ms.Mahalaxmi Mojjada for translating the consent form and questionnaire into Telugu language. The authors also would like to thank all the participants for their valuable time responding to the questionnaire and the interview.

Financial support

This study was carried out as part of MSc thesis at London School of Hygiene and Tropical Medicine (LSHTM), Keppel Street, London, UK. During the study period the principal investigator (WP) was supported by British Council for Prevention of Blindness, LSHTM, and Commonwealth eye health consortium. Other authors (PM, IM, RCK) did not receive any financial or in-kid incentives to be a part of this study. The funders had no role in the study design, data collection and analysis, decision to publish, or preparation of the manuscript.

Competing interest statement

The authors have declared no competing interests exists.

References

1.Das M, Spowart K, Crossley S, Dutton GN. Evidence that children with special needs all require visual assessment. Arch Dis Child. 2010;95(11):888-92.

2.Salt A, Sargent J. Common visual problems in children with disability. Arch Dis Child. 2014;99(12):1163-8.

3.Disabled Persons in India a statistical profile 2016. Ministry of Health and family welfare; 2016.

4.Andersen ES, Dunlea A, Kekelis LS. Blind children's language: resolving some differences. J Child Lang. 1984;11(3):645-64.

5.Hatton DD, Bailey DB, Burchinal MR, Ferrell KA. Developmental Growth Curves of Preschool Children with Vision Impairments. Child Development. 1997;68(5):788-806.

6.Molloy A, Rowe FJ. Manneristic Behaviors of Visually Impaired Children. Strabismus. 2011;19(3):77-84.

7.Kempen GI, Ballemans J, Ranchor AV, van Rens GH, Zijlstra GA. The impact of low vision on activities of daily living, symptoms of depression, feelings of anxiety and social support in community-living older adults seeking vision rehabilitation services. Quality of life research : an international journal of quality of life aspects of treatment, care and rehabilitation. 2012;21(8):1405-11.

8.Kulkarni SK, Veer AB, Gaikwad A, Deshpande M. The impact of early intervention on a child with vision loss in infancy - A parental perspective. Indian journal of ophthalmology. 2022;70(9):3377-81.

9.Education for children with disabilities- improving access and quality , UKAID, Department of International Development https://assets.publishing.service.gov.uk/government/uploads/system/uploads/attachment_data/file/67664/edu-chi-disabil-guid-note.pdf.

10.Frick KD, Riva-Clement L, Shankar MB. Screening for refractive error and fitting with spectacles in rural and urban India: cost-effectiveness. Ophthalmic epidemiology. 2009;16(6):378-87.

11.Lester BA. Comparing the cost-effectiveness of school eye screening versus a primary eye care model to provide refractive error services for children in India. Community Eye Health. 2007;20(61):15.

12.Saravanan S, Krishnamurthy S, Ramani KK, Narayanan A. Cost Analysis of a Comprehensive School Eye Screening Model from India. Ophthalmic epidemiology. 2022:1-7.

13.Woodhouse JM, Davies N, McAvinchey A, Ryan B. Ocular and visual status among children in special schools in Wales: the burden of unrecognised visual impairment. Arch Dis Child. 2014;99(6):500-4.

14.Donaldson LA, Karas M, O'Brien D, Woodhouse JM. Findings from an opt-in eye examination service in English special schools. Is vision screening effective for this population? PLoS One. 2019;14(3):e0212733.

15.Allen LC, Dillon A, Bowen P. Eye Care for Children in Special Schools: An Audit of Provision. The British and Irish orthoptic journal. 2021;17(1):27-32.

16.Ambika K, Nair N. A Study on Awareness of Primary School Teachers Regarding Refractive Errors and its Early Identification among Primary School Children. International Journal of Nursing Education. 2013;5:6-9.

17.Habiba U, Ormsby GM, Butt ZA, Afghani T, Asif M. Knowledge and practices of teachers associated with eye health of primary school children in Rawalpindi, Pakistan. Taiwan J Ophthalmol. 2017;7(1):28-33.

18.Maru A, Belete G, Adimassu N. Knowledge, attitude and associated factors among primary school teachers regarding refractive error in school children in Gondar city, Northwest Ethiopia. PLOS ONE. 2018;13:e0191199.

19.Thomas A. A comparative study to assess the knowledge of primary school teachers regardingrefractive errors among primary school children in selected urban and rural schools, Mangalore: RajivGandhi University of Health Science. 2013.

20.Cassetti V, Sanders T, Bruce A. Challenges of Eye Health Care in Children and Strategies to Improve Treatment Uptake: A Qualitative Study from the Perspective of Eye Care Professionals in the UK. British and Irish Orthoptic Journal. 2019.

21.Paudel P, Yen PT, Kovai V, Naduvilath T, Ho SM, Giap NV, et al. Effect of school eye health promotion on children's eye health literacy in Vietnam. Health Promotion International. 2017;34(1):113-22.

22.Archana Vishwakarma PG, Rupsha Mitra, Sajid Ali. Teachers' perspective, awareness and understaning of Student's eye health, Tech Mahindra Foundation, New Delhi. 2021.

23.Braun V, Clarke V. Using thematic analysis in psychology. Qualitative Research in Psychology. 2006;3:77-101.

24.The Sustainable Development Goals Report 2023: Special Edition: United Nations; 2023 [Available from: https://unstats.un.org/sdgs/report/2023/The-Sustainable-Development-Goals-Report-2023.pdf.

25.Gogate P, Soneji FR, Kharat J, Dulera H, Deshpande M, Gilbert C. Ocular disorders in children with learning disabilities in special education schools of Pune, India. Indian journal of ophthalmology. 2011;59(3):223-8.

26.Pehere NK, Khanna RC, Marlapati R, Sannapaneni K. Prevalence of ophthalmic disorders among hearing-impaired school children in Guntur district of Andhra Pradesh. Indian journal of ophthalmology. 2019;67(4):530-5.

27.Alvarez-Peregrina C, Sánchez-Tena M, Andreu-Vázquez C, Villa-Collar C. Visual Health and Academic Performance in School-Aged Children. Int J Environ Res Public Health. 2020;17(7).

28.Burton MJ, Ramke J, Marques AP, Bourne RRA, Congdon N, Jones I, et al. The Lancet Global Health Commission on Global Eye Health: vision beyond 2020. The Lancet Global health. 2021;9(4):e489-e551.

29.Jan C, Li SM, Kang MT, Liu L, Li H, Jin L, et al. Association of visual acuity with educational outcomes: a prospective cohort study. The British journal of ophthalmology. 2019;103(11):1666-71.

30.Wen J, Yang T, Zhu J, Guo M, Lai X, Tang T, et al. Vitamin a deficiency and sleep disturbances related to autism symptoms in children with autism spectrum disorder: a cross-sectional study. BMC pediatrics. 2021;21(1):299.

31.Cassetti V, Sanders T, Bruce A. Challenges of Eye Health Care in Children and Strategies to Improve Treatment Uptake: A Qualitative Study from the Perspective of Eye Care Professionals in the UK. The British and Irish orthoptic journal. 2019;15(1):96-104.

32.Price R. Inclusive and special education approaches in Developing countries: https://assets.publishing.service.gov.uk/media/5c6ac403ed915d4a39787401/373_Inclusive_and_Special_Education_Approaches.pdf. 2018.

33.Hannah Kuper AS, Howard White. Rapid Evidence Assessment (REA) of What Works to Improve Educational Outcomes for People with Disabilities in Low- and Middle-Income Countries. International Centre for Evidence in Disability, London School of Hygiene and Campbell Collaboration. 2018.

34.Okoloagu N, Okoye O, Onwubiko S, Eze C, Eze B, Chuka-Okosa C. A Survey of Teachers’ Knowledge, Attitudes, and Practices Related to Pupils’ Eye Health and School-Based Eye-Health Services. Nigerian Journal of Ophthalmology. 2019;27(2):68-75.

35.Liu S-M, Chang F-C, Chen C-Y, Shih S-F, Meng B, Ng E, et al. Effects of Parental Involvement in a Preschool-Based Eye Health Intervention Regarding Children's Screen Use in China. International Journal of Environmental Research and Public Health. 2021;18(21):11330.

36.Paudel P, Yen PT, Kovai V, Naduvilath T, Ho SM, Giap NV, et al. Effect of school eye health promotion on children's eye health literacy in Vietnam. Health Promot Int. 2019;34(1):113-22.

37.Burnett AM, Yashadhana A, Lee L, Serova N, Brain D, Naidoo K. Interventions to improve school-based eye-care services in low- and middle-income countries: a systematic review. Bulletin of the World Health Organization. 2018;96(10):682-94d.

38.Zhao J, Wang H, Guan H, Du K, Zhang Y, Congdon N. Impact of Teacher Incentive Intervention on Students' Vision Healthcare Uptake: A Cluster-Randomized Controlled Trial. Int J Environ Res Public Health. 2022;19(19).

39.Bechange S, Gillani M, Jolley E, Iqbal R, Ahmed L, Bilal M, et al. School-based vision screening in Quetta, Pakistan: a qualitative study of experiences of teachers and eye care providers. BMC public health. 2021;21(1):364.

40.Malvankar-Mehta MS, Wilson R, Leci E, Hatch K, Sharan S. Cost and quality of life of overlooked eye care needs of children. Risk management and healthcare policy. 2018;11:25-33.

Figure Legend

Figure 1: Participant recruitment flow chart

Supplementary material

Participant questionnaire for the assessment of knowledge, attitude, and practice among special school managers

Response to Reviewers

Dear Reviewers,

We would like to thank each of the reviewers for their valuable, detailed review and inputs on this manuscript. It has been an excellent learning experience throughout the process of reanalysis and manuscript writing with your inputs. Please find below the responses for each of the comments and inputs from each of the reviewers. We will be very happy to clarify/modify if there are any additional changes or information that need to be additionally added to the manuscript.

Reviewer 1:

1. Title: must better reflect the study which has a KAP component and barriers and enablers to eye care.

Thank you for taking the time to review our manuscript and giving your feedback. We have revised the title to the following "A cross-sectional survey of eye health knowledge, attitude, and practice among special school managers and barriers to eye health programmes in special schools in Hyderabad, India"

2. Methodological concerns: The study is described as mixed methods, but the data is not managed or presented clearly according to requirements of each method. There needs to be clarification between the quantitative analysis and thematic analysis used in the qualitative part.

This study as stated in the manuscript was a mixed method design which included quantitative component and qualitative components. The study consisted of an online questionnaire to capture the knowledge, attitude and practice among the special school managers and an interview of eye health program organizers to understand the barriers to special school eye health programs. The questionnaire consisted of multiple-choice questions and open-ended text boxes. The participant demographics and responses for the multiple-choice questions were analysed as quantitative data for presenting summary statistics. Responses in the open-ended text format and interviews were analysed as qualitative data. The open-ended text responses were categorized under common subthemes and presented within the tables as summary statistics whereas the interview responses were coded and categorized under eight major themes and presented as thematic analysis. Additionally, the participant responses in both open-ended text as well as interviews were stated as quotes in the results section. We did not conduct any inferential statistics as the main objective of the study was not to understand any correlations.

Although, we do agree that an inferential would have added more value to this study, unfortunately we were unable to collect all the information needed to conduct an inferential statistic due to the COVID-19 imposed travel restrictions during the study period. However, we intend to study this in detail as we have secured a grant to conduct a large-scale eye health program in special schools in the study area.

The selection criteria applied must be more clearly articulated to clarify steps and sampling strategy eg. whether all managers or only one leader in each school were invited to participate etc.

Any special school in the study area with valid contact details was eligible for inclusion. Organizations such as early intervention centres, therapy centres, homes for the disabled and schools for the blind were excluded. Within schools, only headteachers, directors or principals who were directly involved in school management activities (referred to in this study as 'managers') were eligible for inclusion. Among the managers who could be contacted via the valid contact details were included; typically one manager from each organization. SEH program organizers with a minimum of two years' experience in community eye health activities from organization that are active in conducting community eye health activities in the Hyderabad region were eligible for inclusion.

The questionnaire design must be detailed. ie. the basis for inclusion of specific questions in each area of the questionnaire and how were these validated for inclusion.

The participants were emailed a link to an online questionnaire built with Google Forms. The questionnaire included some questions used in previous studies conducted in mainstream schools (references given below), plus additional questions written for this study. The questionnaire included

demographic details and questions structured to capture KAP with regards to the eye health needs of children attending special schools. It included multiple-choice questions and open-ended text boxes to allow participants to express their views.

References

1. Ambika K, Nair N. A Study on Awareness of Primary School Teachers Regarding Refractive Errors and its Early Identification among Primary School Children. International Journal of Nursing Education. 2013;5:6-9.

2. Habiba U, Ormsby GM, Butt ZA, Afghani T, Asif M. Knowledge and practices of teachers associated with eye health of primary school children in Rawalpindi, Pakistan. Taiwan J Ophthalmol. 2017;7(1):28-33.

3. Maru A, Belete G, Adimassu N. Knowledge, attitude and associated factors among primary school teachers regarding refractive error in school children in Gondar city, Northwest Ethiopia. PLOS ONE. 2018;13:e0191199.

4. Thomas A. A comparative study to assess the knowledge of primary school teachers regardingrefractive errors among primary school children in selected urban and rural schools, Mangalore: RajivGandhi University of Health Science. 2013.

5. Archana Vishwakarma PG, Rupsha Mitra, Sajid Ali. Teachers' perspective, awareness and understaning of Student's eye health, Tech Mahindra Foundation, New Delhi. 2021.

3. The Results for the quantitative analysis should include some inferential analysis e.g. were there associations between knowledge and practices, or attitudes and practices and were these significant.

We agree that conducting an inferential analysis would add significant weightage to the study and would give meaningful information on the KAP. However, this study was not designed for inferential analysis. Additionally, the number of participants recruited does not allow for meaningful tests of association or statistical significance. Therefore we have not included such results in this study.

Qualitative analysis results must be presented in more detail with the emergent Themes and the specific codes per theme explained and presented as such. Quantitative data is referred to as themes, hence causing confusion.

The thematic analysis of the interview responses was coded and categorized under two major themes as Barriers and Suggestions. Under these major themes, subcategories were explained in detail (The subcategories as follows: program coverage, Program initiative, Lack of skilled human resource, Lack of stakeholder engagement, Impossible to task shift to non-specialists, Poor availability and accessibility of spectacles and low vision devices)

The questionnaire responses were listed as summary tables as explained above in response to question 2. We have modified these as per the suggestions.

4. The Discussion will be strengthened if the methods and results are sorted out.

We have made several changes to the discussion section as per the re-analysis of the data and the results. Pertaining to both the questionnaire and interview responses.

For e.g: From the questionnaire point of view we have added the following points “Almost all participants said poor nutrition could cause eye problems, but none of the participants mentioned vitamin A deficiency (VAD). Perhaps including VAD specifically in the questionnaire MCQs would have impacted the responses on the causes reported. This may also imply that many participants were unaware of the importance of vitamin A for good eye health, which might directly impact the diet provided in these schools. However, few schools where meals were provided included vitamin A rich items such as dairy, meat, egg, and green vegetables. These claims cannot be directly attributed to their knowledge about the nutrition pertaining to good eye health but overall health. Although there are few studies reporting VAD in children with SEN, one study reported low vitamin A levels among Autism spectral disorders from China in 2021. However, specific knowledge on the role of nutrition and eye

health among this group is seems to be limited. This also warrants active inclusion of health promotion as part of special SEH programs in the future.

Apart from creating adaptive environments in schools, many participants said they provided non-optical assistive devices such as large print reading materials, bright coloured objects, and tactile learning materials such as Braille printed reading materials. In addition, they also mentioned that they would encourage the children to use their spectacles and allow them to use prescribed low vision devices. Such practices are encouraging and should be included in health education programs

From the interviews point of view the following statements have been amended and added to the discussion: For e.g. The SEH programme organizers reported that the inclusion of an eye examination in annual general health check-ups was one of the main reasons for the low coverage of special schools in SEH programmes. However, this was not reported by the SSM staff. This misalignment could be a crucial component of any service coverage gap. Future research should prioritise assessing the frequency and ophthalmic content of general health check-ups at special schools, as well the competency of the personnel doing them to reliably assess the eye health and vision of children with SEN. Should check-ups be found to be insufficient to fully meet the needs of children with SEN, both schools and programme organisers will have a greater incentive to prioritise inclusion of special schools in broader programme activities.

Lack of stakeholder (teachers/parents/guardians) engagement in SEH programme was another major concern reported. Lack of motivation among teachers was reported on the assumption that they are already overburdened with other health programmes and responsibilities, a scenario also reported in Pakistan. The organizers reported that there is a limited opportunity to conduct eye health awareness sessions for the primary stakeholders (parents/guardians), as most of them do not attend the screening programmes. This may also have a significant impact on the continuum of care due to poor referral uptake.

5. The Conclusion should highlight findings in relation to the key study objectives and recommendations.

We have added the following as the conclusion as per suggestion, “This study highlights a potential gap in the knowledge, attitude and practice of special school managers and barriers to organize SEH programmes in special schools in India. Measuring the eye health awareness level and KAP among schoolteachers in a larger sample would give a better insight into the matter. It further demands inclusion of eye health promotion strategies to create eye health awareness among all the stakeholder groups. This study also highlights barriers to organize SEH programmes in special schools that included accessibility and affordability of eye health services. Thus there is a need to conduct a situational analysis to estimate the prevalence of VI and other eye conditions to understand the current need and service gap for children with SEN.”

6. There are minor language edits needed as tracked in the manuscript.

We have attempted to improve the writing style throughout the manuscript as part of this revision.

Reviewer 2

Main comments:
Many thanks for a thought-provoking article. Establishing a connection between the responses from the two groups of participants, namely teachers and School Eye Health (SEH) organizers, would provide valuable insights. Exploring teachers' perspectives on the SEH organizers' list of barriers and enablers could shed light on their perceptions and potential collaboration challenges. Notably, the organizers suggest that all children should undergo either annual or admission eye checks. It is essential to examine whether this recommendation is implemented in practice. If the organizers assert that SEH programs are unnecessary because eye care already takes place, it raises the question of whether this belief constitutes the primary barrier to effective eye care, particularly if annual checks or checks on admission or not occurring for everyone. In light of these considerations, the research could benefit from focusing less on theoretical assessments of the perceived importance of eye care and instead prioritize investigating the current state of eye care in India. Questions that address the percentage of children who have received comprehensive eye examinations, the timing of their most

recent tests, the prevalence of spectacle usage or eye conditions among the children, and a comparison with existing studies would enhance the understanding of the current situation.

Thank you very much for your review and valuable suggestions for future research in this area. We agree that an important next step is to report on the status of children's eye examinations, eye health and spectacle use. Due to the COVID-19 restrictions, studying the current state of eye care in special schools was not possible. Reaching out to the schoolteachers rather than the managers was also not possible. We have secured a grant to conduct a large-scale school eye health program in special schools in this region. As part of this project, we intend to collect data on the status of availability of services and compare the results with the level of knowledge of schoolteachers. We would also like to understand any correlation in the vision impairment and refractive errors, prevalence of spectacle usage and the awareness levels. We also would be able to study the spectacle compliance among these children.

Introduction:

1. School eye health (SEH) programs have the potential to provide children with access to good quality services and can be cost-effective (9-11). It would be helpful to specify which services are included in SEH programs.

The sentence has been amended as following: School eye health (SEH) programmes have a potential to provide children with access to high quality eye care services including refractive error correction and additional referral services and it can be cost-effective.

2. In a study by Woodhouse et al. in 2014 (12), it was found that only 53% of special schools in Wales, UK, had conducted eye health programs. It would be beneficial to include data from India on this topic, or mention if no data is available.

There are no such studies or reports available from Indian context and we have included the same in the manuscript as well.

3. In the introduction, it would be valuable to compare the prevalence of eye problems in children with autism, children with multiple disabilities, and children with no disabilities. Additional information and references from Donaldson papers and the Seeability website could be included.

We have added several study references to the introduction section (References 1, 9-22) on various disabilities which highlight the need for eye care interventions in among children with autism, intellectual impairment, hearing impairment and cerebral palsy both within and outside India.

4. The reader needs to know what currently happens in India and in each school included in the research. It would be relevant to mention whether each child has received an eye examination or screening test, and what those tests consisted of. Details about vision tests, cover tests, ocular motility, BV tests, cycloplegia, refraction, and fundus and media examinations would be informative. Additionally, it would be helpful to clarify the procedures followed if a child is unable to respond to a letter test, such as using pictures or preferential looking tests, or referring the child to a hospital.

We acknowledge the importance of including these elements in the data collection process. Although our original aim was to conduct a cross sectional study in a sample of these schools in India, COVID-19 restrictions at the time of the study disrupted the proposed examination of children. This is also the main reason for difficulty in contacting the schools and the teachers online as the principal investigator was in the UK at the time of the study. We will certainly consider these components to be added in our future research in this area.

5. Special school managements- Is this Special school managers/head teachers?

This included headteachers, directors and principals. Everyone had a different title although they hada similar roles in their schools which is the role of a head teacher.

6. Please check SEH written in full in the first instance.

This has been corrected as per the suggestion

7. Sometimes SHE used. This has been corrected as per the suggestion

8. Throughout the paper, it should be explicitly addressed that the study assesses the knowledge and attitudes of school managers and the barriers and enablers of organizers to SEH. This should be reflected in the title as well.

We have modified the title as per the suggestion as follows “A cross-sectional survey of eye health knowledge, attitude, and practice among special school managers and barriers to eye health programmes in special schools in Hyderabad, India”

Methodology:
9. In Table 1, it may be more effective to present the 13 schools separately rather than together.

Thank you for the suggestion. While it is a very good idea to do so, we presented it as a small table as the other tables are very large and may not be appealing to the reader. However, we can modify this if the reviewer feels it would add more value.

10. Table 1 specify years for age.

Included as suggested

11. Instead of focusing on theoretical scenarios, it could be more realistic to ask schools about the eye care provided to the children currently under their care. A theoretical methodology is likely to lead to positive subjective bias.

We agree with this point. We will consider this in our future research which we will be conducting for the special schoolteachers in the study area. We welcome any suggestions towards the new methodology, and it will be valuable for us for the future research in the area as we have received a grant to pursue research in this area.

12. The number of school managers who are aware of children with an eye condition does not mean that they have identified all the children with eye conditions if a full sight test not carried out on all children.

This is true. We also believe that unless an eye examination is carried out in these schools it will be difficult to ascertain this merely based on the response from these participants. This we believe will be addressed in the future research.

13. Stating that health programmes are warranted twice a year does not mean they occur, particularly for all children. Many people are under the impression that some children are too disabled for a sight test. What is included in a health programme?

This is very true. However, the aim of this question was to understand what these participants felt it to be appropriate, not to understand how many times a year they had conducted in the past. We did not ask the question pertaining to the annual health check-up. As the questionnaire responses were conducted prior to the interviews. Perhaps conducting the organizer interviews before creating the

questionnaire would have helped in framing the questions related to past health/eye health programs in the schools.

14. It would be valuable to determine the percentage of children who have received an eye test or screening program. Which type was it, how long ago it occurred, and the average age at the first test.

Thank you for the suggestion. We will consider this in our future research.

15. The study should also inquire if each child has a documented sight test in their clinical records and if there is a management plan for any diagnosed conditions.

Thank you for the suggestion. We will consider this in our future research.

16. Comparing the number of visually impaired children, those wearing refractive correction, or diagnosed with eye conditions to other populations of children with special educational needs (SEN) would provide useful insights. If significantly lower numbers are found, it suggests that schools are not identifying all cases.

This is the next step in our future research. Conducting eye health programs in the special schools and identifying the eye health awareness levels among schoolteachers. Thank you for reiterating these important points.

17. It would be beneficial to inquire if carers understand the implications for each child diagnosed with an eye condition and if they felt they required further education. For example, clarification could be sought regarding whether a child with freely alternating manifest strabismus would be classified as visually impaired and require magnification or if it simply affects their coordination.
18. It would be pertinent to ask how often each child's glasses should be checked and what the procedure is in case of breakage.

Thank you very much for your helpful suggestions in points 11-18 for future research building on this initial study of knowledge, attitudes, and practice among schoolteachers.

19. Online questionnaire should be included.

Thank you for this suggestion. We have included the questionnaire as supplementary material.

Results:
20. It is unclear why there is a discrepancy in the responses of participants 1 and 2 regarding the frequency of eye check-ups (yearly or on admission). Clarification should be sought from all schools to confirm if children receive an annual eye health assessment or one on admission, as stated by the health organizers. Additionally, it would be important to determine if any children fall through the gaps and do not receive an eye test.

As mentioned in question 13, the questionnaire responses were conducted prior to the interviews. Perhaps conducting the organizer interviews before creating the questionnaire would have helped in framing the questions related to past health/eye health programs in the schools. Also we feel that the different responses from two participants could be due to difference in the level of priority that each of their organizations might be having to conduct school eye health programs each year.

21. It would be relevant to explore if schools are aware that they can request an SEH program, as stated by the organizers.
22. The schools' perspectives on the barriers stated by the organizers should be included. Do they feel unwilling to participate?
23. It should be clarified if the schools are allowed to refer children to a doctor and, if so, which type of

doctor.
24. Environmental modifications or adaptations mentioned in the results should be explained.
25. The SEH organizers could be asked if the identified barriers impact the child's annual or admission eye check.
26. If the organizers believe that SEH is unnecessary because eye care already occurs, it raises the question of why they are concerned about barriers to SEH.

This is a very valid point. Thank you for highlighting this point. This must also be included in the future research questionnaire item.

Discussion:

27. When children have difficulties with reading, writing, and multiple disabilities, it can be challenging for caregivers to determine whether the issues stem from eye problems, IQ problems, autism, or other factors related to their disabilities. It would be helpful to address whether all children struggling are referred to eye specialist even if carers are unsure what the problem is.

28. The SEH organizers should be checked whether they have verified if each child receives their annual or admission eye check-up, or if they assume that it happens.

Thank you very much for your helpful suggestions in points 20-28 for future research building on this initial study.

Reviewer 3

This is an interesting and useful contribution to understanding the barriers to service provision in special schools.

Thank you very much for your review and feedback.

My comments:

1) Line 81-84. In the introduction the authors refer to eye care programs in Wales. There is now a wider England study that is driving the debate that I think is worth considering.
Findings from an opt-in eye examination service in English special schools. Is vision screening effective for this population? L. A. Donaldson, M. Karas, D. O'Brien and J. M. Woodhouse. PloS one 2019 Vol. 14 Issue 3 Pages e0212733
I would also like to see this idea explored more as to the relevance or not of this single example of a program in the UK.

Thank you for you suggestion. We have included the above citation as well we found another relevant study. These both studies highlight different sides of the problems 1) Donaldson LA et al presents the provision for providing eye care for children with SEN among the eye care providers whereas 2) Woodhouse et al, presents the problem from the special schools' point of view as how often they conducted eye health programs in schools. We have highlighted these studies and elaborated in the introduction section.

2) The study defines itself as a mixed methods study (line 98-99) but it is hard to work out how the data was handled? It talks of a thematic analysis, and I am assuming this is of both the open text parts of the online questionnaires and the recorded interviews. (Line 148-148). This needs to be clarified. It then goes onto to say the questionnaire data themes were determined but says nothing of the interview thematic analysis which you would expect to lead to some other themes.

In the results section it then discusses results under each of these three themes defined in the methods section, but this data seems to be only the results of knowledge, practice, and attitude multiple choice questions. I cannot see any discussion of the results of the thematic analysis of the free text responses.

This study as stated in the manuscript was a mixed method design which included quantitative component and qualitative components. The study consisted of an online questionnaire to capture the knowledge, attitude and practice among the special school managers and an interview of eye health program organizers to understand the barriers to special school eye health programs. The questionnaire consisted of multiple-choice questions and open-ended text boxes. The participant demographics and responses for the multiple-choice questions were analysed as quantitative data for presenting summary statistics. Responses in the open-ended text format and interviews were analysed as qualitative data. The open-ended text responses were categorized under common subthemes and presented within the tables as summary statistics. Additionally, the participant responses in both open-ended text as well as interviews were stated as quotes in the results section.

The thematic analysis of the interview responses was coded and categorized under two major themes as Barriers and Suggestions. Under these major themes, subcategories were explained in detail (The subcategories as follows: program coverage, Program initiative, Lack of skilled human resource, Lack of stakeholder engagement, Impossible to task shift to non-specialists, Poor availability and accessibility of spectacles and low vision devices)

3) The results of the interviews do offer quotes as would be expected of a thematic analysis and the data does seem to be grouped into headings but there is no mention made of themes generated form an analysis of this data.

We have included quotes as suggested from both questionnaire as well as interviews

4) There is no discussion of the methodology of the thematic analysis. How was it planned, how was the data coded and how was a thematic framework arrived at. There is also no mention of the theoretical underpinnings of the approach to the thematic analysis. This is a complex subject, but I feel if the authors a claiming a thematic methodology they should make some effort to describe within the bounds of accepted thinking on this method. (Braun & Clarke, 2006; Kiger & Varpio, 2020). This does not need to be detailed but it does need a mention.

Thank you for these resources. We have followed the methods suggested by Braun and Clarke for the analysis. We have also included this in the methodology section as reference.

Response to the reviewers

Dear Reviewers, thank you for your taking time to review our article and for your valuable comments.

Reviewer 4

It seems that the reviewer 4 has given inputs based on the first version of the manuscript rather than the revised version. However, the inputs and views are well taken, and the authors thank the reviewer for their time and suggestions.

The manuscript is well written with a few typos

Comment 1: The sample size of 33% was found to be too low, indicating a low uptake.

Response: The low response rate we think is due to the impact of COVID-19 pandemic during the study period. The uptake was very low despite multiple attempts to contact and encourage the participants to complete the survey.

Comment 2: The small size is too low for the results to be generalized

Response: We completely agree with this comment and therefore we have added this point to the limitation section

Comment 3: 2/4 invited eye health programme organizers, although they form 50% of the population an effort could have been made to interview online all 4 health programme organizers

Response: All the interviewers were contacted via individual telephone calls. Two organizers could not be reached through phone calls, and they were also unresponsive for the email invitations. This study was conducted in the middle of the COVID pandemic, and the investigators were in London, UK and hence it was also challenging to contact them through alternate contact details.

Reviewer 5:

Comment 1: This is an interesting study and worth reporting. The manuscript is very well written with least grammatical errors. However, some of the underlisted limitations are ones authors could have corrected easily before commencing the actual data collection. Example is especially ensuring the questionnaire used was reliable and valid to measure exactly what it seeks to measure. I think authors instead of recommending another study to report on a comprehensive situational analysis, should have taken some time to collect data and analyze these findings also to enrich the current report further.

Response: Thank you for your comments and inputs. While we totally agree with the point that the questionnaire used was reliable and valid prior to the study, this was a challenge due to some unavoidable circumstances. Although the original study was designed to capture the awareness levels by conducting interviews face to face, this was not possible due to the COVID-19 travel restrictions, the investigators were in

London, UK, and very limited time frame to carry out the study as this was part of a MSc public health program project submission. Therefore, questionnaires were developed within a limited timeframe and carried out online. However, the questionnaire was pilot tested among peers of the principal investigator prior to the administration. We do agree that it would have been better to have had similar participant demographics for the pilot, the opportunity was limited to reach a uniform audience. Additionally, there are no preexisting questionnaire reported in the literature to identify the awareness level among the special school managers. Hence this would be the first study to report on this. The experience and findings from this study has also helped us understand this lacuna and a need for a validated questionnaire to measure the awareness levels in special schools.

Comment 2: Line 39-40 on the old document; Lines 41 -42 of revised document: Is not grammatically correct to begin a sentence with a number or fraction like this. I suggest authors modify this sentence. E.g. A number of thirteen (13) out of sixty-seven (67) invited SSM (19%) participated.

Response: Thank you for the suggestion. We have modified it as suggested

Comment 3: Line 51 on the old document; Lines 53-54 of revised doc: I think current work should have considered also conducting a comprehensive situational analysis of these findings so discussion would be rich. This is just a scanty result.

Response: We completely agree with this comment, however, there was no scope for a situational analysis due to the pandemic restrictions. Hence this could not be done.

Comment 4: Lines 128-130 in old document; Lines 146 – 148 on the revised doc: How are authors sure responders' answers reflect their current understanding. What precautions or measures did author put in place to ensure responders to not refer to other resources on the internet especially as questionnaire stayed with them for two weeks. Please indicate this.

Response: This was one of the limitations of the study. Although the participants were instructed not refer to other resources, one could not have known whether they did so. The participants were encouraged during the phone call not to refer to other resources and about the negative impacts of them referring to other resources on the results. The participants were also explained that there were no wrong answers, rather they must express what they already knew as it were. However, the authors do understand this is not 100% fool proof method. This point has been indicated in the limitations section.

Comment 5: Line 154-155 in old document; Line 172 -173 on revised document: Demographic data like age and gender distributions are repeated in tables and in the text. It will be advisable for authors to delete from the text and to simply refer to the table 1 to avoid repetitions.

Response: We have modified the document as suggested

Comment 6: Line 292 in old document; Line 445 -447 I revised document: The questionnaire used should have at least been tested for its reliability. If these was

done in the pilot study, please indicate how reliable this tool was. With use of an unreliable questionnaire, how are we sure of the reliability of the results?

Response: Kindly refer to the response to comment 1

Reviewer #6: General comments

I appreciate the author's efforts to explore barriers to eye health service provision in special schools in Hyderabad India. The study investigates the “Knowledge, attitude and practice” of special school headteachers regarding various aspects of eye health among children in their schools. Further, the study also reports from interviews conducted among managers of two organisations involved in school eye health programs in the area.
The findings from the study could be of interest to those working in this field, particularly for those involved in program planning and management in the local area. The authors have attempted to address the comments raised by the reviewers in the first round.

Response: Dear Reviewer thank you for taking time in going through the manuscript and for your comments and valuable inputs. This was a great learning experience.

The manuscript in its current form, however, still has several concerns and limitations. As previous reviewers raised, there is significant concern about the validity and comprehensiveness of the survey questionnaire used. Further, the limited sample size (both quantitative and qualitative parts) also hinders any generalisation of the findings. The language and formatting of the manuscript also need improvement for clarity, conciseness, and consistency.
I suggest that the authors undertake a thorough revision and subsequently resubmit the work for further consideration.

Specific comments:
1. Title:
a. Considering the length of the title, the study design could be removed (as the study not only included “a cross-sectional survey” but also interviews).
b. Also consider clarifying that the barriers relate to organising the eye health programs (not accessing). For example, “…. and barriers to organising eye health programs…”

Response: We have modified this as suggested “Eye health knowledge, attitude, and practice among special school managers and barriers to eye health programmes in special schools in Hyderabad, India”

Abstract:
1. Background: the aims could be revised for clarity and ease of reading.

Response: Children with special education needs (SEN) are at higher risk of developing vision problems; however, in India, there is no data available on the awareness level among special school managers (SSM) about the eye health needs of children with SEN and barriers for providing eye care for these in children in schools. This study is aimed to identify the awareness level among SSM and barriers for organizing School Eye Health (SEH) programmes in special schools as reported by the eye health program organizers.

2. Methods:
a. Were the questions focused on “visual needs” or “eye health” as in the title?

Response: These terminologies are used interchangeably here. But we use the term Eye health very widely throughout the manuscript as we aim not just the vision but also other eye health issues. Hence we have made it uniform throughout with “ Eye health” instead of visual needs.

3. Results:
a. Line 43-45: needs to be revised for clarity – Modified as suggested
b. Line 45-46: “Only 23% of special schools organized yearly SEH programmes.” But an additional 8% (1) school organised it twice a year. So, 31% organized at least once a year? Response: Modified as suggested, this change has also been changed in the main manuscript section
c. I suggest the authors be consistent with how the barriers are reported here and in the main results section.
4. Conclusion:
a. Consistency in the use of “visual needs”, “eye health” or “eye health needs” as the focus of the study.
b. I suggest the authors be consistent with how the barriers are reported here and in the main results/discussion/conclusion section.

Response: We have made Eye health needs as the focus across the manuscript as suggested

Introduction:
1. Line 70-71: “SEN are at a higher risk of developing VI…”. This suggests that being in a SEN is causing VI (as diabetics have a higher risk of vision impairment). Or is it that these children have a higher prevalence of vision impairment and ocular comorbidity?

Response: This is a very valid question. Both the risk as well as prevalence of VI are higher among this group. We found literature reporting on higher prevalence of VI as well as the increased risk among this population (presenting odds ratio). Therefore, we have mentioned this as risk. We intend to keep this as increased risk as presence of disability itself increases the risk as most of these children cannot express their vision related difficulties unless being intentionally screened for it. Risks for developing VI such as uncorrected RE and other ocular comorbidities such as cataract, optic atrophy, degenerative and atrophic changes in the brain would

result in VI and hence these are risk factors in this case. Hence, we mentioned this as risk factors.

2. Add a description of what school eye health programs are in general and in the local context (India/Hyderabad).

Response: Although we would like to add some information on this, the word limit suggested by the journal would not allow to further elaborate on the school eye health programs in India. In India, the school eye health programs vary from organization to organization. Most organizations conduct vision screening programs rather than a comprehensive eye health program. Vision screening programs are usually carried out by trained schoolteachers or community health workers wherein, the children undergo a vision test and those who fail the cut off 6/12 or 6/9 are further referred to a primary or secondary care centres nearby. On the other hand some individual school screening programs, carry out a comprehensive eye examination, however, cycloplegic refraction is not done in the school premises due to lack of expert human resource such as ophthalmologist on site. Additionally lack of trained optometrists is another reason why there are more vision screening programs rather than comprehensive programs. This is also captured in this study.

3. Line 98: Should add the affiliation of this person

Response: The participant requested not to enclose the organization name. Therefore, we do not want to disclose it in the manuscript.

4. Line 99-108: The rationale for focusing on KAP of school managers (rather than the teachers or carers who are directly involved in the daily care of the children) needs to be clearly and explicitly described.

Response: This has been explained in the methodology as well as in the limitation section. The teachers were not included in the study as there was no scope of contacting them due to COVID-19 pandemic. The study investigators at the time were in London, UK and the face-to-face data collection was not possible. The school contact details were retrieved from an online resource which provided the details of the managers/principals/directors rather than the teachers. We also believe that including teachers would have been of great value.

Methods:

1. Line 126-129: How were these organisations identified? Are the investigators confident that all organisations were identified?

Response: There are only 4 major eye health providing organization which provide community eye health including school eye health programs of which 1 is run by the government and the 3 are run by non-governmental organizations. There are other eye health providing organizations which are corporate hospitals which do not participate in community eye health programs. The details of these organizations were gathered from one of the study participant organizations.

2. Line 137: What is the rationale for including an experience limit for the inclusion of the SHE programs organizers?

Response: The reason for this criterion was with the assumption that minimum of 2 years would have enabled an organizer to have conducted/organizer adequate number of school eye health programs to comment on the barriers.

3. Line 163-166: the revised data analysis segment provides additional information on the 'thematic analysis' citing Braun and Clark 2006 in response to the previous reviewer's comments. I am just wondering if the authors used any software to manage the data, followed all the steps as outlined by Braun and Clarke (2006), were the codes inductively generated or any theoretical frameworks used, and if there was an iterative refining and defining of the themes. Reporting on the investigator's roles and backgrounds in this process and providing the above details will help strengthen the qualitative component (adding reflexivity and credibility) of the study

Response: We did not use any software such as NVIVO as these were small set of responses hence the themes were created based on inductive method. Once the interviews were transcribed into texts, using inductive method, the codes were identified and grouped under common emerging themes. Therefore there were no theory driven method used in this study. We have added this part to the analysis section too. The investigator who conducted the interview over telephone call also transcribed the data into texts and carried out the further analysis.

Results:
1. Mention that the sample in this study represents 13 of 67 schools (19%)

Response: Although there were 67 special schools, we were able to contact only 39 of them at the end as only these schools had a valid contact detail. Out of 39 schools we received response from only 13 schools, hence we have considered this as 13/39 (33%). In this case, wouldn't this be 13/39, rather than 13/67? Could the reviewer kindly clarify why this should be the case if it should be 13/67? Thank you.

2. Information in Table 1 is largely provided in the text above, so it could be removed to reduce the length of the paper.

Response: Removed the text as suggested

3. For open-ended questions, clearly mention that the responses are the author's codes/subthemes and not the verbatim responses of the participants. It also needs to be clarified that some participants' responses may have been coded to more than one code/subtheme.

Response: Yes, these were authors codes rather than verbatim responses and we have mentioned this in the methods as suggested.

4. The results presented in Lines 184-190 do not correspond exactly to those in Table 2 as they are not reported consistently (text shows a theme of Difficulties faced in performing academic tasks" including two rows of the table. The same goes for the rest of the text/table rows. The themes and codes/subthemes within each theme need to be reported explicitly and consistently in both text and table.

Response: This has been modified uniformly in the texts as in the table.

a. It would be important if any participants were not aware of the symptoms of vision problems, but this information is hard to make from the data presented.

Response: We believe, in the knowledge part of the data, we asked about what symptoms would be noticeable in children with vision problems gives this information.

5. The methods section mentions categorising themes as good/poor knowledge, positive/negative attitude, and acceptable/non-acceptable practices. This is not reported in the results section.

a. I also if all the responses or subthemes were categorised into at least one category. For example, in Table 4, how were the responses to the question "What would you do If you suspect a child to have eye problem? (Open ended)" categorised into acceptable or non-acceptable practices?

Response: It does make sense and hence we have changed this description as the following in the methods section, "Any responses in open text boxes were categorized under common subthemes and listed as summary statistics in the tables. These responses were presented as themes created rather than verbatim responses. Interviews were audio recorded and transcribed for thematic analysis; the codes were created using inductive method."

6. Rather than lengthy paragraphs discussing multiple concepts/questions, please use paragraph breaks.

Response: Thanks for the suggestion, we have used paragraph breaks in the longer paragraph sections

7. Table 2: "How often is eye check-up warranted for children SENs". What would be the rationale for putting this question as an MCQ and not an open-ended question? And what is the rationale for the limited options presented (for example, not including a 6-monthly option)? The same goes for the rationale for selecting specific MCQs for other questions as well.

Response: The rationale for this question is to understand the participants knowledge on how frequently an eye examination is warranted for children with SENs. Ideally, it is recommended at least once every year is recommended for all

children and those with refractive error it is twice a year which is to imply 6 months. Hence this was added.

8. Line 207: "To the question asked in open text boxes about why/why not children with VI should be attending mainstream schools, …" Was that the question asked?

Response: The original question asked was, "Do you think children who are vision impaired (low vision/blind) should attend normal school? if Yes, Why/ if no, Why? "

9. Normal school vs regular school: do they mean the same thing?

Response: Yes, they both mean the same thing

10. Table 4:
a. Check the total number in the right column. I can understand there may be multiple codes/subthemes emerging from the response of one participant (and this needs to be explicitly mentioned) but this still does not explain why some questions only have 12 responses (including "did not answer" and "others").

b. For the rows with "others" and just 1 (8%), please spell out what that response was.

Response: Corrected the numbers and added the responses to the "Others" rows as well, as suggested.

11. Interview responses
a. I was surprised that there were only "four known community outreach organizations and more than that, I was disappointed that participation from all of them was not secured.

Response: There are only 4 known eye health organizations in the study area (1 public and 3 private NGOs) which provide community screening programs. Therefore, only these were included. All the other organizations in the study area or smaller eye clinics or corporate Mult speciality hospitals which do not provide community-based eye health services including school screening programs.

Secondly, all the interviewers were contacted via individual telephone calls. Two organizers could not be reached through phone calls, and they were also unresponsive for the email invitations. Three more attempts were made to contact those who did not respond to the calls or email invitations the first time. This study was conducted in the middle of the COVID-19 pandemic, and the investigators were in London, UK and hence it was also challenging to secure an alternate contact detail and it was also not possible to meet them face-to-face.

b. Based on the SSM survey responses, about a quarter of the schools reported organising eye health programs at least once a year. If we assume this to be a representative sample from the 67 special schools in the area, about 17 schools would conduct annual eye health programs. However, the organisations included in

the interview only covered 5+1 special schools (Line 251-252). Does that mean the remaining two organisations that could not be interviewed covered most of the special schools? That would be a significant limitation of the study as the key organisations providing most of the eye health programs to special schools were missed.

Response: These numbers quoted by the participants in the interview are the ones covered by their organizations on an average. We do not know whether the other two organizations even conducted eye health programs in the special schools. Hence, this must not be left for assumptions such as "the rest were covered by other organizations". According to what we gathered in the interviews, it seems that most of them do not prioritize or include special schools in their programs which indicates a potential service gap in this area. It is very rarely special schools are included in these programs even by the participated organizations. This means that most special schools in the study area might not have been screened in the past, but only a few have been covered. Additionally, according to the responses from the SSMs, their responses do not mean that they conducted eye health programs as they mentioned, rather, it could also mean that they responded randomly, leaving significant scope for recall or information bias. We agree that this is one of the limitations of the study and this has been mentioned in the limitations section.

c. I'd encourage authors to explicitly mention or clarify at the beginning of this section (and wherever relevant) that the statements presented within the themes are as expressed by the participants. Sometimes it may be confused with the author's opinion. For example, are the following statements based on what the data (participants' responses) suggests or a fact or opinion of the authors?

i. "Special SEH programmes are usually done as stand-alone programmes and hence they are rarely included in government or NGO sponsored activities."
ii. " … special SEH programmes require the parents to attend while the eye examination is being carried out."
iii. "Most SEH programmes are conducted by community eye health workers, schoolteachers and vision technicians who are not trained to carry out a complete eye examination."

Responses: These are very good points. Yes, these responses are presented as per the themes emerged from the participants response, not the authors interpretation.

d. Reading the description of the first barrier (low program coverage) for organisers, it feels like this theme is more about low priority (which then resulted in low coverage). This is my impression reading the description, not the full data. That's why I reiterate my earlier comments about iterative refining and defining/naming of the themes and the importance of reporting on the investigator's roles in this process.

Response: This is a valid point. We have modified as suggested

e. I also note that the themes in the revised manuscript are different to the barriers listed in Box 2 of the original submission. Some of the previously mentioned barriers such as “no standard screening protocols” and “infrastructures are inaccessible” are not mentioned as barriers in the revised submission.

Response: These were not included in the revised manuscript as the data was reanalysed before submitting the revision. During the reanalysis these were not found to be part of the emergent themes. Hence, we did not include these in the revised manuscript. During the reanalysis, inaccessible facilities were found not as barriers rather these were suggested by the participants. These responses are listed in the table 4.

f. Line 267-268: “On the other hand, special schools seldom take the initiative in organizing eye health programmes.” If this is the organisers’ perception, were they probed on why they believed so?

Response: This was not probed further while this would have given a perspective on why schools did not take initiatives. Hence, we have included in the discussion part that the probable reasons for this could be other disabilities taking priorities over eye health and low awareness levels. The following has been discussed in the discussion section. “From the schools’ point of view, it is also possible that overall disability takes priority over eye health, and hence the schools also do not take initiative in organizing SEH programmes. This also shows a possible knowledge gap among the school management side.”

g. A lack of stakeholder engagement: were the organisers probed about the engagement or motivation of the school management? Considering the earlier part of the study on SSM and their potential role in organising eye health programs, it seems important to explore this aspect.

Response: Due to the limited time (3 months) frame from the conception till the end reporting of the study as part of MSc work, the data collection happened parallelly rather than first questionnaire responses and then the interviews which would have helped in including questions based on the responses from the SSM group. We also agree probing the participants would have given more information on this. This is one of the reasons why we suggested further research in which we would be able to incorporate the learnings from this study. We also have secured a grant to carry out a large-scale special school eye health program in which we intend to include these aspects while doing the data collection in the future research.

Discussion:

1. The discussion section as such is quite lengthy and needs to be a bit more concise.
2. Some of the statements in the discussion seem to better fit the results section or are repeated from the results section.

Response: Thank you for this input. While we agree that if the concise discussion would be better, we aimed to keep the discussion around the most important points, many of

which cannot be ignored and hence we would like to keep most of these aspects included.

3. Some results are introduced in the discussion without a mention in the results section. For example:

a. Line 381-382: "They also felt studying with normal peers could improve their psychosocial well-being."

Response: We re-looked at the data and have modified the statement in the discussion as follows, " Although positive impact of inclusive education on psychosocial well-being is supported by studies from high-income countries (32), the evidence in LMICs is inadequate.(33) Additionally, this study did not explore to understand this factor."

b. Line 400: "However, this was not reported by the SSM staff." SSM's responses relating to annual health check-ups covering eye examinations were not mentioned in the results.

Response: This is a very valid point and we have modified this statement after relooking at the data. "Although it was reported that the regular health check-ups in the schools included eye health, this could not be verified in this study. Hence, future research should prioritise assessing the frequency and ophthalmic content of general health check-ups at special schools, as well the competency levels of those who perform these examinations."

4. Line 354-357: Does this mean that the children haven't had any eye examinations in past? It is important to have an eye examination, but it does not need to be through a school eye health program.

Response: We felt that if the respondents knew that an eye examination is important, they would have mentioned about the referring the children or conducting vision screening in a bit more detail. However, none of them mentioned anything about the past referrals or eye examinations in details. This aspect will be considered in the future research.

5. The discussion around Vitamin A and its deficiency seems overstretched as this was how the survey questions were designed (MCQ without Vit A option!). Further, Gogate at el. (Gogate P, Soneji FR, Kharat J, Dulera H, Deshpande M, Gilbert C. Ocular disorders in children with learning disabilities in special education schools of Pune, India. Indian J Ophthalmol. 2011 May-Jun;59(3):223-8.) shows that about 2% of bilateral vision impairment is due to vitamin A deficiency in children with learning disabilities in special education schools of Pune, India.

Response: This is a good point and we do agree it has been stretched and we have shortened the discussion part based on the suggestion as following, " Most participants said poor nutrition could cause eye problems, but none of the participants mentioned vitamin A deficiency (VAD). Perhaps including VAD specifically in the questionnaire MCQs would have impacted the responses on the causes reported. Few studies have reported VAD among children with SEN.(25, 30) This also warrants active inclusion of health promotion as part of special SEH programs in the future."

Limitations:
1. While the sample size limitation is mentioned, authors need to clearly mention its implication i.e. the generalisability of findings. Further, the small number of interviews conducted needs to be mentioned with its implications in data saturation and confidence in the completeness of the barriers unearthed.

Response: We have included these aspects into the limitation section as suggested.

Language and formatting:
Some suggestions to improve the language and formatting of the manuscript:
1. Break down the long and complex sentences with multiple concepts. For example, the first sentence in the abstract.

Response: We have made these changes as suggested throughout the manuscript. Thank you for the suggestion.
2. Minimise the use of non-standard abbreviations
3. Consistent use of decimal points (30.8% vs 31%) and terms (eye health providers vs organisers)

Response: This has been made uniform. Numbers have been made as decimals (30.8% etc) throughout the manuscript

4. Careful re-examination of the numbers and proportion reported. E.g.,
a. Line 359: "…only less than a third of participants were aware of this condition." Table 2 shows 23% which is less than a quarter of the participants.

Response: This has been corrected and reconfirmed
b. Line 362: "All participants said poor nutrition…" Table 2 shows 12 of 13 participants for this response.

Response: This has been changed to most participants.

Data Review URL

Click here to download Data Review URL
http://datadryad.org/stash/share/MP-
