# Supplementary material for: Eye health knowledge, attitude, and practice among special school managers and barriers to eye health programmes in special schools in Hyderabad, India
Source: PLOS Glob Public Health. 2024 Aug 28;4(8):e0002124. doi: 10.1371/journal.pgph.0002124 (PMC11356432; doi:10.1371/journal.pgph.0002124)
Supplement: Attachment — Submitted filename: PGPH-D-23-01037 _Response to the reviewers_06_11_2023.docx [file pgph.0002124.s003.docx]

Dear Reviewers,

We would like to thank each of the reviewers for their valuable, detailed review and inputs on this manuscript. It has been an excellent learning experience throughout the process of reanalysis and manuscript writing with your inputs. Please find below the responses for each of the comments and inputs from each of the reviewers. We will be very happy to clarify/modify if there are any additional changes or information that need to be additionally added to the manuscript.

Reviewer 1:

1.Title: must better reflect the study which has a KAP component and barriers and enablers to eye care.

Thank you for taking the time to review our manuscript and giving your feedback. We have revised the title to the following “A cross-sectional survey of eye health knowledge, attitude, and practice among special school managers and barriers to eye health programmes in special schools in Hyderabad, India”

2.Methodological concerns: The study is described as mixed methods, but the data is not managed or presented clearly according to requirements of each method. There needs to be clarification between the quantitative analysis and thematic analysis used in the qualitative part.

This study as stated in the manuscript was a mixed method design which included quantitative component and qualitative components. The study consisted of an online questionnaire to capture the knowledge, attitude and practice among the special school managers and an interview of eye health program organizers to understand the barriers to special school eye health programs. The questionnaire consisted of multiple-choice questions and open-ended text boxes. The participant demographics and responses for the multiple-choice questions were analysed as quantitative data for presenting summary statistics. Responses in the open-ended text format and interviews were analysed as qualitative data. The open-ended text responses were categorized under common subthemes and presented within the tables as summary statistics whereas the interview responses were coded and categorized under eight major themes and presented as thematic analysis. Additionally, the participant responses in both open-ended text as well as interviews were stated as quotes in the results section. We did not conduct any inferential statistics as the main objective of the study was not to understand any correlations.

Although, we do agree that an inferential would have added more value to this study, unfortunately we were unable to collect all the information needed to conduct an inferential statistic due to the COVID-19 imposed travel restrictions during the study period. However, we intend to study this in detail as we have secured a grant to conduct a large-scale eye health program in special schools in the study area.

The selection criteria applied must be more clearly articulated to clarify steps and sampling strategy eg. whether all managers or only one leader in each school were invited to participate etc.

Any special school in the study area with valid contact details was eligible for inclusion. Organizations such as early intervention centres, therapy centres, homes for the disabled and schools for the blind were excluded. Within schools, only headteachers, directors or principals who were directly involved in school management activities (referred to in this study as ‘managers’) were eligible for inclusion. Among the managers who could be contacted via the valid contact details were included; typically one manager from each organization. SEH program organizers with a minimum of two years’ experience in community eye health activities from organization that are active in conducting community eye health activities in the Hyderabad region were eligible for inclusion.

The questionnaire design must be detailed. ie. the basis for inclusion of specific questions in each area of the questionnaire and how were these validated for inclusion.

The participants were emailed a link to an online questionnaire built with Google Forms. The questionnaire included some questions used in previous studies conducted in mainstream schools (references given below), plus additional questions written for this study. The questionnaire included demographic details and questions structured to capture KAP with regards to the eye health needs of children attending special schools. It included multiple-choice questions and open-ended text boxes to allow participants to express their views.

References

1.Ambika K, Nair N. A Study on Awareness of Primary School Teachers Regarding Refractive Errors and its Early Identification among Primary School Children. International Journal of Nursing Education. 2013;5:6-9.

2.Habiba U, Ormsby GM, Butt ZA, Afghani T, Asif M. Knowledge and practices of teachers associated with eye health of primary school children in Rawalpindi, Pakistan. Taiwan J Ophthalmol. 2017;7(1):28-33.

3.Maru A, Belete G, Adimassu N. Knowledge, attitude and associated factors among primary school teachers regarding refractive error in school children in Gondar city, Northwest Ethiopia. PLOS ONE. 2018;13:e0191199.

4.Thomas A. A comparative study to assess the knowledge of primary school teachers regardingrefractive errors among primary school children in selected urban and rural schools, Mangalore: RajivGandhi University of Health Science. 2013.

5.Archana Vishwakarma PG, Rupsha Mitra, Sajid Ali. Teachers' perspective, awareness and understaning of Student's eye health, Tech Mahindra Foundation, New Delhi. 2021.

3.The Results for the quantitative analysis should include some inferential analysis e.g. were there associations between knowledge and practices, or attitudes and practices and were these significant.

We agree that conducting an inferential analysis would add significant weightage to the study and would give meaningful information on the KAP. However, this study was not designed for inferential analysis. Additionally, the number of participants recruited does not allow for meaningful tests of association or statistical significance. Therefore we have not included such results in this study.

Qualitative analysis results must be presented in more detail with the emergent Themes and the specific codes per theme explained and presented as such. Quantitative data is referred to as themes, hence causing confusion.

The thematic analysis of the interview responses was coded and categorized under two major themes as Barriers and Suggestions. Under these major themes, subcategories were explained in detail (The subcategories as follows: program coverage, Program initiative, Lack of skilled human resource, Lack of stakeholder engagement, Impossible to task shift to non-specialists, Poor availability and accessibility of spectacles and low vision devices)

The questionnaire responses were listed as summary tables as explained above in response to question 2. We have modified these as per the suggestions.

4.The Discussion will be strengthened if the methods and results are sorted out.

We have made several changes to the discussion section as per the re-analysis of the data and the results. Pertaining to both the questionnaire and interview responses.

For e.g: From the questionnaire point of view we have added the following points “Almost all participants said poor nutrition could cause eye problems, but none of the participants mentioned vitamin A deficiency (VAD). Perhaps including VAD specifically in the questionnaire MCQs would have impacted the responses on the causes reported. This may also imply that many participants were unaware of the importance of vitamin A for good eye health, which might directly impact the diet provided in these schools. However, few schools where meals were provided included vitamin A rich items such as dairy, meat, egg, and green vegetables. These claims cannot be directly attributed to their knowledge about the nutrition pertaining to good eye health but overall health. Although there are few studies reporting VAD in children with SEN, one study reported low vitamin A levels among Autism spectral disorders from China in 2021. However, specific knowledge on the role of nutrition and eye health among this group is seems to be limited. This also warrants active inclusion of health promotion as part of special SEH programs in the future.

Apart from creating adaptive environments in schools, many participants said they provided non-optical assistive devices such as large print reading materials, bright coloured objects, and tactile learning materials such as Braille printed reading materials. In addition, they also mentioned that they would encourage the children to use their spectacles and allow them to use prescribed low vision devices. Such practices are encouraging and should be included in health education programs

From the interviews point of view the following statements have been amended and added to the discussion: For e.g. The SEH programme organizers reported that the inclusion of an eye examination in annual general health check-ups was one of the main reasons for the low coverage of special schools in SEH programmes. However, this was not reported by the SSM staff. This misalignment could be a crucial component of any service coverage gap. Future research should prioritise assessing the frequency and ophthalmic content of general health check-ups at special schools, as well the competency of the personnel doing them to reliably assess the eye health and vision of children with SEN. Should check-ups be found to be insufficient to fully meet the needs of children with SEN, both schools and programme organisers will have a greater incentive to prioritise inclusion of special schools in broader programme activities.

Lack of stakeholder (teachers/parents/guardians) engagement in SEH programme was another major concern reported. Lack of motivation among teachers was reported on the assumption that they are already overburdened with other health programmes and responsibilities, a scenario also reported in Pakistan. The organizers reported that there is a limited opportunity to conduct eye health awareness sessions for the primary stakeholders (parents/guardians), as most of them do not attend the screening programmes. This may also have a significant impact on the continuum of care due to poor referral uptake.

5.The Conclusion should highlight findings in relation to the key study objectives and recommendations.

We have added the following as the conclusion as per suggestion, “This study highlights a potential gap in the knowledge, attitude and practice of special school managers and barriers to organize SEH programmes in special schools in India. Measuring the eye health awareness level and KAP among schoolteachers in a larger sample would give a better insight into the matter. It further demands inclusion of eye health promotion strategies to create eye health awareness among all the stakeholder groups. This study also highlights barriers to organize SEH programmes in special schools that included accessibility and affordability of eye health services. Thus there is a need to conduct a situational analysis to estimate the prevalence of VI and other eye conditions to understand the current need and service gap for children with SEN.”

6.There are minor language edits needed as tracked in the manuscript.

We have attempted to improve the writing style throughout the manuscript as part of this revision.

Reviewer 2

Main comments:
Many thanks for a thought-provoking article. Establishing a connection between the responses from the two groups of participants, namely teachers and School Eye Health (SEH) organizers, would provide valuable insights. Exploring teachers' perspectives on the SEH organizers' list of barriers and enablers could shed light on their perceptions and potential collaboration challenges. Notably, the organizers suggest that all children should undergo either annual or admission eye checks. It is essential to examine whether this recommendation is implemented in practice. If the organizers assert that SEH programs are unnecessary because eye care already takes place, it raises the question of whether this belief constitutes the primary barrier to effective eye care, particularly if annual checks or checks on admission or not occurring for everyone. In light of these considerations, the research could benefit from focusing less on theoretical assessments of the perceived importance of eye care and instead prioritize investigating the current state of eye care in India. Questions that address the percentage of children who have received comprehensive eye examinations, the timing of their most recent tests, the prevalence of spectacle usage or eye conditions among the children, and a comparison with existing studies would enhance the understanding of the current situation.

Thank you very much for your review and valuable suggestions for future research in this area. We agree that an important next step is to report on the status of children’s eye examinations, eye health and spectacle use. Due to the COVID-19 restrictions, studying the current state of eye care in special schools was not possible. Reaching out to the schoolteachers rather than the managers was also not possible. We have secured a grant to conduct a large-scale school eye health program in special schools in this region. As part of this project, we intend to collect data on the status of availability of services and compare the results with the level of knowledge of schoolteachers. We would also like to understand any correlation in the vision impairment and refractive errors, prevalence of spectacle usage and the awareness levels. We also would be able to study the spectacle compliance among these children.

Introduction:
1. School eye health (SEH) programs have the potential to provide children with access to good quality services and can be cost-effective (9-11). It would be helpful to specify which services are included in SEH programs.

The sentence has been amended as following: School eye health (SEH) programmes have a potential to provide children with access to high quality eye care services including refractive error correction and additional referral services and it can be cost-effective.

2. In a study by Woodhouse et al. in 2014 (12), it was found that only 53% of special schools in Wales, UK, had conducted eye health programs. It would be beneficial to include data from India on this topic, or mention if no data is available.

There are no such studies or reports available from Indian context and we have included the same in the manuscript as well.

3. In the introduction, it would be valuable to compare the prevalence of eye problems in children with autism, children with multiple disabilities, and children with no disabilities. Additional information and references from Donaldson papers and the Seeability website could be included.

We have added several study references to the introduction section (References 1, 9-22) on various disabilities which highlight the need for eye care interventions in among children with autism, intellectual impairment, hearing impairment and cerebral palsy both within and outside India.

4. The reader needs to know what currently happens in India and in each school included in the research. It would be relevant to mention whether each child has received an eye examination or screening test, and what those tests consisted of. Details about vision tests, cover tests, ocular motility, BV tests, cycloplegia, refraction, and fundus and media examinations would be informative. Additionally, it would be helpful to clarify the procedures followed if a child is unable to respond to a letter test, such as using pictures or preferential looking tests, or referring the child to a hospital.

We acknowledge the importance of including these elements in the data collection process. Although our original aim was to conduct a cross sectional study in a sample of these schools in India, COVID-19 restrictions at the time of the study disrupted the proposed examination of children. This is also the main reason for difficulty in contacting the schools and the teachers online as the principal investigator was in the UK at the time of the study. We will certainly consider these components to be added in our future research in this area.

5. Special school managements- Is this Special school managers/head teachers?

This included headteachers, directors and principals. Everyone had a different title although they hada similar roles in their schools which is the role of a head teacher.

6. Please check SEH written in full in the first instance.

This has been corrected as per the suggestion

7. Sometimes SHE used. This has been corrected as per the suggestion

8. Throughout the paper, it should be explicitly addressed that the study assesses the knowledge and attitudes of school managers and the barriers and enablers of organizers to SEH. This should be reflected in the title as well.

We have modified the title as per the suggestion as follows “A cross-sectional survey of eye health knowledge, attitude, and practice among special school managers and barriers to eye health programmes in special schools in Hyderabad, India”

Methodology:
9. In Table 1, it may be more effective to present the 13 schools separately rather than together.

Thank you for the suggestion. While it is a very good idea to do so, we presented it as a small table as the other tables are very large and may not be appealing to the reader. However, we can modify this if the reviewer feels it would add more value.

10. Table 1 specify years for age.

Included as suggested

11. Instead of focusing on theoretical scenarios, it could be more realistic to ask schools about the eye care provided to the children currently under their care. A theoretical methodology is likely to lead to positive subjective bias.

We agree with this point. We will consider this in our future research which we will be conducting for the special schoolteachers in the study area. We welcome any suggestions towards the new methodology, and it will be valuable for us for the future research in the area as we have received a grant to pursue research in this area.

12. The number of school managers who are aware of children with an eye condition does not mean that they have identified all the children with eye conditions if a full sight test not carried out on all children.

This is true. We also believe that unless an eye examination is carried out in these schools it will be difficult to ascertain this merely based on the response from these participants. This we believe will be addressed in the future research.

13. Stating that health programmes are warranted twice a year does not mean they occur, particularly for all children. Many people are under the impression that some children are too disabled for a sight test. What is included in a health programme?

This is very true. However, the aim of this question was to understand what these participants felt it to be appropriate, not to understand how many times a year they had conducted in the past. We did not ask the question pertaining to the annual health check-up. As the questionnaire responses were conducted prior to the interviews. Perhaps conducting the organizer interviews before creating the questionnaire would have helped in framing the questions related to past health/eye health programs in the schools.

14. It would be valuable to determine the percentage of children who have received an eye test or screening program. Which type was it, how long ago it occurred, and the average age at the first test.

Thank you for the suggestion. We will consider this in our future research.

15. The study should also inquire if each child has a documented sight test in their clinical records and if there is a management plan for any diagnosed conditions.

Thank you for the suggestion. We will consider this in our future research.

16. Comparing the number of visually impaired children, those wearing refractive correction, or diagnosed with eye conditions to other populations of children with special educational needs (SEN) would provide useful insights. If significantly lower numbers are found, it suggests that schools are not identifying all cases.

This is the next step in our future research. Conducting eye health programs in the special schools and identifying the eye health awareness levels among schoolteachers. Thank you for reiterating these important points.

17. It would be beneficial to inquire if carers understand the implications for each child diagnosed with an eye condition and if they felt they required further education. For example, clarification could be sought regarding whether a child with freely alternating manifest strabismus would be classified as visually impaired and require magnification or if it simply affects their coordination.
18. It would be pertinent to ask how often each child's glasses should be checked and what the procedure is in case of breakage.

Thank you very much for your helpful suggestions in points 11-18 for future research building on this initial study of knowledge, attitudes, and practice among schoolteachers.

19. Online questionnaire should be included.

Thank you for this suggestion. We have included the questionnaire as supplementary material.

Results:
20. It is unclear why there is a discrepancy in the responses of participants 1 and 2 regarding the frequency of eye check-ups (yearly or on admission). Clarification should be sought from all schools to confirm if children receive an annual eye health assessment or one on admission, as stated by the health organizers. Additionally, it would be important to determine if any children fall through the gaps and do not receive an eye test.

As mentioned in question 13, the questionnaire responses were conducted prior to the interviews. Perhaps conducting the organizer interviews before creating the questionnaire would have helped in framing the questions related to past health/eye health programs in the schools. Also we feel that the different responses from two participants could be due to difference in the level of priority that each of their organizations might be having to conduct school eye health programs each year.

21. It would be relevant to explore if schools are aware that they can request an SEH program, as stated by the organizers.
22. The schools' perspectives on the barriers stated by the organizers should be included. Do they feel unwilling to participate?
23. It should be clarified if the schools are allowed to refer children to a doctor and, if so, which type of doctor.
24. Environmental modifications or adaptations mentioned in the results should be explained.
25. The SEH organizers could be asked if the identified barriers impact the child's annual or admission eye check.
26. If the organizers believe that SEH is unnecessary because eye care already occurs, it raises the question of why they are concerned about barriers to SEH.

This is a very valid point. Thank you for highlighting this point. This must also be included in the future research questionnaire item.

Discussion:

27. When children have difficulties with reading, writing, and multiple disabilities, it can be challenging for caregivers to determine whether the issues stem from eye problems, IQ problems, autism, or other factors related to their disabilities. It would be helpful to address whether all children struggling are referred to eye specialist even if carers are unsure what the problem is.

28. The SEH organizers should be checked whether they have verified if each child receives their annual or admission eye check-up, or if they assume that it happens.

Thank you very much for your helpful suggestions in points 20-28 for future research building on this initial study.

Reviewer 3

This is an interesting and useful contribution to understanding the barriers to service provision in special schools.

Thank you very much for your review and feedback.

My comments:

1) Line 81-84. In the introduction the authors refer to eye care programs in Wales. There is now a wider England study that is driving the debate that I think is worth considering.
Findings from an opt-in eye examination service in English special schools. Is vision screening effective for this population? L. A. Donaldson, M. Karas, D. O’Brien and J. M. Woodhouse. PloS one 2019 Vol. 14 Issue 3 Pages e0212733
I would also like to see this idea explored more as to the relevance or not of this single example of a program in the UK.

Thank you for you suggestion. We have included the above citation as well we found another relevant study. These both studies highlight different sides of the problems 1) Donaldson LA et al presents the provision for providing eye care for children with SEN among the eye care providers whereas 2) Woodhouse et al, presents the problem from the special schools’ point of view as how often they conducted eye health programs in schools. We have highlighted these studies and elaborated in the introduction section.

2) The study defines itself as a mixed methods study (line 98-99) but it is hard to work out how the data was handled? It talks of a thematic analysis, and I am assuming this is of both the open text parts of the online questionnaires and the recorded interviews. (Line 148-148). This needs to be clarified. It then goes onto to say the questionnaire data themes were determined but says nothing of the interview thematic analysis which you would expect to lead to some other themes.
In the results section it then discusses results under each of these three themes defined in the methods section, but this data seems to be only the results of knowledge, practice, and attitude multiple choice questions. I cannot see any discussion of the results of the thematic analysis of the free text responses.

This study as stated in the manuscript was a mixed method design which included quantitative component and qualitative components. The study consisted of an online questionnaire to capture the knowledge, attitude and practice among the special school managers and an interview of eye health program organizers to understand the barriers to special school eye health programs. The questionnaire consisted of multiple-choice questions and open-ended text boxes. The participant demographics and responses for the multiple-choice questions were analysed as quantitative data for presenting summary statistics. Responses in the open-ended text format and interviews were analysed as qualitative data. The open-ended text responses were categorized under common subthemes and presented within the tables as summary statistics. Additionally, the participant responses in both open-ended text as well as interviews were stated as quotes in the results section.

The thematic analysis of the interview responses was coded and categorized under two major themes as Barriers and Suggestions. Under these major themes, subcategories were explained in detail (The subcategories as follows: program coverage, Program initiative, Lack of skilled human resource, Lack of stakeholder engagement, Impossible to task shift to non-specialists, Poor availability and accessibility of spectacles and low vision devices)

3) The results of the interviews do offer quotes as would be expected of a thematic analysis and the data does seem to be grouped into headings but there is no mention made of themes generated form an analysis of this data.

We have included quotes as suggested from both questionnaire as well as interviews

4) There is no discussion of the methodology of the thematic analysis. How was it planned, how was the data coded and how was a thematic framework arrived at. There is also no mention of the theoretical underpinnings of the approach to the thematic analysis. This is a complex subject, but I feel if the authors a claiming a thematic methodology they should make some effort to describe within the bounds of accepted thinking on this method. (Braun & Clarke, 2006; Kiger & Varpio, 2020). This does not need to be detailed but it does need a mention.

Thank you for these resources. We have followed the methods suggested by Braun and Clarke for the analysis. We have also included this in the methodology section as reference.
