# Supplementary material for: Eye health knowledge, attitude, and practice among special school managers and barriers to eye health programmes in special schools in Hyderabad, India
Source: PLOS Glob Public Health. 2024 Aug 28;4(8):e0002124. doi: 10.1371/journal.pgph.0002124 (PMC11356432; doi:10.1371/journal.pgph.0002124)
Supplement: Attachment — Submitted filename: REVIEW OF A MANUSCRIPT comments of the reviewer.docx [file pgph.0002124.s005.docx]

REVIEW OF A MANUSCRIPT

REVIEWER: DR THOKOZILE INGRID METSING

TOPIC:

A cross-sectional survey of eye health knowledge, attitude, and practice among special

school managers and barriers to eye health programmes in special schools in

Hyderabad, India

OVERVIEW OF THE REVIEW:

The manuscript is well written with a few typos

- The sample size of 33% was found to be too low, indicating a low uptake.
- The small size is too low for the results to be generalized
- 2/4 invited eye health programme organizers, although they form 50% of the population an effort could have been made to interview online all 4 health programme organizers

COMMENT:

Even though the findings of the study were found to be valuable, the study seems like it is a pilot study project.
