# Supplementary material for: Eye health knowledge, attitude, and practice among special school managers and barriers to eye health programmes in special schools in Hyderabad, India
Source: PLOS Glob Public Health. 2024 Aug 28;4(8):e0002124. doi: 10.1371/journal.pgph.0002124 (PMC11356432; doi:10.1371/journal.pgph.0002124)
Supplement: Attachment — Submitted filename: Response to the reviewers_PGPH_04_04_2024.docx [file pgph.0002124.s007.docx]

**Response to the reviewers**

Dear Reviewers, thank you for your taking time to review our article and for your valuable comments.

**Reviewer 4**

It seems that the reviewer 4 has given inputs based on the first version of the manuscript rather than the revised version. However, the inputs and views are well taken, and the authors thank the reviewer for their time and suggestions.

The manuscript is well written with a few typos

Comment 1: The sample size of 33% was found to be too low, indicating a low uptake.

Response: The low response rate we think is due to the impact of COVID-19 pandemic during the study period. The uptake was very low despite multiple attempts to contact and encourage the participants to complete the survey.

Comment 2: The small size is too low for the results to be generalized

Response: We completely agree with this comment and therefore we have added this point to the limitation section

Comment 3: 2/4 invited eye health programme organizers, although they form 50% of the population an effort could have been made to interview online all 4 health programme organizers

Response: All the interviewers were contacted via individual telephone calls. Two organizers could not be reached through phone calls, and they were also unresponsive for the email invitations. This study was conducted in the middle of the COVID pandemic, and the investigators were in London, UK and hence it was also challenging to contact them through alternate contact details.

**Reviewer 5:**

Comment 1: This is an interesting study and worth reporting. The manuscript is very well written with least grammatical errors. However, some of the underlisted limitations are ones authors could have corrected easily before commencing the actual data collection. Example is especially ensuring the questionnaire used was reliable and valid to measure exactly what it seeks to measure. I think authors instead of recommending another study to report on a comprehensive situational analysis, should have taken some time to collect data and analyze these findings also to enrich the current report further.

Response: Thank you for your comments and inputs. While we totally agree with the point that the questionnaire used was reliable and valid prior to the study, this was a challenge due to some unavoidable circumstances. Although the original study was designed to capture the awareness levels by conducting interviews face to face, this was not possible due to the COVID-19 travel restrictions, the investigators were in London, UK, and very limited time frame to carry out the study as this was part of a MSc public health program project submission. Therefore, questionnaires were developed within a limited timeframe and carried out online. However, the questionnaire was pilot tested among peers of the principal investigator prior to the administration. We do agree that it would have been better to have had similar participant demographics for the pilot, the opportunity was limited to reach a uniform audience. Additionally, there are no preexisting questionnaire reported in the literature to identify the awareness level among the special school managers. Hence this would be the first study to report on this. The experience and findings from this study has also helped us understand this lacuna and a need for a validated questionnaire to measure the awareness levels in special schools.

Comment 2: Line 39-40 on the old document; Lines 41 -42 of revised document: Is not grammatically correct to begin a sentence with a number or fraction like this. I suggest authors modify this sentence. E.g. A number of thirteen (13) out of sixty-seven (67) invited SSM (19%) participated.

Response: Thank you for the suggestion. We have modified it as suggested

Comment 3: Line 51 on the old document; Lines 53-54 of revised doc: I think current work should have considered also conducting a comprehensive situational analysis of these findings so discussion would be rich. This is just a scanty result.

Response: We completely agree with this comment, however, there was no scope for a situational analysis due to the pandemic restrictions. Hence this could not be done.

Comment 4: Lines 128-130 in old document; Lines 146 – 148 on the revised doc: How are authors sure responders’ answers reflect their current understanding. What precautions or measures did author put in place to ensure responders to not refer to other resources on the internet especially as questionnaire stayed with them for two weeks. Please indicate this.

Response: This was one of the limitations of the study. Although the participants were instructed not refer to other resources, one could not have known whether they did so. The participants were encouraged during the phone call not to refer to other resources and about the negative impacts of them referring to other resources on the results. The participants were also explained that there were no wrong answers, rather they must express what they already knew as it were. However, the authors do understand this is not 100% fool proof method. This point has been indicated in the limitations section.

Comment 5: Line 154-155 in old document; Line 172 -173 on revised document: Demographic data like age and gender distributions are repeated in tables and in the text. It will be advisable for authors to delete from the text and to simply refer to the table 1 to avoid repetitions.

Response: We have modified the document as suggested

Comment 6: Line 292 in old document; Line 445 -447 I revised document: The questionnaire used should have at least been tested for its reliability. If these was done in the pilot study, please indicate how reliable this tool was. With use of an unreliable questionnaire, how are we sure of the reliability of the results?

Response: Kindly refer to the response to comment 1

**Reviewer #6: General comments**

I appreciate the author's efforts to explore barriers to eye health service provision in special schools in Hyderabad India. The study investigates the “Knowledge, attitude and practice” of special school headteachers regarding various aspects of eye health among children in their schools. Further, the study also reports from interviews conducted among managers of two organisations involved in school eye health programs in the area.
The findings from the study could be of interest to those working in this field, particularly for those involved in program planning and management in the local area. The authors have attempted to address the comments raised by the reviewers in the first round.

Response: Dear Reviewer thank you for taking time in going through the manuscript and for your comments and valuable inputs. This was a great learning experience.

The manuscript in its current form, however, still has several concerns and limitations. As previous reviewers raised, there is significant concern about the validity and comprehensiveness of the survey questionnaire used. Further, the limited sample size (both quantitative and qualitative parts) also hinders any generalisation of the findings. The language and formatting of the manuscript also need improvement for clarity, conciseness, and consistency.
I suggest that the authors undertake a thorough revision and subsequently resubmit the work for further consideration.

Specific comments:
1. Title:
a. Considering the length of the title, the study design could be removed (as the study not only included “a cross-sectional survey” but also interviews).
b. Also consider clarifying that the barriers relate to organising the eye health programs (not accessing). For example, “…. and barriers to organising eye health programs…”

Response: We have modified this as suggested “Eye health knowledge, attitude, and practice among special school managers and barriers to eye health programmes in special schools in Hyderabad, India”

Abstract:
1. Background: the aims could be revised for clarity and ease of reading.

Response: Children with special education needs (SEN) are at higher risk of developing vision problems; however, in India, there is no data available on the awareness level among special school managers (SSM) about the eye health needs of children with SEN and barriers for providing eye care for these in children in schools. This study is aimed to identify the awareness level among SSM and barriers for organizing School Eye Health (SEH) programmes in special schools as reported by the eye health program organizers.

2. Methods:
a. Were the questions focused on “visual needs” or “eye health” as in the title?

Response: These terminologies are used interchangeably here. But we use the term Eye health very widely throughout the manuscript as we aim not just the vision but also other eye health issues. Hence we have made it uniform throughout with “ Eye health” instead of visual needs.

3. Results:
a. Line 43-45: needs to be revised for clarity – Modified as suggested
b. Line 45-46: “Only 23% of special schools organized yearly SEH programmes.” But an additional 8% (1) school organised it twice a year. So, 31% organized at least once a year? Response: Modified as suggested, this change has also been changed in the main manuscript section
c. I suggest the authors be consistent with how the barriers are reported here and in the main results section.
4. Conclusion:
a. Consistency in the use of “visual needs”, “eye health” or “eye health needs” as the focus of the study.
b. I suggest the authors be consistent with how the barriers are reported here and in the main results/discussion/conclusion section.

Response: We have made Eye health needs as the focus across the manuscript as suggested

Introduction:
1. Line 70-71: “SEN are at a higher risk of developing VI…”. This suggests that being in a SEN is causing VI (as diabetics have a higher risk of vision impairment). Or is it that these children have a higher prevalence of vision impairment and ocular comorbidity?

Response: This is a very valid question. Both the risk as well as prevalence of VI are higher among this group. We found literature reporting on higher prevalence of VI as well as the increased risk among this population (presenting odds ratio). Therefore, we have mentioned this as risk. We intend to keep this as increased risk as presence of disability itself increases the risk as most of these children cannot express their vision related difficulties unless being intentionally screened for it. Risks for developing VI such as uncorrected RE and other ocular comorbidities such as cataract, optic atrophy, degenerative and atrophic changes in the brain would result in VI and hence these are risk factors in this case. Hence, we mentioned this as risk factors.

2. Add a description of what school eye health programs are in general and in the local context (India/Hyderabad).

Response: Although we would like to add some information on this, the word limit suggested by the journal would not allow to further elaborate on the school eye health programs in India. In India, the school eye health programs vary from organization to organization. Most organizations conduct vision screening programs rather than a comprehensive eye health program. Vision screening programs are usually carried out by trained schoolteachers or community health workers wherein, the children undergo a vision test and those who fail the cut off 6/12 or 6/9 are further referred to a primary or secondary care centres nearby. On the other hand some individual school screening programs, carry out a comprehensive eye examination, however, cycloplegic refraction is not done in the school premises due to lack of expert human resource such as ophthalmologist on site. Additionally lack of trained optometrists is another reason why there are more vision screening programs rather than comprehensive programs. This is also captured in this study.

3. Line 98: Should add the affiliation of this person

Response: The participant requested not to enclose the organization name. Therefore, we do not want to disclose it in the manuscript.

4. Line 99-108: The rationale for focusing on KAP of school managers (rather than the teachers or carers who are directly involved in the daily care of the children) needs to be clearly and explicitly described.

Response: This has been explained in the methodology as well as in the limitation section. The teachers were not included in the study as there was no scope of contacting them due to COVID-19 pandemic. The study investigators at the time were in London, UK and the face-to-face data collection was not possible. The school contact details were retrieved from an online resource which provided the details of the managers/principals/directors rather than the teachers. We also believe that including teachers would have been of great value.

Methods:
1. Line 126-129: How were these organisations identified? Are the investigators confident that all organisations were identified?

Response: There are only 4 major eye health providing organization which provide community eye health including school eye health programs of which 1 is run by the government and the 3 are run by non-governmental organizations. There are other eye health providing organizations which are corporate hospitals which do not participate in community eye health programs. The details of these organizations were gathered from one of the study participant organizations.

2. Line 137: What is the rationale for including an experience limit for the inclusion of the SHE programs organizers?

Response: The reason for this criterion was with the assumption that minimum of 2 years would have enabled an organizer to have conducted/organizer adequate number of school eye health programs to comment on the barriers.

3. Line 163-166: the revised data analysis segment provides additional information on the ‘thematic analysis’ citing Braun and Clark 2006 in response to the previous reviewer’s comments. I am just wondering if the authors used any software to manage the data, followed all the steps as outlined by Braun and Clarke (2006), were the codes inductively generated or any theoretical frameworks used, and if there was an iterative refining and defining of the themes. Reporting on the investigator's roles and backgrounds in this process and providing the above details will help strengthen the qualitative component (adding reflexivity and credibility) of the study

Response: We did not use any software such as NVIVO as these were small set of responses hence the themes were created based on inductive method. Once the interviews were transcribed into texts, using inductive method, the codes were identified and grouped under common emerging themes. Therefore there were no theory driven method used in this study. We have added this part to the analysis section too. The investigator who conducted the interview over telephone call also transcribed the data into texts and carried out the further analysis.

Results:
1. Mention that the sample in this study represents 13 of 67 schools (19%)

Response: Although there were 67 special schools, we were able to contact only 39 of them at the end as only these schools had a valid contact detail. Out of 39 schools we received response from only 13 schools, hence we have considered this as 13/39 (33%). In this case, wouldn’t this be 13/39, rather than 13/67? Could the reviewer kindly clarify why this should be the case if it should be 13/67? Thank you.

2. Information in Table 1 is largely provided in the text above, so it could be removed to reduce the length of the paper.

Response: Removed the text as suggested

3. For open-ended questions, clearly mention that the responses are the author’s codes/subthemes and not the verbatim responses of the participants. It also needs to be clarified that some participants’ responses may have been coded to more than one code/subtheme.

Response: Yes, these were authors codes rather than verbatim responses and we have mentioned this in the methods as suggested.

4. The results presented in Lines 184-190 do not correspond exactly to those in Table 2 as they are not reported consistently (text shows a theme of Difficulties faced in performing academic tasks” including two rows of the table. The same goes for the rest of the text/table rows. The themes and codes/subthemes within each theme need to be reported explicitly and consistently in both text and table.

Response: This has been modified uniformly in the texts as in the table.

a. It would be important if any participants were not aware of the symptoms of vision problems, but this information is hard to make from the data presented.

Response: We believe, in the knowledge part of the data, we asked about what symptoms would be noticeable in children with vision problems gives this information.

5. The methods section mentions categorising themes as good/poor knowledge, positive/negative attitude, and acceptable/non-acceptable practices. This is not reported in the results section.

a. I also if all the responses or subthemes were categorised into at least one category. For example, in Table 4, how were the responses to the question “What would you do If you suspect a child to have eye problem? (Open ended)” categorised into acceptable or non-acceptable practices?

Response: It does make sense and hence we have changed this description as the following in the methods section, “Any responses in open text boxes were categorized under common subthemes and listed as summary statistics in the tables. These responses were presented as themes created rather than verbatim responses. Interviews were audio recorded and transcribed for thematic analysis; the codes were created using inductive method.”

6. Rather than lengthy paragraphs discussing multiple concepts/questions, please use paragraph breaks.

Response: Thanks for the suggestion, we have used paragraph breaks in the longer paragraph sections

7. Table 2: “How often is eye check-up warranted for children SENs”. What would be the rationale for putting this question as an MCQ and not an open-ended question? And what is the rationale for the limited options presented (for example, not including a 6-monthly option)? The same goes for the rationale for selecting specific MCQs for other questions as well.

Response: The rationale for this question is to understand the participants knowledge on how frequently an eye examination is warranted for children with SENs. Ideally, it is recommended at least once every year is recommended for all children and those with refractive error it is twice a year which is to imply 6 months. Hence this was added.

8. Line 207: “To the question asked in open text boxes about why/why not children with VI should be attending mainstream schools, …” Was that the question asked?

Response: The original question asked was, “Do you think children who are vision impaired (low vision/blind) should attend normal school? if Yes, Why/ if no, Why? “

9. Normal school vs regular school: do they mean the same thing?

Response: Yes, they both mean the same thing

10. Table 4:
a. Check the total number in the right column. I can understand there may be multiple codes/subthemes emerging from the response of one participant (and this needs to be explicitly mentioned) but this still does not explain why some questions only have 12 responses (including “did not answer” and “others”).

b. For the rows with “others” and just 1 (8%), please spell out what that response was.

Response: Corrected the numbers and added the responses to the “Others” rows as well, as suggested.

11. Interview responses
a. I was surprised that there were only “four known community outreach organizations and more than that, I was disappointed that participation from all of them was not secured.

Response: There are only 4 known eye health organizations in the study area (1 public and 3 private NGOs) which provide community screening programs. Therefore, only these were included. All the other organizations in the study area or smaller eye clinics or corporate Mult speciality hospitals which do not provide community-based eye health services including school screening programs.

Secondly, all the interviewers were contacted via individual telephone calls. Two organizers could not be reached through phone calls, and they were also unresponsive for the email invitations. Three more attempts were made to contact those who did not respond to the calls or email invitations the first time. This study was conducted in the middle of the COVID-19 pandemic, and the investigators were in London, UK and hence it was also challenging to secure an alternate contact detail and it was also not possible to meet them face-to-face.

b. Based on the SSM survey responses, about a quarter of the schools reported organising eye health programs at least once a year. If we assume this to be a representative sample from the 67 special schools in the area, about 17 schools would conduct annual eye health programs. However, the organisations included in the interview only covered 5+1 special schools (Line 251-252). Does that mean the remaining two organisations that could not be interviewed covered most of the special schools? That would be a significant limitation of the study as the key organisations providing most of the eye health programs to special schools were missed.

Response: These numbers quoted by the participants in the interview are the ones covered by their organizations on an average. We do not know whether the other two organizations even conducted eye health programs in the special schools. Hence, this must not be left for assumptions such as “the rest were covered by other organizations”. According to what we gathered in the interviews, it seems that most of them do not prioritize or include special schools in their programs which indicates a potential service gap in this area. It is very rarely special schools are included in these programs even by the participated organizations. This means that most special schools in the study area might not have been screened in the past, but only a few have been covered. Additionally, according to the responses from the SSMs, their responses do not mean that they conducted eye health programs as they mentioned, rather, it could also mean that they responded randomly, leaving significant scope for recall or information bias. We agree that this is one of the limitations of the study and this has been mentioned in the limitations section.

c. I’d encourage authors to explicitly mention or clarify at the beginning of this section (and wherever relevant) that the statements presented within the themes are as expressed by the participants. Sometimes it may be confused with the author's opinion. For example, are the following statements based on what the data (participants' responses) suggests or a fact or opinion of the authors?

i. “Special SEH programmes are usually done as stand-alone programmes and hence they are rarely included in government or NGO sponsored activities.”
ii. “ … special SEH programmes require the parents to attend while the eye examination is being carried out.”
iii. “Most SEH programmes are conducted by community eye health workers, schoolteachers and vision technicians who are not trained to carry out a complete eye examination.”

Responses: These are very good points. Yes, these responses are presented as per the themes emerged from the participants response, not the authors interpretation.

d. Reading the description of the first barrier (low program coverage) for organisers, it feels like this theme is more about low priority (which then resulted in low coverage). This is my impression reading the description, not the full data. That’s why I reiterate my earlier comments about iterative refining and defining/naming of the themes and the importance of reporting on the investigator's roles in this process.

Response: This is a valid point. We have modified as suggested

e. I also note that the themes in the revised manuscript are different to the barriers listed in Box 2 of the original submission. Some of the previously mentioned barriers such as “no standard screening protocols” and “infrastructures are inaccessible” are not mentioned as barriers in the revised submission.

Response: These were not included in the revised manuscript as the data was reanalysed before submitting the revision. During the reanalysis these were not found to be part of the emergent themes. Hence, we did not include these in the revised manuscript. During the reanalysis, inaccessible facilities were found not as barriers rather these were suggested by the participants. These responses are listed in the table 4.

f. Line 267-268: “On the other hand, special schools seldom take the initiative in organizing eye health programmes.” If this is the organisers’ perception, were they probed on why they believed so?

Response: This was not probed further while this would have given a perspective on why schools did not take initiatives. Hence, we have included in the discussion part that the probable reasons for this could be other disabilities taking priorities over eye health and low awareness levels. The following has been discussed in the discussion section. “From the schools’ point of view, it is also possible that overall disability takes priority over eye health, and hence the schools also do not take initiative in organizing SEH programmes. This also shows a possible knowledge gap among the school management side.”

g. A lack of stakeholder engagement: were the organisers probed about the engagement or motivation of the school management? Considering the earlier part of the study on SSM and their potential role in organising eye health programs, it seems important to explore this aspect.

Response: Due to the limited time (3 months) frame from the conception till the end reporting of the study as part of MSc work, the data collection happened parallelly rather than first questionnaire responses and then the interviews which would have helped in including questions based on the responses from the SSM group. We also agree probing the participants would have given more information on this. This is one of the reasons why we suggested further research in which we would be able to incorporate the learnings from this study. We also have secured a grant to carry out a large-scale special school eye health program in which we intend to include these aspects while doing the data collection in the future research.

Discussion:
1. The discussion section as such is quite lengthy and needs to be a bit more concise.
2. Some of the statements in the discussion seem to better fit the results section or are repeated from the results section.

Response: Thank you for this input. While we agree that if the concise discussion would be better, we aimed to keep the discussion around the most important points, many of which cannot be ignored and hence we would like to keep most of these aspects included.

3. Some results are introduced in the discussion without a mention in the results section. For example:
a. Line 381-382: “They also felt studying with normal peers could improve their psychosocial well-being.”

Response: We re-looked at the data and have modified the statement in the discussion as follows, “ Although positive impact of inclusive education on psychosocial well-being is supported by studies from high-income countries (32), the evidence in LMICs is inadequate.(33) Additionally, this study did not explore to understand this factor.”
b. Line 400: “However, this was not reported by the SSM staff.” SSM’s responses relating to annual health check-ups covering eye examinations were not mentioned in the results.

Response: This is a very valid point and we have modified this statement after relooking at the data. “Although it was reported that the regular health check-ups in the schools included eye health, this could not be verified in this study. Hence, future research should prioritise assessing the frequency and ophthalmic content of general health check-ups at special schools, as well the competency levels of those who perform these examinations.”

4. Line 354-357: Does this mean that the children haven’t had any eye examinations in past? It is important to have an eye examination, but it does not need to be through a school eye health program.

Response: We felt that if the respondents knew that an eye examination is important, they would have mentioned about the referring the children or conducting vision screening in a bit more detail. However, none of them mentioned anything about the past referrals or eye examinations in details. This aspect will be considered in the future research.

5. The discussion around Vitamin A and its deficiency seems overstretched as this was how the survey questions were designed (MCQ without Vit A option!). Further, Gogate at el. (Gogate P, Soneji FR, Kharat J, Dulera H, Deshpande M, Gilbert C. Ocular disorders in children with learning disabilities in special education schools of Pune, India. Indian J Ophthalmol. 2011 May-Jun;59(3):223-8.) shows that about 2% of bilateral vision impairment is due to vitamin A deficiency in children with learning disabilities in special education schools of Pune, India.

Response: This is a good point and we do agree it has been stretched and we have shortened the discussion part based on the suggestion as following, “ Most participants said poor nutrition could cause eye problems, but none of the participants mentioned vitamin A deficiency (VAD). Perhaps including VAD specifically in the questionnaire MCQs would have impacted the responses on the causes reported. Few studies have reported VAD among children with SEN.(25, 30) This also warrants active inclusion of health promotion as part of special SEH programs in the future.”

Limitations:
1. While the sample size limitation is mentioned, authors need to clearly mention its implication i.e. the generalisability of findings. Further, the small number of interviews conducted needs to be mentioned with its implications in data saturation and confidence in the completeness of the barriers unearthed.

Response: We have included these aspects into the limitation section as suggested.

Language and formatting:
Some suggestions to improve the language and formatting of the manuscript:
1. Break down the long and complex sentences with multiple concepts. For example, the first sentence in the abstract.

Response: We have made these changes as suggested throughout the manuscript. Thank you for the suggestion.
2. Minimise the use of non-standard abbreviations
3. Consistent use of decimal points (30.8% vs 31%) and terms (eye health providers vs organisers)

Response: This has been made uniform. Numbers have been made as decimals (30.8% etc) throughout the manuscript

4. Careful re-examination of the numbers and proportion reported. E.g.,
a. Line 359: “…only less than a third of participants were aware of this condition.” Table 2 shows 23% which is less than a quarter of the participants.

Response: This has been corrected and reconfirmed
b. Line 362: “All participants said poor nutrition…” Table 2 shows 12 of 13 participants for this response.

Response: This has been changed to most participants.
