# Supplementary material for: Eye health knowledge, attitude, and practice among special school managers and barriers to eye health programmes in special schools in Hyderabad, India
Source: PLOS Glob Public Health. 2024 Aug 28;4(8):e0002124. doi: 10.1371/journal.pgph.0002124 (PMC11356432; doi:10.1371/journal.pgph.0002124)
Supplement: Attachment — Submitted filename: PGPH-D-23-01037_Response to the reviewers comments.docx [file pgph.0002124.s009.docx]

Response to reviewer comments

Dear Reviewer,

We would like to take this opportunity to thank you for your insightful suggestions, corrections and comments on our manuscript. We have addressed all the comments in the manuscripts. We have also considered all the minor corrections as recommended by you in the manuscripts. We have also taken the science language editor’s help to review the manuscript and edited the language as suggested.

1. Were these programme organizers chosen from amongst all eye care providers (including private providers) or just from amongst the NPO sector?

Response: These organizers were chosen from the group of organizations that are from both not for profit as well as government run organizations. The private sector institutions do not involve in community eye care and therefore, these were not included in the study.

2. Noting the limitations it may be necessary to include “As the study was negatively impacted by the Covid pandemic, further research with wider representation is needed to plan comprehensive eye health programmes for children with SEN”

Response: Thank you for your suggestion and this point makes it much better. Therefore, we have changed it in the manuscript as suggested.

3. As per Reviewer 6, you need to explain why SSM were more appropriate to participate as opposed to teachers.

Response: The currently study does not argue that the SSM were more appropriate to participate, rather it is one of the main limitations of the study. We further added in the limitations section that including class teachers rather than the heads of schools would have been better as the class teachers interact more with the children than the heads of schools. Additionally, we have also mentioned the reasons for including SSMs instead of teachers as it was carried out online and the contact details available online were only of the heads of schools.

4. Rephrase: Representatives of special schools identified from a local tertiary eye care centre database completed the online questionnaire.

Response: Thank you for this suggestion and we have rephrased the sentence as suggested.

5. What is a “valid” contact detail as opposed to one that will be considered “invalid”.

Response: We mean to imply those whose contact details were still working while being contacted.

6. Were these developed and piloted?

Response: Yes, the questionnaire was piloted prior to the start of the study. However, we did mention that the pilot was carried out among the peer group of the PI, which included a heterogenous background. Hence there is a possibility of varying degree of understanding of the questions which has been mentioned in the limitations of the study.

7. The questionnaire included open ended questions as opposed to open ended “text boxes” The text boxes are included for the responses to be inserted, but your questions were “open-ended. Please rephrase throughout.

Response: Thank you for this suggestion. We have rephrased this as open-ended questions as suggested.

8. This represents only 19% of SES in the study area. This is a study limitation.

Response: Thank you for pointing this out. Yes, the overall participation is not the complete representation of the all the schools in the area and this has been mentioned in the limitation.

9. these seem more like conditions needed for children to attend regular schools as opposed to “reasons WHY they should/should not attend regular schools. Please review the responses carefully and rephrase.

Response: Thank you for the suggestion. We have rephrased it as following, “Close to 2/3^rd^ of the participants (61.54%, n=8/13) mentioned that children with VI can interact normally with their peers and comprehend like those without VI. In addition they will also feel motivated being with their normal peers. Hence, attending mainstream schools would benefit them. On the contrary, 1/3^rd^ of the participants felt, those with VI will benefit from special schools as they will receive additional support and care in these schools.”

10. This merely covers “lack of initiative”. What does “lack of ownership mean as it is not covered in the paragraph below?

Response: Thank you for the suggestion. We have changed it to lack of initiative as suggested.

11. This paragraph appears to highlight “stakeholder related challenges”

Response: Thank you for the suggestion. This has been changed as suggested
