# Supplementary material for: Eye health knowledge, attitude, and practice among special school managers and barriers to eye health programmes in special schools in Hyderabad, India
Source: PLOS Glob Public Health. 2024 Aug 28;4(8):e0002124. doi: 10.1371/journal.pgph.0002124 (PMC11356432; doi:10.1371/journal.pgph.0002124)
Supplement: Attachment — Submitted filename: PGPH-D-23-01037_Response to the reviewers comments.docx [file pgph.0002124.s011.docx]

Response to reviewer comments

Dear Reviewer,

We would like to take this opportunity to thank you for your insightful suggestions, corrections and comments on our manuscript. We have addressed all the comments throughout the manuscripts and have highlighted the changes. This was a great learning experience.

1. You need to use the term “high”, as you are not comparing to another group in this sentence

Response : Changed as suggested

2. You need to always write in a manner that shows that these are responses as reported by the participants and not your thoughts.

Response : This is a very good input and we have changed the narrative as suggested by you. Thank you for this suggestion.

3. Were these responses as this reported by the study participants? If so, please write it in that manner. If not, then it belongs in the Discussion and not in Results

Response: This is a very good suggestion. Thank you for this suggestion. We have made the changes as suggested as below.

The organizers mentioned that special schools require a skilled workforce trained for the assessment of children with SEN, low vision assessment, and prescription of spectacles and low vision devices (LVDs). Additionally, they mentioned that most SEH programmes are conducted by community eye health workers, schoolteachers, and vision technicians who are not trained to perform a complete eye examination. They also mentioned that having a more skilled workforce, including optometrists or ophthalmologists, would incur additional costs. They also mentioned that in some circumstances, such cadres are unwilling to participate in the SEH programmes due to long-distance travel and limited financial benefits. Therefore, most special SEH programmes are conducted by unsupervised optometry trainees.

4. Again, report as a result

Response: Thank you for this suggestion. We have made the following changes.

The organizers mentioned that a complete eye examination for children with SEN requires expensive equipment, increasing the demand for financial resources. They also stated that only dry refraction can be performed on the school premises, and children require a referral for cycloplegic refraction. The organizers also claimed that poor referral uptakes among this group make it difficult to provide effective refractive error correction via the SEH programmes alone.

5. Clearly state who were these suggestions made by

Response: Thank you for this suggestion. The following change has been made.

the two sub-themes of existing good practices observed in the included special schools and suggestions from the SEH programme organizers, for improved service provision are summarized in Table 4.
